# Lipid-donor-anchored genome mining uncovers dioxanopeptins, antibacterial lipopeptides with a 1,3-dioxane functionalized polyunsaturated lipid tail

**DOI:** 10.1039/d6sc00003g

**Published:** 2026-02-27

**Authors:** Ying Chen, Yunsheng Chen, Hao Xiang, Changqi Luo, Jiaqi Duan, Kun Hu, Xiaohong Zheng, Jing Liu, Yongbo Xue, Yi-Ming Shi

**Affiliations:** a School of Pharmaceutical Sciences (Shenzhen), Sun Yat-sen University Shenzhen China xueyb@mail.sysu.edu.cn; b State Key Laboratory of Quantitative Synthetic Biology, Center for Synthetic Biochemistry, Shenzhen Institute of Synthetic Biology, Shenzhen Institutes of Advanced Technology, Chinese Academy of Sciences Shenzhen China ym.shi@siat.ac.cn; c State Key Laboratory of Phytochemistry and Natural Medicines, Kunming Institute of Botany, Chinese Academy of Sciences Kunming China; d Equipment Public Service Center, South China Sea Institute of Oceanology Guangzhou China; e Department of Natural Products in Organismic Interactions, Max Planck Institute for Terrestrial Microbiology Marburg Germany; f University of Chinese Academy of Sciences Beijing China

## Abstract

Non-ribosomal lipopeptide antibiotics derive much of their antibacterial performance from the structure of their lipid tail, yet genome mining remains largely peptide-centric and rarely prioritizes lipid donor formation as an entry point. Herein, we establish a lipid-donor-anchored discovery strategy using *pfa*-type polyunsaturated fatty acid synthase (PUFAS) core genes as the genomic anchor for long polyunsaturated acyl donors, followed by filtering for co-localization of non-ribosomal peptide synthetase (NRPS) assembly lines. This approach delineated 60 curated PUFAS–NRPS biosynthetic gene clusters, including a previously unassigned *dxp* family, which is distributed across Proteobacteria. Cultivation of *Chitinimonas koreensis* DSM 17726 enabled the isolation of dioxanopeptins, a new set of non-ribosomal lipopeptides featuring a long polyunsaturated lipid tail further functionalized by a pyruvyl-derived 1,3-dioxane ketal motif. Recombinant DxpH catalyzed the *in vitro* conversion of dedioxanopeptin to dioxanopeptin using phosphoenolpyruvate as the pyruvyl donor, establishing pyruvylation as a post-assembly tailoring step. Dioxanopeptins displayed selective activity against methicillin-resistant *Staphylococcus aureus*, and their antibacterial phenotype correlated with disruption of proton motive force homeostasis and ATP production. Removal of the pyruvyl-derived ketal markedly diminished potency, highlighting its contribution to antibacterial activity. Together, these results expand the chemical space of lipopeptides and illustrate how lipid-donor-anchored genome mining can complement NRPS-focused strategies to access functionally differentiated antibiotic scaffolds.

## Abstract 2

Lipid-donor-anchored genome mining uncovers dioxanopeptins, a series of lipopeptides with a 1,3-dioxane functionalized polyunsaturated lipid tail. They selectively inhibit MRSA by disrupting proton motive force homeostasis and ATP production.

## Graphical abstract

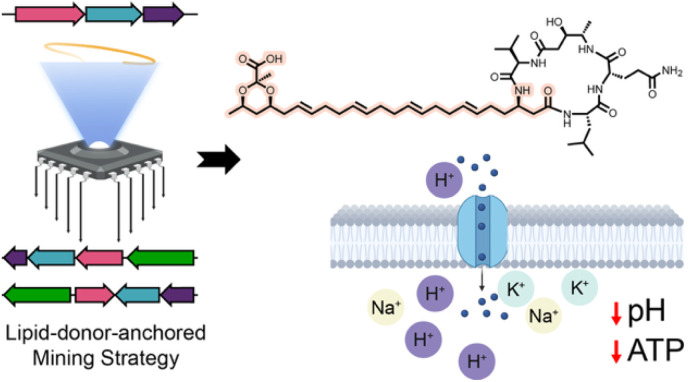

## Introduction

Non-ribosomal lipopeptide antibiotics, exemplified by daptomycin and polymyxins, are bacterial natural products in which a linear or cyclic peptide is covalently linked to a lipid tail, creating an amphiphilic scaffold.^1^ Lipopeptides often exert their effects by targeting the bacterial envelope: the hydrophobic acyl moiety inserts into lipid bilayers, while the peptide headgroup interfaces with surface components, such as phospholipid headgroups and lipid-linked cell-wall precursors.^2^ This makes the lipid moiety a major determinant of membrane partitioning and, consequently, antibacterial potency and spectrum.^3^

As membrane insertion is frequently the entry point to activity, small structural changes in the lipid moiety, including chain length, branching, saturation, and functionalization, can dramatically influence the potency, spectrum, selectivity, and mode of action. As a trend, shorter lipid tails can favor efficient insertion into bacterial membranes, whereas longer and more hydrophobic tails can enhance stable membrane binding and oligomerization, often shifting antibacterial potency and selectivity. Branched-chain fatty acyl groups alter how the molecule packs into bilayers: even a methyl branch can introduce packing defects and modulate membrane disorder and pore-forming propensity.^4^ Unsaturated bonds likewise introduce conformational constraints that reshape insertion geometry and self-assembly, thereby affecting biological outputs.

**Fig. 1:**
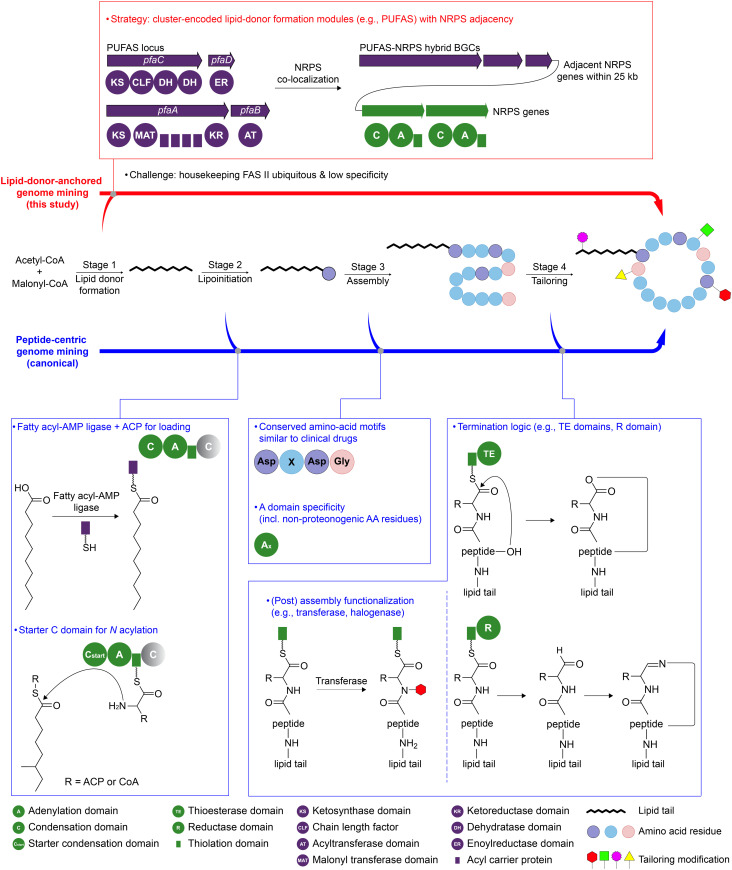
Lipid-donor-anchored genome mining for lipopeptide discovery. Schematic comparison of genome-mining entry points mapped onto four stages of lipopeptide biosynthesis.

Although lipid-tail features strongly modulate lipopeptide bioactivity, genome mining is still largely peptide-centric, prioritizing biosynthetic gene clusters (BGCs) primarily by non-ribosomal peptide synthetase (NRPS) domain architecture^5–8^ and therefore providing limited leverage over the lipid-tail chemical space. A key bottleneck is that, for many canonical lipopeptides, the acyl donor is supplied by ubiquitous housekeeping type II fatty acid synthase (FAS II),^9^ so lipid donor formation often lacks a distinctive, cluster-encoded genomic beacon for prioritizing unusual lipid tails. We frame lipopeptide genome mining around four biosynthetic checkpoints: lipid donor formation, lipoinitiation, assembly, and tailoring (stages 1–4, Fig. 1). Currently, most established genome mining workflows tend to focus on BGC signatures in stages 2–4. Lipoinitiation is frequently exploited, leveraging fatty acyl-AMP ligase/ACP systems^10^ and starter condensation (C) domains^11^ that couple an acyl group to the first amino acid, but is less likely to indicate which non-canonical lipid donors are supplied. Non-ribosomal peptide synthetase–polyketide synthase (NRPS-PKS) assembly is then prioritized *via* module architecture and A-domain specificity prediction,^6^ which is powerful for proposing peptide cores. Finally, tailoring^11^ (*e.g.*, thioesterase domain- & terminal reductase domain-mediated macrocyclization or reductive offloading; modification mediated by halogenases and glycosyltransferases) can guide diversification. Consequently, the lipid-tail dimension, despite its outsized functional importance, remains comparatively underexploited as a genome-mining entry point.

Given that lipid-tail features can drive membrane engagement and bioactivity, we reasoned that a discovery strategy that explicitly prioritizes lipid-donor formation might better enrich for functionally differentiated lipopeptides. Here, we shifted non-ribosomal lipopeptide genome mining from a peptide-centric to a lipid-donor-anchored strategy, using cluster-encoded lipid-donor formation genes as the primary entry point and NRPS co-localization as a secondary filter. As a proof-of-concept, we focused on polyunsaturated fatty acid synthase (PUFAS) core genes (*pfaA-D*) as the lipid-donor machinery, which is largely BGC-encoded and thus provides a genomic signature for mining new classes of lipopeptides. This led to the discovery of the *dxp* (dioxanopeptin) BGC family, predominantly found in Proteobacteria. The Ck-*dxp* BGC from *Chitinimonas koreensis* DSM 17726 was expressed under standard laboratory conditions and produced unusual lipopeptides, termed dioxanopeptins (1–6), bearing a long unsaturated lipid tail with a 1,3-dioxane ring, putatively installed by a pyruvyltransferase. Dioxanopeptins exhibited selective activity against methicillin-resistant *Staphylococcus aureus* (MRSA) by dissipating the proton motive force (PMF), thereby compromising ATP production.

## Results and discussion

### Discovery of *dxp* gene cluster family in Proteobacteria by targeting PUFA pathways

**Fig. 2:**
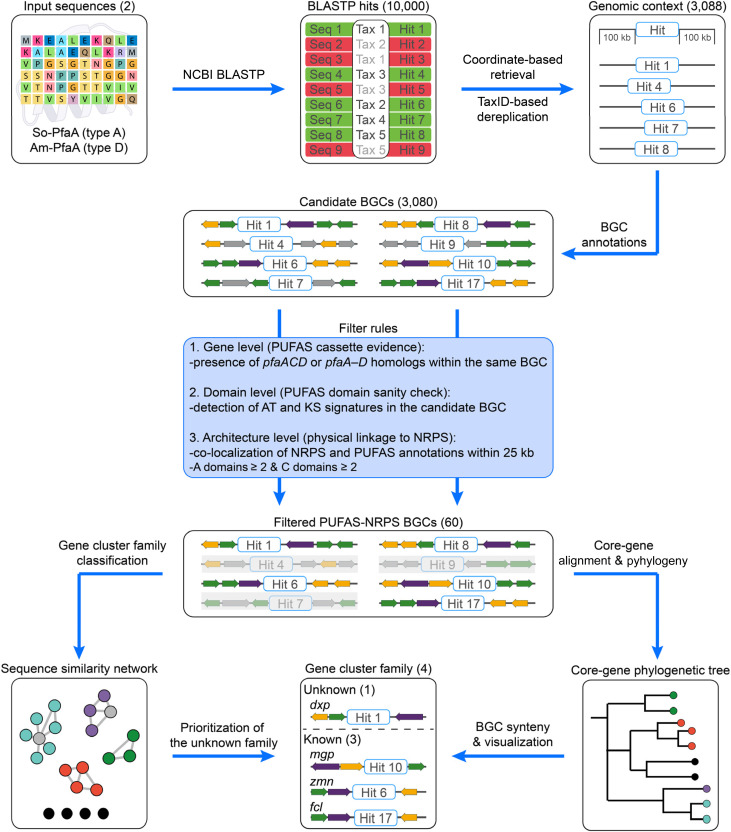
Lipid-donor-anchored lipopeptide genome-mining workflow, illustrated using *pfa* genes as a proof of concept. Representative Shewanella-type (So-*pfa*, type A) and Aureispira-type (Am-*pfa*, type D) PfaA proteins were used as BLASTP queries to retrieve genomic neighborhoods (±100 kb) around each hit, followed by TaxID-based dereplication and antiSMASH annotation to identify candidate BGCs. Candidate PUFAS–NRPS hybrids were retained using rule-based filters. The resulting 60 BGCs were grouped by the BiG-SCAPE sequence similarity network against MIBiG reference BGCs, prioritizing the unknown *dxp* family for experimental characterization. Core-gene phylogeny (*pfaA*, *pfaC*, *pfaD*, and the co-localized NRPS component) and synteny analyses were used to delineate and visualize the *dxp*, *fcl*, *zmn*, and *mgp* families. Since the NCBI BLAST service returns a maximum of 5000 target sequences per search, the So-PfaA and Am-PfaA BLASTP results were merged (up to 10 000 hits). In the BLASTP results, green denotes retained representatives after dereplication and red denotes hits flagged as redundant. See the SI for methodological details.

PUFAS in bacteria is a PKS/FAS-like multienzyme complex typically encoded by a *pfa* locus (*pfaA-D*, Fig. 1), in which *pfaA* carries the core KS–MAT–ACP repeats–KR architecture, *pfaB* encodes a stand-alone AT, *pfaC* contains KS-CLF–DH modules, and *pfaD* encodes an ER. Comparative genomics has grouped *pfa* loci into types A–T.^12^ To establish a high specificity genome mining entry point for PUFAS machinery, we selected two representative *pfa* loci^13^ (Fig. 2): the Shewanella-type (So-*pfa*, type A) and Aureispira-type (Am-*pfa*, type D), which differ structurally but share the overall catalytic logic. So-*pfa*, the canonical *pfa* locus, includes *pfaA-D*, with the KR domain embedded in So-PfaA and the AT domain as a stand-alone So-PfaB. In contrast, Am-*pfa* has a rearranged structure, with the KR region separated from Am-PfaA as a stand-alone enzyme and the AT region fused to Am-PfaC, which is characteristic of certain non-canonical PUFAS. These So-*pfa* and Am-*pfa* loci capture both canonical and rearranged PUFAS architectures, improving homology-based retrieval across divergent *pfa* loci. The BGCs were then filtered by co-localization of an NRPS assembly line within 25 kb of the *pfa* locus, enriching for candidate lipopeptide BGCs with PUFAS machinery adjacent to NRPS genes.

**Fig. 3:**
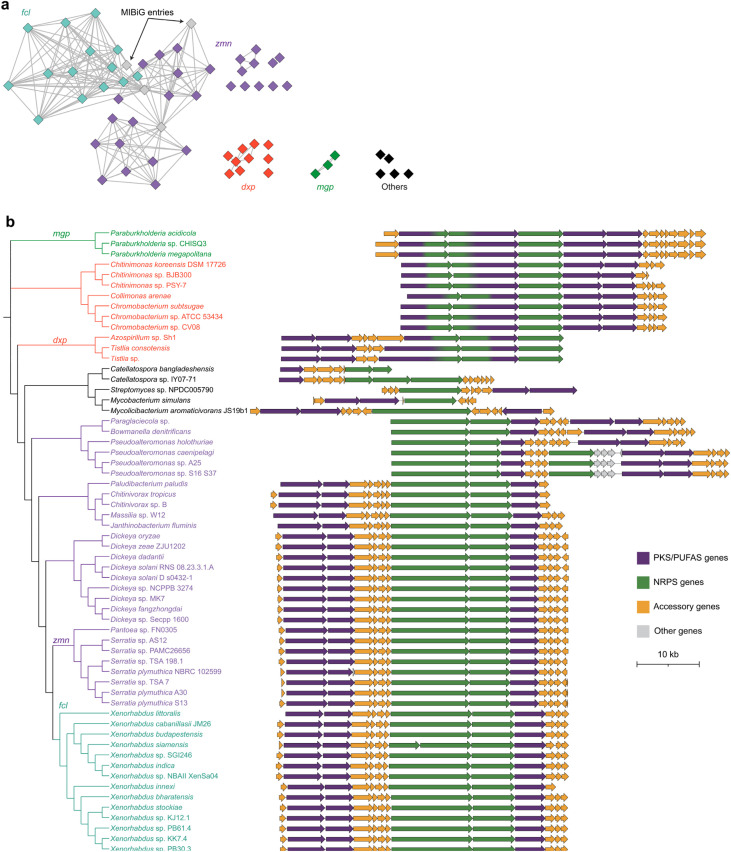
Sequence similarity network and maximum-likelihood core-gene phylogeny of the 60 curated PUFAS–NRPS BGCs. (a) BiG-SCAPE sequence-similarity network showing four major gene cluster families: three known families (*fcl*, *zmn*, and *mgp*; representative chemical structures of the products are shown) and the previously untapped *dxp* family. (b) Phylogeny of the PUFAS–NRPS BGC core genes, inferred from a concatenated alignment of *pfaA*, *pfaC*, and *pfaD*, together with the co-localized NRPS component. The tree is used to delineate and visualize family-level clades and to order the corresponding BGC maps (see also Fig. S1 for synteny).

By doing so, we identified 60 candidate BGCs in which a *pfa*-type PUFAS occurs adjacent to an NRPS assembly line. We then used BiG-SCAPE^14,15^ to construct a BGC similarity network against the reference BGCs in the MIBiG database,^16^ which classified these BGCs into four major families (Fig. 3a). This includes three previously described families, *fcl* (fabclavine biosynthesis),^17^*zmn* (prezeamine biosynthesis),^18^ and *mgp* (megapolipeptin biosynthesis),^19^ as well as a previously unrecognized family without linkage to any MIBiG entries, which we term *dxp*. We then performed phylogenetic and synteny analyses to delineate and visualize the family-classified BGCs (Fig. 3b and S1). Our analysis was consistent with earlier reports showing that *fcl* and *zmn* clusters were primarily associated with genera such as *Xenorhabdus*, *Serratia*, and *Dickeya*,^20–22^ whereas the *mgp* family appears largely restricted to *Paraburkholderia*.^19^ In contrast, synteny analysis revealed the *dxp* family as a distinct and comparatively restricted lineage, represented by *Chitinimonas*, *Chromobacterium*, *Tistlia*, *Azospirillum*, and *Collimonas* within Proteobacterial genomes.

**Fig. 4:**
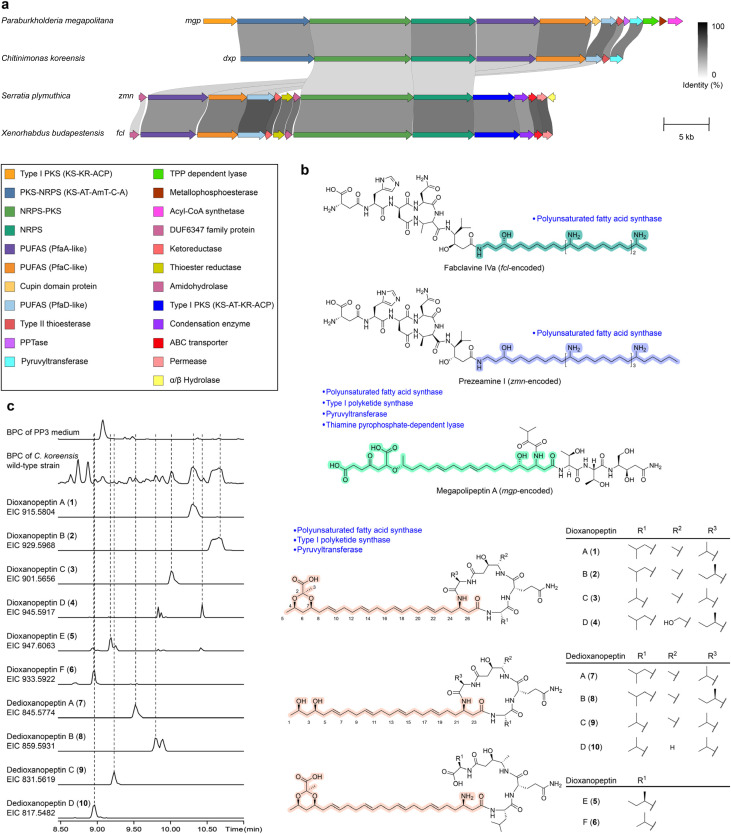
Comparison of genome-mined PUFAS–NRPS gene cluster families and representative hybrid products. (a) Comparison of the *fcl*, *zmn*, *mgp*, and *dxp* BGCs. Links connect similar genes across clusters and are shaded by sequence identity. (b) Structures of representative products from the known families (fabclavine IVa, prezeamine I, and megapolipeptin A) and the *dxp*-encoded dioxanopeptins (1–6) and dedioxanopeptins (7–10). Lipid-derived moieties assembled by the dedicated lipid-tail machinery are highlighted, and family-specific tailoring enzymes are indicated. (c) BPC and EICs for the indicated [M + H]^+^ ions of dioxanopeptins and dedioxanopeptins produced by *C. koreensis* DSM 17726 cultivated in PP3 medium.

In the *fcl*^17^ and *zmn*^18^ pathways (Fig. 4a), the PUFAS-like system is repurposed to build a long polyamino alcohol chain (Fig. 4b) *via* recruitment of a modular aminotransferase, together with a stand-alone KR and a thioester reductase. In parallel, a PKS–NRPS assembly line produces a mature peptidyl thioester tethered to a carrier protein, which is subsequently condensed with the polyamino alcohol chain to yield fabclavines and prezeamines. The specialized polyamino alcohol moiety is appended to the C-terminus of the peptide scaffold. The *mgp* family has distinct auxiliary functions (Fig. 4a), including an unusual stand-alone cupin family enzyme and PfaD homologs with an additional ACP domain.^19^ Notably, the *mgp* family encodes a pyruvyltransferase, proposed to act together with a thiamine pyrophosphate-dependent lyase to assemble the 4-oxoheptanedioic moiety that decorates the polyunsaturated lipid chain. The specialized lipid thus constitutes the N-terminal starter unit of the megapolipeptin scaffold (Fig. 4b).

By contrast, the *dxp* family encodes a compact PUFAS–NRPS core (Fig. 4a), in which DxpD (PfaA homolog) carries four tandem ACP domains and DxpF (PfaD homolog) carries a single ACP. This feature matches the *mgp* family but differs from the *fcl* and *zmn* families. In addition, the *dxp* family encodes fewer accessory genes flanking the core assembly line, yet retains a putative pyruvyltransferase, suggesting a distinct lipid-tail functionalization.

To link genotype to chemotype, we selected three publicly accessible DSMZ strains harboring representative *dxp* BGCs, including *C. koreensis* DSM 17726, *Chromobacterium subtsuga* DSM 17043, and *Tistlia consotensis* DSM 21585, for cultivation and LC-HRMS screening. To facilitate targeted detection and interpret structural variation, we profiled NRPS A-domain substrate specificity for the prioritized BGCs using three complementary predictors, NRPStransformer^23^ and PARAS/PARASECT,^24^ in parallel. We recorded the top-three candidates for each A domain (Table S1). Among the three tested strains, extracts of *C. koreensis* DSM 17726 reproducibly showed a series of peaks with late retention times (1–10, Fig. 4c), consistent with a highly hydrophobic scaffold. Also, MS/MS spectra displaying neutral losses correspond to valine, leucine, and glutamine residues (Fig. S4–S17). Isotope feeding experiments of d_8_-l-valine and ^13^C/^15^N-labeled media further supported a molecular composition of six nitrogens and 43–49 carbons. Five out of six nitrogens are attributable to four A-domain-incorporated amino acid residues (four backbone amide nitrogens plus one side-chain amide nitrogen from an amide-containing residue, *e.g.*, glutamine), and the remaining one is attributable to an amino transferase domain-installed amino group. The 43*–*49 carbon count exceeds what would be expected from the peptide alone. Collectively, these data suggest a lipopeptide family bearing an extended polyunsaturated lipid tail, as anticipated from the Ck-*dxp* BGC. This motivated scale-up fermentation and subsequent isolation and structural elucidation of compounds 1–10.

### Structure elucidation of dioxanopeptins

The structures of dioxanopeptins (Fig. 4b) were established by combined NMR spectroscopy (Fig. S2 and Table S3), MS/MS analysis (Fig. S4–S17), isotope-labeling experiments (Fig. S4–S9), bioinformatic analysis (Fig. S3 and S19–S21), and Marfey's derivatization (Fig. S18), showing that dioxanopeptins are cyclic lipopeptides bearing a long polyunsaturated lipid tail with a 1,3-dioxane motif. Detailed NMR assignment is provided in the SI. The ^1^H NMR and HSQC spectra suggest a regularly arranged unsaturated fatty chain in which adjacent double bonds are separated by two CH_2_ units. The ^13^C chemical shifts of the allylic methylene carbons adjacent to the olefins cluster around *δ*_C_ 32 ppm. According to previous studies,^25,26^ allylic methylenes next to *trans* double bonds typically resonate at 32–34 ppm, whereas those next to *cis* double bonds appear at 27–29 ppm. Therefore, we assigned the polyunsaturated double bonds as *trans*. We further employed Marfey's method to confirm that leucine and glutamine are l-configured, while valine is d-configured (Fig. S18). These experimental results are consistent with the amino acid stereochemistry predicted by the C/E domain annotations. The configuration of the alanine residue, which was extended by a polyketide unit, was assigned to be l by C domain prediction (Table S6).

The stereochemistry of C-4, C-7, and C-41 was tentatively proposed to be 4*R*, 7*S*, and 41*R*, respectively, based on the analysis of the catalytic subdomain of KR (KR_*C*_) sequences.^27^ In the resulting phylogeny, the Ck-DxpD KR_*C*_ clustered within the B-type KR clade, whereas the Ck-DxpB KR_*C*_ grouped with the A-type KR clade, supporting type-specific stereoselectivity assignment (Fig. S3). In particular, to provide NMR evidence for the C-41 stereochemical assignment, we recorded a PSYCHEDELIC spectrum^28^ on dioxanopeptin B (higher abundance) to resolve the congested multiplet and extract ^3^*J*_H39–H41_ = 7.5 Hz (Fig. S2), which indicates an *anti*-relationship between H-39 and H-41 and is in agreement with the C-41 assignment inferred from the KR_*C*_ phylogeny. To determine the configuration at C-25, we performed DFT-GIAO NMR calculations^29^ on truncated structural models. The computed shifts for the 25*R* isomer (1T-a) showed better agreement with the experimental ^13^C and ^1^H data than those for the 25*S* isomer (1T-b), as reflected by the higher *R*^2^ and lower mean absolute error/corrected mean absolute error values. Therefore, DP4+ analysis^30^ strongly supported the 25*R* isomer over 25*S*, giving a DP4+ probability of 100.00% (Fig. S2).

The structures of dioxanopeptins B (2) and C (3) were confirmed by comparative NMR analysis with dioxanopeptin A (1). Dioxanopeptins E (5) and F (6), with shorter retention times in the HPLC chromatogram, showed MS/MS fragmentation patterns identical to 2 and 1, respectively, but had a +18 Da mass shift (Fig. S12 and S13). Therefore, 5 and 6 were assigned as corresponding hydrolytic linear congeners of 2 and 1.

We observed a series of compounds (7–10) that eluted earlier in the HPLC chromatogram than dioxanopeptins A–C (1–3). The MS/MS spectra of 1–3 and 7–10 showed conserved fragmentation for the peptide moiety, while diagnostic fragments of the lipid portion differed by −70 Da (C_3_H_2_O_2_, Fig. S14–S17). This mass deficit is consistent with the loss of a pyruvyl-like moiety, indicating that compounds 7–10 lack the pyruvyl-derived ketal.

### Proposed biosynthetic pathway and *in vitro* characterization of a pyruvyltransferase

**Fig. 5:**
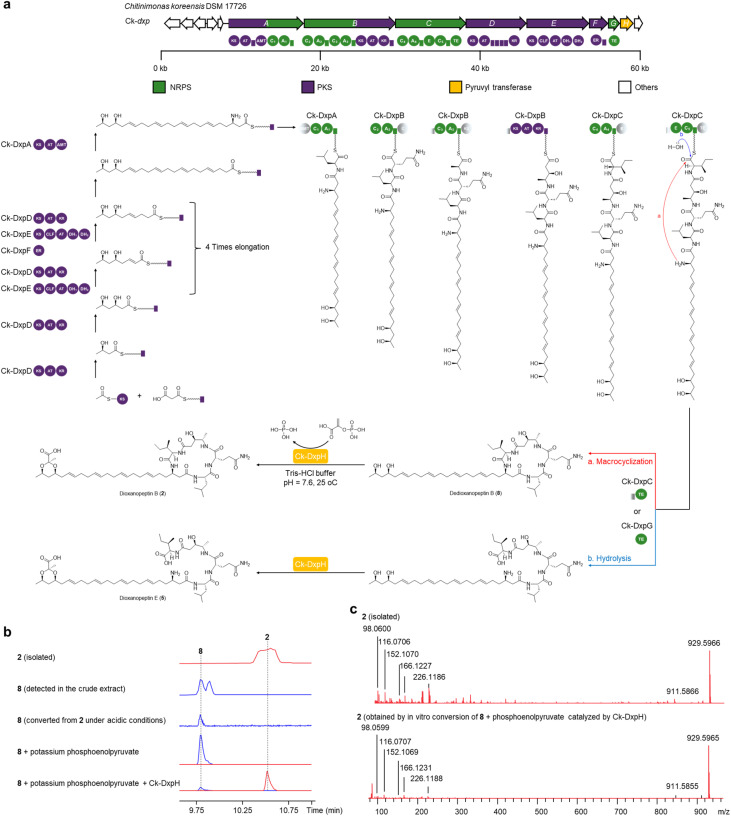
Proposed biosynthetic pathway for dioxanopeptins and *in vitro* characterization of the pyruvyltransferase Ck-DxpH. (a) Proposed *dxp* biosynthetic pathway, exemplified by dioxanopeptins B (2) and E (5). The pyruvyl-free intermediate dedioxanopeptin B (8) is proposed as the substrate for DxpH-catalyzed pyruvyl ketal formation to yield 2, which is supported by *in vitro* assays. (b) EICs showing the *in vitro* conversion of 8 to 2 catalyzed by recombinant Ck-DxpH using potassium phosphoenolpyruvate as the pyruvyl donor (EIC of 2, red; EIC of 8, blue). (c) Comparison of MS/MS spectra of the isolated standard and the enzymatic product of 2.

Based on the structures of dioxanopeptins (1–10, Fig. 4b) and the domain architecture of the Ck-*dxp* BGC (Fig. 5a), we propose that DxpDEF iteratively assemble the long polyunsaturated lipid chain on ACP domains, initiating from an acetyl-CoA starter and extending with malonyl-CoA units. The KR in DxpD is predicted to generate β-hydroxyacyl intermediates during chain extension. Iterative elongation is expected to yield a transient β,δ-diol intermediate. Next, alternating partially and fully reductive cycles take place. In a partial reduction cycle, the KR in DxpD reduces the β-ketoacyl intermediate to a β-hydroxyacyl thioester, which is then dehydrated by the DH in DxpE (homologous to FabA, Fig. S19 and S20) to give an α,β-enoyl thioester, thereby installing a *trans* double bond.

Canonical PUFASs involve DH_FabA_ dehydratase/isomerase chemistry for *cis* double-bond installation.^31^ To contextualize this unusual geometry biosynthetically, we further analyzed the PUFA-associated DH domains. Ck-DxpE contains two tandem hotdog-fold DH domains. Based on their order, we refer to these regions as Ck-DxpE DH_1_ (N-terminal) and Ck-DxpE DH_2_ (C-terminal). Sequence comparison (Fig. S21) indicates that DH_1_ is degenerate, as it lacks an eight-residue segment within the conserved catalytic region (corresponding to positions 70–88 in FabA and FabZ) and does not retain the canonical His–acidic residue dyad, whereas DH_2_ preserves an intact catalytic segment and the His–acidic dyad, consistent with a catalytically competent FabZ-like DH domain. We therefore focused our phylogenetic and active-site analyses on Ck-DxpE DH_2_ as the likely functional dehydratase. Similarly, MgpF contains two DH domains, of which DH_1_ appears to degenerate, whereas DH_2_ retains the canonical catalytic active sites. Phylogenetically, Ck-DxpE DH_2_ falls within the FabA clade and clusters with the DH_2_ from the *mgp* family. However, targeted sequence comparison against UniProt-reviewed FabA and FabZ references shows that Ck-DxpE DH_2_ lacks residues associated with FabA-type isomerization and instead carries a FabZ-like catalytic signature at the His–acidic-residue pair (FabA: His/Asp; FabZ: His/Glu),^32^ which is consistent with DH activity that yields *trans*-2-enoyl intermediates without Δ^2^ to Δ^3^*cis* isomerization. Taken together, these analyses are consistent with the *trans* assignment by NMR and provide a plausible mechanistic rationale for why the *dxp* PUFAS–NRPS system may deviate from the more typical *cis*-PUFA outcome.

In the subsequent full-reduction cycle, the newly formed β-ketoacyl intermediate undergoes KR (DxpD)- and DH (DxpE)-mediated processing followed by enoyl reduction by ER (DxpF) to complete a fully reduced two-carbon extension. Three additional repetitions of this alternation account for the observed polyunsaturated lipid chain.

The 22-carbon lipid is then passed to the downstream PKS–NRPS machinery. A PKS extension associated with DxpA is proposed to generate a β-keto group, which is then converted to a β-amino functionality by the embedded aminotransferase domain, yielding a 24-carbon polyunsaturated amino lipid. DxpA and DxpB catalyze the sequential incorporation of l-leucine/l-valine, l-asparagine, and l-alanine/l-serine/glycine, and the PKS module in DxpB installs a β-hydroxyacyl extender unit. The terminal residue is incorporated by the first module of DxpC and varies among valine/isoleucine, followed by epimerization to the d-configuration. Finally, macrocyclization or hydrolytic off-loading is proposed to be catalyzed either by the TE domain of DxpC or by the stand-alone TE DxpG, yielding the cyclic dedioxanopeptins (7–10), in which the amino group on the lipid chain is involved in ring closure, as well as in the formation of linear products.

The presence of a 1,3-dioxane ring suggests the formation of the dihydroxyl group on the polyunsaturated fatty acyl chain. We therefore propose that DxpH, a putative pyruvyltransferase, installs a pyruvyl ketal onto dedioxanopeptins (7–10) to furnish the dioxane motif of dioxanopeptins (1–6). To test this conversion, we first chemically removed the pyruvyl-like moiety from dioxanopeptin B (2) under acidic conditions to afford dedioxanopeptin B (8). We then performed *in vitro* assays using a recombinant Ck-DxpH protein (Fig. S23). Incubation of Ck-DxpH with 8 and potassium phosphoenolpyruvate showed the formation of 2, as confirmed by LC-HRMS and MS/MS comparison with authentic 2 (Fig. 5b and c). This indicates that pyruvylation occurs as a post-assembly modification. These results indicate that pyruvylation is a post-assembly tailoring modification in dioxanopeptin biosynthesis.

### Dioxanopeptins exert antibacterial activity through dissipation of PMF

In an attempt to determine the antibacterial activity of dioxanopeptins against multi-drug resistant (MDR) bacteria, we selected eight ESKAPE bacteria^33^ by using dioxanopeptins A–D (1–4) to confirm their antibacterial spectrum. We found that dioxanopeptins exhibited potent antibacterial activity against a panel of Gram-positive bacteria, such as MRSA and Vancomycin-Resistant Enterococci (VRE) with MICs ranging from 2 to 64 µg mL^−1^ (Tables 1 and S8). Notably, dioxanopeptins showed bactericidal activity against both *S. aureus* and *Enterococcus faecium*. However, we found that dioxanopeptins displayed weak or no inhibitory activity against Gram-negative bacteria (MICs reached 64 µg mL^−1^ or higher), but the efficiency was enhanced once the permeability of the outer membrane increased (Table 1). For example, the Δ*waaC* mutant strain of *Klebsiella pneumoniae* and LPS-deficient strain of *Acinetobacter baumannii* deficient in the outer membrane have shown higher sensitivity to dioxanopeptins compared to the wild type strains, with the MIC decreasing from 32 µg mL^−1^ to 4 µg mL^−1^ in the presence of dioxanopeptin A (1) to *A. baumannii*. Thus, we speculated that the outer membrane barrier hindered the diffusion of dioxanopeptins into the cytoplasm of Gram-negative bacteria. Additionally, although it is shown that dioxanopeptin has limited inhibitory activity against Gram-negative bacteria, we measured the growth dynamics of *Escherichia coli*, *K. pneumoniae*, *A. baumannii*, *Salmonella* Typhimurium, and *Pseudomonas aeruginosa* in the presence of dioxanopeptin. The concentrations of dioxanopeptin used to treat these species were 0.25×, 0.5×, 1×, and 2× MICs against *S. aureus*. We found that although the concentration of dioxanopeptin was far lower than the inhibition concentration, the overall biomass, such as *E. coli* and *S.* Typhimurium, was decreased, accompanied by up-regulation of metabolic activity in the late exponential phase and static growth phase (Fig. S24). Such results are consistent with previous studies in that bactericidal antibiotics are associated with metabolically active states.^34^ Taken together, these results suggest that dioxanopeptins are potent against MDR Gram-positive bacteria, whereas their activity against Gram-negative bacteria is limited by the permeability barrier of the outer membrane.

**Table 1:** Antibacterial activities of dioxanopeptins A–D (1–4) and dedioxanopeptin B (8)^a^

Organism	1		2		3		4		8
	MIC	MBC	MIC	MBC	MIC	MBC	MIC	MBC	MIC
*S. aureus* ATCC 29213	4	4	16	16	4	4	>64	>64	>64
*S. aureus* T144 (MRSA)	4	4	8	8	8	8	64	64	>64
*E. faecium* BM1405	8	8	8	8	8	8	16	32	n.a.
*E. coli* ATCC 25922	>64	>64	>64	>64	>64	>64	>64	>64	>64
*E. coli* B2	>64	>64	>64	>64	>64	>64	>64	>64	n.a.
*A. baumannii* 7-2	>64	>64	>64	>64	32	>64	64	>64	n.a.
*A. baumannii* 7-2 LPS deficiency	2	8	1	8	4	32	16	32	n.a.
*K. pneumoniae* ATCC 43816	>64	>64	>64	>64	>64	>64	64	>64	n.a.
*K. pneumoniae* ATCC 43816 Δ*waaC*	64	>64	64	>64	16	>64	64	>64	n.a.
*P. aeruginosa* PAO1	>64	>64	>64	>64	64	>64	64	>64	n.a.

a MIC and MBC, minimal inhibitory concentration and minimal bactericidal concentration, µg mL^−1^; MRSA, methicillin-resistant *S. aureus*; n.a., not applicable.

**Fig. 6:**
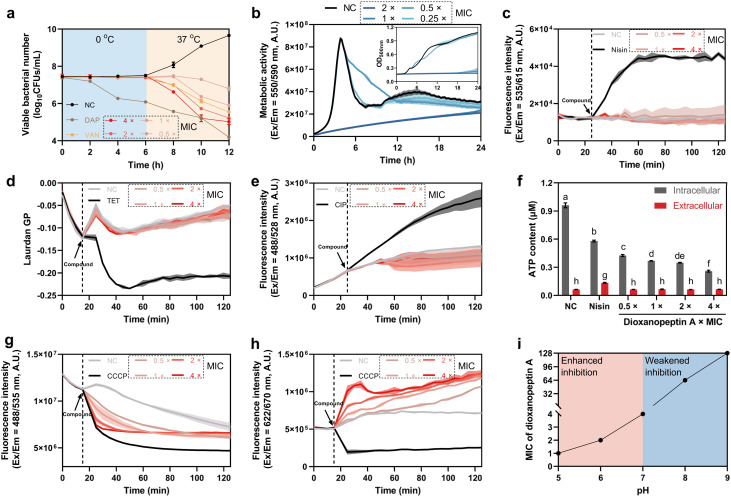
Dioxanopeptin impairs PMF resulting in a reduced production of ATP. (a) Bactericidal curve of dioxanopeptin A against *S. aureus* ATCC 29213 under the culture conditions of 0 °C (metabolic delay) and 37 °C (metabolic activation). Vancomycin (final concentration of 1× MIC) and daptomycin (final concentration of 1× MIC) were used as positive controls. (b) Metabolic activity and growth dynamics of *S. aureus* ATCC 29213 under the treatment of dioxanopeptin A with 0.25×, 0.5×, 1×, and 2× MICs. The inserted panel is the growth curve of *S. aureus* ATCC 29213 under the treatment of dioxanopeptin A. (c) The membrane permeability was changed by the addition of dioxanopeptin A or nisin. Compounds were added after cultivation for 15 min. (d) The change in the cytoplasmic pH in *S. aureus* treated with different concentrations of dioxanopeptin A (0.5×, 1×, 2×, and 4× MICs). (e) Membrane potential was elevated by the addition of dioxanopeptin A (0.5×, 1×, 2×, and 4× MICs). (f) Increase in the antibacterial activity of dioxanopeptin A in acid-modified media. (g) The fluidity of the membrane was still after the addition of dioxanopeptin A at the final concentrations of 0.5×, 1×, 2×, and 4× MICs. (h) Intra- and extracellular ATP levels in *S. aureus* after being treated with different concentrations of dioxanopeptin A for 30 min. Different letters within each plot indicate significant differences in grouping (*p* < 0.05; *n* = 3). Statistically significant differences were determined between groups using one-way ANOVA with Tukey's *post hoc* tests. (i) Dioxanopeptin A does not induce the accumulation of intracellular ROS in *S. aureus*. NC represents the negative control without the addition of antibiotics.

To decipher the underlying mechanism of dioxanopeptins, we first evaluated the time-killing dynamics of dioxanopeptin A (1) against *S. aureus* at metabolically static and active states. The compound showed no bactericidal activity against *S. aureus* under 0 °C conditions, but was activated when the cultivation temperature was increased to 37 °C, implying that the bactericidal pattern of dioxanopeptin is highly related to bacterial metabolism, which is similar to the mode of action of vancomycin rather than daptomycin^35^ (Fig. 6a). Furthermore, we determined the metabolic activity of *S. aureus* with the indicator dye resazurin.^36^ Resazurin can be transformed by NADH to the fluorescent product resorufin in metabolically active cells and can therefore serve as an indicator of the cellular redox state, as the reaction depends on the intracellular NAD^+^/NADH level. Our results demonstrated that dioxanopeptin at sublethal concentrations did not significantly alter the overall growth phase of *S. aureus*. However, a reduced growth rate was observed during the late logarithmic growth phase, accompanied by increased metabolic activity (Fig. 6b). Besides, the exogenous addition of dioxanopeptin did not impair the membrane permeability even when treated with the concentration of 4× MIC against *S. aureus* for 2 h (Fig. 6c). The fluidity of membrane and the accumulation of ROS were not affected in the presence of dioxanopeptin (Fig. 6d and e). Hence, we evaluated the other aspects that might be responsible for antibacterial activity of dioxanopeptin. Energy generation is necessary for bacterial survival when faced with antibiotic challenge.^34^ We found that ATP production was decreased in a dose-dependent manner after being treated with dioxanopeptin (Fig. 6f). PMF is vital for ATP production and is modulated by a delicate compensation mechanism, in which dissipation of either the cytoplasmic pH gradient (ΔpH) or membrane potential (Δ*ψ*) is compensated by a counteractive increment in the other.^37^ To understand the role of dioxanopeptin in PMF change, we measured the Δ*ψ* and ΔpH using fluorescent dyes DiSC_3_(5) and BCECF-AM, respectively. The fluorescent dye DiSC_3_(5) exhibits increased fluorescence intensity with a higher membrane potential, while BCECF-AM shows enhanced fluorescence as the intracellular environment becomes more basic. The results showed that the ΔpH significantly decreased to counteract the up-regulation of Δ*ψ* in the presence of dioxanopeptin (Fig. 6g and h), and both components were changed in a dose-dependent manner, indicating that dioxanopeptin disrupted the homeostasis of PMF, leading to the reduction of ATP production. Correspondingly, we found that dioxanopeptin displayed enhanced antibacterial activity under acidic conditions, consistent with the ability of dioxanopeptin to lower the cytoplasmic pH (Fig. 6g and i).

To examine whether the pyruvyl-like moiety is responsible for the antibacterial activity, we compared the bioactivity of dedioxanopeptin B (8) to dioxanopeptin B (2). Compound 8 displayed markedly reduced activity relative to 2, with MICs increasing from 8–16 µg mL^−1^ to >64 µg mL^−1^ (Table 1). We propose that the pyruvyl-like carboxylate in an amphiphilic scaffold of dioxanopeptins may facilitate interfacial proton exchange at the membrane–water interface, thereby biasing PMF disruption toward ΔpH dissipation. A decrease in ΔpH can be accompanied by a compensatory increase in Δ*ψ*. However, the overall PMF remains perturbed, consistent with the observed ATP decrease. Collectively, these data indicate that the pyruvyl-like moiety is required for antibacterial potency of dioxanopeptins and are consistent with a model in which dioxanopeptins primarily collapse ΔpH, thereby perturbing PMF and lowering cellular ATP levels.

In antifungal assays against a panel of plant-pathogenic fungi, dioxanopeptins exhibited no significant inhibitory activity against *Fusarium* spp., *Rhizoctonia solani*, or *Colletotrichum fructicola* (Table S8).

## Conclusions

This study, guided by a lipid-donor-anchored genome-mining workflow using PUFAS core genes as the primary entry point and NRPS co-localization as a secondary filter, identified a previously unassigned *dxp* family and linked it to dioxanopeptins by targeted cultivation, LC-MS/MS, isotope labeling, and NMR structure elucidation. The identified dioxanopeptins (1–6) are a new family of non-ribosomal lipopeptide antibiotics from *C*. *koreensis* DSM 17726 that feature a long polyunsaturated lipid tail bearing a 1,3-dioxane motif, together with dedioxanopeptins (7–10) lacking the pyruvyl-derived ketal. Functionally, dioxanopeptins display selective antibacterial activity against MDR Gram-positive bacteria, and mechanistic assays indicate that their activity correlates with perturbation of PMF homeostasis and reduced ATP production. Importantly, a pyruvyl-free analogue (8) generated from 2 under acidic conditions shows markedly diminished potency, indicating that the pyruvyl-derived functionality is required for antibacterial activity.

Biosynthetically, the presence of the 1,3-dioxane motif suggested a pyruvyl ketal installed onto a diol-bearing lipid chain. This hypothesis was evidenced by *in vitro* assays, where recombinant Ck-DxpH catalyzed the conversion of dedioxanopeptin B (8) to dioxanopeptin B (2) using potassium phosphoenolpyruvate as the pyruvyl donor. This indicates pyruvylation as a post-assembly tailoring step.

Methodologically, our genome mining outcome also highlights the limitation of lipid-donor markers. *pfa*-type PUFAS modules are not exclusive to NRPS lipopeptides, as reflected by the recovery of the *zmn* and *fcl* families in our dataset. At the same time, this rediscovery serves as an internal positive control that the workflow retrieves established PUFAS-associated pathways while still enabling prioritization of previously untapped families such as *dxp*. Collectively, these results indicate that lipid-donor-anchored mining will benefit from stricter product-class constraints, for example, by coupling lipid-donor signatures with NRPS features indicative of *N*-acylation (*e.g.*, fatty acyl-AMP ligase/ACP or starter C domains) and with clear termination logic (*e.g.*, terminal thioesterase or reductase domains), to prioritize BGCs most likely encoding NRPS lipopeptides.

## Author contributions

Y.-M. S. designed the project. Y. C. performed compound isolation, structural elucidation, and chemical conversion under the supervision of Y. X. Y. S. C. and J. D. conducted antibacterial and antifungal assays. H. X. performed biosynthetic gene cluster analysis. C. L. performed protein purification and enzyme *in vitro* assays together with Y. C. K. H. conducted computational calculations of carbon chemical shifts. X. Z. performed NMR data acquisition. J. L. conducted strain metabolic analysis. All authors contributed to manuscript writing.

## Conflicts of interest

There are no conflicts to declare.

## Supplementary Material

SC-017-D6SC00003G-s001

SC-017-D6SC00003G-s002

## Data Availability

The data supporting this article have been included as part of the supplementary information (SI). Supplementary information: ^1^H and ^13^C NMR spectra, HPLC-HRMS data, and further experimental details. See DOI: https://doi.org/10.1039/d6sc00003g. The code for mining dioxanopeptins can be found at GitHub https://github.com/SIAT-SyM-Group/2025-dxpBGC-mining.

## References

[cit1] Zhang S., Chen Y., Zhu J., Lu Q., Cryle M. J., Zhang Y., Yan F. (2023). Nat. Prod. Rep..

[cit2] Hachmann A.-B., Sevim E., Gaballa A., Popham D. L., Antelmann H., Helmann J. D. (2011). Antimicrob. Agents Chemother..

[cit3] Schneider T., Müller A., Miess H., Gross H. (2014). Int. J. Med. Microbiol..

[cit4] Butler M. S., Capon R. J. (2026). Nat. Prod. Rep..

[cit5] Zhang Z., Zhou Y., Xie S., Liu R. Z., Huang Z., Saravana Kumar P., Feng G., Yuan F., Zhang L. (2025). J. Am. Chem. Soc..

[cit6] Wang Z., Koirala B., Hernandez Y., Zimmerman M., Park S., Perlin D. S., Brady S. F. (2022). Nature.

[cit7] Dose B., Niehs S. P., Scherlach K., Flórez L. V., Kaltenpoth M., Hertweck C. (2018). ACS Chem. Biol..

[cit8] Gotze S., Vij R., Burow K., Thome N., Urbat L., Schlosser N., Pflanze S., Muller R., Hansch V. G., Schlabach K., Fazlikhani L., Walther G., Dahse H. M., Regestein L., Brunke S., Hube B., Hertweck C., Franken P., Stallforth P. (2023). J. Am. Chem. Soc..

[cit9] Ali N., Pang Z., Wang F., Xu B., El-Seedi H. R., Hussain A. (2022). J. Food Qual..

[cit10] Liong A., Leao P. N. (2025). Nat. Prod. Rep..

[cit11] Süssmuth R. D., Mainz A. (2017). Angew Chem. Int. Ed. Engl..

[cit12] Shulse C. N., Allen E. E. (2011). PLoS One.

[cit13] Hayashi S., Satoh Y., Ujihara T., Takata Y., Dairi T. (2016). Sci. Rep..

[cit14] Navarro-Muñoz J. C., Selem-Mojica N., Mullowney M. W., Kautsar S. A., Tryon J. H., Parkinson E. I., De Los Santos E. L. C., Yeong M., Cruz-Morales P., Abubucker S., Roeters A., Lokhorst W., Fernandez-Guerra A., Cappelini L. T. D., Goering A. W., Thomson R. J., Metcalf W. W., Kelleher N. L., Barona-Gomez F., Medema M. H. (2019). Nat. Chem. Biol..

[cit15] Draisma A., Loureiro C., Louwen N. L. L., Kautsar S. A., Navarro-Muñoz J. C., Doering D. T., Mouncey N. J., Medema M. H. (2026). Nat. Commun..

[cit16] Zdouc M. M., Blin K., Louwen N. L. L., Navarro J., Loureiro C., Bader C. D., Bailey C. B., Barra L., Booth T. J., Bozhuyuk K. A. J., Cediel-Becerra J. D. D., Charlop-Powers Z., Chevrette M. G., Chooi Y. H., D’Agostino P. M., de Rond T., Del Pup E., Duncan K. R., Gu W., Hanif N., Helfrich E. J. N., Jenner M., Katsuyama Y., Korenskaia A., Krug D., Libis V., Lund G. A., Mantri S., Morgan K. D., Owen C., Phan C. S., Philmus B., Reitz Z. L., Robinson S. L., Singh K. S., Teufel R., Tong Y., Tugizimana F., Ulanova D., Winter J. M., Aguilar C., Akiyama D. Y., Al-Salihi S. A. A., Alanjary M., Alberti F., Aleti G., Alharthi S. A., Rojo M. Y. A., Arishi A. A., Augustijn H. E., Avalon N. E., Avelar-Rivas J. A., Axt K. K., Barbieri H. B., Barbosa J. C. J., Barboza Segato L. G., Barrett S. E., Baunach M., Beemelmanns C., Beqaj D., Berger T., Bernaldo-Aguero J., Bettenbuhl S. M., Bielinski V. A., Biermann F., Borges R. M., Borriss R., Breitenbach M., Bretscher K. M., Brigham M. W., Buedenbender L., Bulcock B. W., Cano-Prieto C., Capela J., Carrion V. J., Carter R. S., Castelo-Branco R., Castro-Falcon G., Chagas F. O., Charria-Giron E., Chaudhri A. A., Chaudhry V., Choi H., Choi Y., Choupannejad R., Chromy J., Donahey M. S. C., Collemare J., Connolly J. A., Creamer K. E., Crusemann M., Cruz A. A., Cumsille A., Dallery J. F., Damas-Ramos L. C., Damiani T., de Kruijff M., Martin B. D., Sala G. D., Dillen J., Doering D. T., Dommaraju S. R., Durusu S., Egbert S., Ellerhorst M., Faussurier B., Fetter A., Feuermann M., Fewer D. P., Foldi J., Frediansyah A., Garza E. A., Gavriilidou A., Gentile A., Gerke J., Gerstmans H., Gomez-Escribano J. P., Gonzalez-Salazar L. A., Grayson N. E., Greco C., Gomez J. E. G., Guerra S., Flores S. G., Gurevich A., Gutierrez-Garcia K., Hart L., Haslinger K., He B., Hebra T., Hemmann J. L., Hindra H., Hoing L., Holland D. C., Holme J. E., Horch T., Hrab P., Hu J., Huynh T. H., Hwang J. Y., Iacovelli R., Iftime D., Iorio M., Jayachandran S., Jeong E., Jing J., Jung J. J., Kakumu Y., Kalkreuter E., Kang K. B., Kang S., Kim W., Kim G. J., Kim H., Kim H. U., Klapper M., Koetsier R. A., Kollten C., Kovacs A. T., Kriukova Y., Kubach N., Kunjapur A. M., Kushnareva A. K., Kust A., Lamber J., Larralde M., Larsen N. J., Launay A. P., Le N. T., Lebeer S., Lee B. T., Lee K., Lev K. L., Li S. M., Li Y. X., Licona-Cassani C., Lien A., Liu J., Lopez J. A. V., Machushynets N. V., Macias M. I., Mahmud T., Maleckis M., Martinez-Martinez A. M., Mast Y., Maximo M. F., McBride C. M., McLellan R. M., Bhatt K. M., Melkonian C., Merrild A., Metsa-Ketela M., Mitchell D. A., Muller A. V., Nguyen G. S., Nguyen H. T., Niedermeyer T. H. J., O’Hare J. H., Ossowicki A., Ostash B. O., Otani H., Padva L., Paliyal S., Pan X., Panghal M., Parade D. S., Park J., Parra J., Rubio M. P., Pham H. T., Pidot S. J., Piel J., Pourmohsenin B., Rakhmanov M., Ramesh S., Rasmussen M. H., Rego A., Reher R., Rice A. J., Rigolet A., Romero-Otero A., Rosas-Becerra L. R., Rosiles P. Y., Rutz A., Ryu B., Sahadeo L. A., Saldanha M., Salvi L., Sanchez-Carvajal E., Santos-Medellin C., Sbaraini N., Schoellhorn S. M., Schumm C., Sehnal L., Selem N., Shah A. D., Shishido T. K., Sieber S., Silviani V., Singh G., Singh H., Sokolova N., Sonnenschein E. C., Sosio M., Sowa S. T., Steffen K., Stegmann E., Streiff A. B., Struder A., Surup F., Svenningsen T., Sweeney D., Szenei J., Tagirdzhanov A., Tan B., Tarnowski M. J., Terlouw B. R., Rey T., Thome N. U., Torres Ortega L. R., Torring T., Trindade M., Truman A. W., Tvilum M., Udwary D. W., Ulbricht C., Vader L., van Wezel G. P., Walmsley M., Warnasinghe R., Weddeling H. G., Weir A. N. M., Williams K., Williams S. E., Witte T. E., Rocca S. M. W., Yamada K., Yang D., Yang D., Yu J., Zhou Z., Ziemert N., Zimmer L., Zimmermann A., Zimmermann C., van der Hooft J. J. J., Linington R. G., Weber T., Medema M. H. (2024). Nucleic Acids Res..

[cit17] Fuchs S. W., Grundmann F., Kurz M., Kaiser M., Bode H. B. (2014). ChemBioChem.

[cit18] Masschelein J., Clauwers C., Awodi U. R., Stalmans K., Vermaelen W., Lescrinier E., Aertsen A., Michiels C., Challis G. L., Lavigne R. (2015). Chem. Sci..

[cit19] Paulo B. S., Recchia M. J. J., Lee S., Fergusson C. H., Romanowski S. B., Hernandez A., Krull N., Liu D. Y., Cavanagh H., Bos A., Gray C. A., Murphy B. T., Linington R. G., Eustaquio A. S. (2024). Chem. Sci..

[cit20] Shi Y.-M., Bode H. B. (2018). Nat. Prod. Rep..

[cit21] Masschelein J., Jenner M., Challis G. L. (2017). Nat. Prod. Rep..

[cit22] Shi Y.-M., Hirschmann M., Shi Y.-N., Ahmed S., Abebew D., Tobias N. J., Grün P., Crames J. J., Pöschel L., Kuttenlochner W., Richter C., Herrmann J., Müller R., Thanwisai A., Pidot S. J., Stinear T. P., Groll M., Kim Y., Bode H. B. (2022). Nat. Chem..

[cit23] Zhang Z., Zhou Y., Xie S., Liu R.-Z., Huang Z., Saravana Kumar P., Feng G., Yuan F., Zhang L. (2025). J. Am. Chem. Soc..

[cit24] Terlouw B. R. , Huang C., Meijer D., Cediel-Becerra J. D. D., Rothe M. L., Jenner M., Zhou S., Zhang Y., Fage C. D., Tsunematsu Y., van Wezel G. P., Robinson S. L., Alberti F., Alkhalaf L. M., Chevrette M. G., Challis G. L. and Medema M. H., bioRxiv, 2025, preprint, 10.1101/2025.01.08.631717

[cit25] Bus J., Sies I., Lie Ken Jie M. S. F. (1976). Chem. Phys. Lipids.

[cit26] Alexandri E., Ahmed R., Siddiqui H., Choudhary M. I., Tsiafoulis C. G., Gerothanassis I. P. (2017). Molecules.

[cit27] Xiang C., Yao S., Wang R., Zhang L. (2024). Beilstein J. Org. Chem..

[cit28] Sinnaeve D., Foroozandeh M., Nilsson M., Morris G. A. (2016). Angew Chem. Int. Ed. Engl..

[cit29] Ermanis K., Parkes K. E., Agback T., Goodman J. M. (2016). Org. Biomol. Chem..

[cit30] Grimblat N., Zanardi M. M., Sarotti A. M. (2015). J. Org. Chem..

[cit31] Hayashi S., Satoh Y., Ogasawara Y., Maruyama C., Hamano Y., Ujihara T., Dairi T. (2019). Angew Chem. Int. Ed. Engl..

[cit32] Labonte J. W., Townsend C. A. (2013). Chem. Rev..

[cit33] Miller W. R., Arias C. A. (2024). Nat. Rev. Microbiol..

[cit34] Lobritz M. A., Belenky P., Porter C. B., Gutierrez A., Yang J. H., Schwarz E. G., Dwyer D. J., Khalil A. S., Collins J. J. (2015). Proc. Natl. Acad. Sci. U. S. A..

[cit35] Buttress J. A., Schafer A. B., Koh A., Wheatley J., Mickiewicz K., Wenzel M., Strahl H. (2025). Nat. Commun..

[cit36] Silva F. S. G., Starostina I. G., Ivanova V. V., Rizvanov A. A., Oliveira P. J., Pereira S. P. (2016). Curr. Protoc. Toxicol..

[cit37] Bakker E. P., Mangerich W. E. (1981). J. Bacteriol..

