# Supplementary material for: Lipid-donor-anchored genome mining uncovers dioxanopeptins, antibacterial lipopeptides with a 1,3-dioxane functionalized polyunsaturated lipid tail
Source: Chem Sci. 2026 Feb 27;17(16):8229–41. doi: 10.1039/d6sc00003g (PMC12964715; doi:10.1039/d6sc00003g)
Supplement: SC-017-D6SC00003G-s001 [file SC-017-D6SC00003G-s001.pdf]

Supplementary Information (SI) for Chemical Science.
This journal is © The Royal Society of Chemistry 2026

Supplementary Information

Lipid-donor-anchored genome mining uncovers dioxanopeptins, antibacterial lipopeptides with a 1,3-dioxane functionalized polyunsaturated lipid tail

Ying Chen[1,2#], Yunsheng Chen[2#], Hao Xiang[2,6#], Changqi Luo,[2] Jiaqi Duan[2], Kun Hu[3,6], Xiaohong Zheng[4], Jing Liu[5], Yongbo Xue[1*], Yi-Ming Shi[2,6*]

[1]School of Pharmaceutical Sciences (Shenzhen), Sun Yat-sen University, Shenzhen, China

[2]State Key Laboratory of Quantitative Synthetic Biology, Center for Synthetic Biochemistry, Shenzhen Institute of Synthetic Biology, Shenzhen Institutes of Advanced Technology, Chinese Academy of Sciences, Shenzhen, China

[3]State Key Laboratory of Phytochemistry and Natural Medicines, Kunming Institute of Botany, Chinese Academy of Sciences, Kunming, China

[4]Equipment Public Service Center, South China Sea Institute of Oceanology, Guangzhou, China

[5]Department of Natural Products in Organismic Interactions, Max Planck Institute for Terrestrial Microbiology, Marburg, Germany

[6]University of Chinese Academy of Sciences, Beijing, China

#These authors contributed equally to the work.

*Corresponding author

Materials and Methods

1. General Experimental Procedures. Isotope-labelled chemicals were purchased from Cambridge Isotope Laboratories. HPLC-HRMS analysis was conducted on an Impact II qTof mass spectrometer (Bruker) with a RRHD Eclipse XDB-C18 column (80 Å, 2.1 mm × 50 mm, 1.8-μm particle size, Agilent) at a flow rate of 0.3 mL/min (5–95% acetonitrile/water with 0.1% formic acid, vol/vol, 12 min). Flash purification was performed on a SepaBean machine T flash purification system by a silica gel column chromatography (Santai E-8101-0220, 220 g) with a self-packed pre-column (Santai, Puriflash dry-load empty E-8101-0220 Flash column) coupled with a UV detector HPLC purification was performed on preparative and semipreparative Agilent 1260 systems coupled to a diode array detector (DAD) with a C18 ZORBAX Eclipse XDB column (9.4 mm × 250 mm, 5 μm, 3 mL/min; 4.6 mm × 250 mm, 5 μm, 1 mL/min). Freeze drying was performed using equipment of SCIENTZ-10N/C. NMR experiments were carried out on a Bruker AVANCE III HD 700 MHz spectrometer equipped with a 5-mm cryoprobe.

2. antiSMASH Annotations and Genome Mining. Representative Shewanella-type PfaA (type A, AAN54658.1) and Aureispira-type PfaA (type D, BAP47694.1) proteins were used as query sequences[1, 2], and two independent BLASTP[3] searches were performed against the NCBI RefSeq protein database. Each BLASTP search returned up to 5,000 hits; therefore, merging the two searches yielded a total of 10,000 BLASTP hits. For de-duplication, hits were collapsed by the NCBI taxonomic identifier (taxon ID), retaining a single representative per taxon ID. For each BLASTP hit, the corresponding genomic neighborhood spanning 100 kb (±50 kb) was retrieved, resulting in 3,088 genomic contexts, and annotated with antiSMASH v8.0[4] to predict BGCs. In total, 3,080 BGC-containing GenBank outputs were obtained and compiled into a local BGC database.

To enrich for loci encoding *pfa* multi-gene cassettes, we then queried the local BGC database with cblaster[5], using Shewanella-type *pfaACD* and Aureispira-type *pfaA–D*[1, 2]as independent query sets, and retained hits meeting ≥20% amino-acid identity and ≥30% query coverage. We further applied a streamlined rule-based filter to obtain PFUA-NRPS hybrid candidates, requiring (i) PKS_AT (PF00698.24) and ketoacyl synthase (KS, PF00109.29, PF02801.25, and PF16197.8) signatures, (ii) co-localization of NRPS and PUFA KS within 25 kb based on antiSMASH annotated core coordinates, and (iii) enrichment of hallmark catalytic domains consistent with the targeted architecture (adenylation domain, PF00501.31; condensation domain, PF00668.23). Applying this workflow, we identified 60 candidate PUFAS–NRPS BGCs. To assess relatedness to previously characterized gene clusters, we clustered these 60 candidates together with MIBiG v4.0[6] BGCs using BiG-SCAPE v2.0.1[7, 8], running bigscape cluster --classify legacy --legacy-weights --hybrids-off --mix --include-singletons --gcf-cutoffs 0.20, 0.30, 0.40, 0.50, 0.60, 0.70, 0.80. This clustering revealed a distinct *dxp* family that did not co-cluster with any MIBiG entries. The 60 candidate PUFAS-NRPS BGCs were then carried forward for downstream phylogenetic, classification, and synteny analyses to delineate the *dxp* family from other co-occurring families (e.g., *fcl*, *zmn,* and *mgp*).

To enable comparative analyses across clusters, we defined a conserved core-gene set to classify the 60 BGCs, including PUFAS components (*pfaA*, *pfaC*, and *pfaD*) and the co-localized NRPS component. For each core gene, BLASTP searches were performed against a protein database derived from the 60 BGCs, retaining hits with ≥20% amino-acid identity and ≥30% query coverage, and keeping a single best-scoring hit per BGC. Homolog sets were aligned with MUSCLE v5.3[9] and trimmed with ClipKIT[10] (v2.7.0 mode kpic-smart-gap). For each trimmed multiple sequence alignment (MSA), substitution-model selection was performed with ModelTest-NG[11] (v0.1.7 amino-acid mode), and the

best-fitting model was LG+I+G4+F for all four core genes. The four trimmed alignments were then concatenated into a core-gene MSA, which was used for phylogenetic inference with RAxML-NG[11, 12] v1.2.2 under LG+I+G4+F, using 100 random starting trees and 1,000 bootstrap replicates to assess node support. Gene-cluster synteny across the 60 BGCs was evaluated by linear comparison with clinker[5] (Supplementary Figure 1). Based on the combined evidence from the core-gene phylogeny and conserved synteny, the 60 BGCs segregated into three major clades. The distinct clade highlighted in Figure 3b was designated as the *dxp* family. Custom BGC mining workflow is available via Google Colab at: https://colab.research.google.com/github/SIAT-SyM-Group/2025-dxpBGC-mining/blob/main/workflow.ipynb.

3. Comparison of KR$_C$ Subdomains. The catalytic subdomain of KR (KR$_C$)sequences were curated from a published, updated motif-based framework for stereoselectivity prediction[13] and combined with the KR$_C$ segments extracted from Ck-DxpD and Ck-DxpB. Amino acid sequences were aligned with Clustal Omega[14] under default parameters. Substitution-model selection was performed with ModelTest-NG[11], yielding WAG+G4+F[15]. Maximum-likelihood phylogenies were inferred with RAxML-NG[12], and node support was assessed with 1,000 bootstrap replicates[16]. Trees were visualized and annotated in iTOL[17], and rendered as both a global overview and a local, zoomed-in view to resolve the placement of the query sequences (Supplementary Figure 3).

4. A-domain Substrate Specificity Prediction. To profile NRPS A domain substrate selectivity[18], we applied three complementary predictors in parallel: NRPSTransformer[19] and PARAS/PARASECT[20]. NRPStransformer was run in Substrate Prediction mode, returning a ranked list of candidate substrates with confidence scores (43-class output), from which the top three predictions were extracted. PARAS was executed in the PARAS (all substrates) setting, which predicts A-domain specificity against a predefined panel of

223 substrates and reports ranked candidates with confidence scores. PARASECT was run in the PARASECT (bacterial) setting (trained on bacterial data only) and used to score whether predefined and/or user-supplied substrates are predicted to interact with each A domain; substrates were ranked by the predicted interaction score. Unless stated otherwise, default parameters were used for all tools. For each method, the top three candidates and their associated scores were recorded and reported (Supplementary Table 1). Because scoring schemes are method-specific, scores were used only for within-tool ranking and were not interpreted as directly comparable across tools.

5. Culture Conditions, Extraction, and Detection. Wild-type strains (Supplementary Table 2) were cultivated on LB agar plates at 30 ^{o}C overnight and were subsequently inoculated into liquid LB culture at 30 ^{o}C with shaking at 220 rpm. For compound production, the overnight LB culture of a mutant was transferred into 5 mL LB/PP3/XPP/830/CYE media (1:100, v/v) with 2% (v/v) of Amberlite^{TM} XAD-16 resins at 30 ^{o}C with shaking at 220 rpm. The XAD-16 resins were collected after 72 h and extracted with 5 mL of methanol. The solvent was dried under rotary evaporators, and the dried extract was resuspended in 500 µL methanol, of which 5 µL was injected and analyzed by HPLC-HRMS. Unless otherwise specified, HPLC-HRMS chromatograms in the figures are shown on the same scale.

6. Labelling Experiments for Structural Elucidation by MS. The cultivation of strains for labelling experiments was carried out as described above. The cell pellets of the 100 µL overnight culture were washed twice with dd H_2O and then resuspended in dd H_2O (100 µL). A 5 mL of the ^{13}C or ^{15}N isotope labelling medium was inoculated with a washed overnight culture (100 µL).

7. Isolation and Purification. The fermentation was initiated by transferring 10 mL of the seed culture to 1 L of PP3 medium in a 5 L flask supplemented with 2% (v/v) XAD resin,

followed by incubation for 3 days at 30°C and 220 rpm in a shaking incubator. In total, 23.5 L PP3 culture of *Chitinimonas koreensis* DSM 17726 was collected after 72 h of incubation at 30°C with shaking at 220 rpm, and then washed with water and extracted with methanol (3 × 0.6 L) to yield a crude extract (4.5 g after solvent removal). The extract was then fractionated by silica gel column chromatography (230−400 mesh) using a stepwise gradient of CH_2Cl_2 and MeOH to yield 9 fractions (Fractions1−9). Fractions 4−6 (0.95 g) containing dioxanopeptins, were further purified by semipreparative HPLC on a C18 column using an acetonitrile/water gradient (0.1% formic acid; 20–65% over 55 min; 3 mL/min) to afford dioxanopeptins A (12.0 mg), B (28.1 mg), C (3.1 mg) and D (1.5 mg).

8. NMR Spectroscopy. Measurements were carried out using ^{1}H and ^{13}C NMR, ^{1}H-^{13}C heteronuclear single quantum coherence (HSQC), ^{1}H-^{13}C heteronuclear multiple bond correlation (HMBC), ^{1}H-^{1}H correlation spectroscopy (COSY), ^{1}H-^{13}C heteronuclear multiple quantum correlation/^{1}H-^{1}H correlation spectroscopy (HMQC-COSY), and ^{1}H-^{13}C heteronuclear single quantum coherence/^{1}H-^{1}H total correlation spectroscopy (HSQC-TOCSY). Chemical shifts (*δ*) were reported in parts per million (ppm) and referenced to the solvent signals. Data are reported as follows: chemical shift, multiplicity (br = broad, s = singlet, d = doublet, t = triplet, dd = doublet of doublet, m = multiplet, and ov = overlapped), and coupling constants (in hertz). Bruker TopSpin 4.0 was used for NMR data collection and spectral interpretation.

9. Calculation and Comparison of ^{13}C NMR Chemical Shifts.

(1) Structural Simplification. To manage computational cost and reduce conformational complexity, the long aliphatic chain (C1–C20) of compound **1** was truncated, as it is spectroscopically less diagnostic for the core structure. This generated a simplified core model, **1T**. To probe the undefined stereochemistry at C-25, two epimers were constructed from the **1T** scaffold: **1T-a** (25*R*) and **1T-b** (25*S*), while the configurations at C-28, C-34,

C-39, C-41, and C-44 were fixed as *S*, *S*, *S*, *R*, and *R*, respectively. All subsequent calculations were performed independently on **1T-a** and **1T-b**.

(2) Conformational Ensemble and Boltzmann Weighting. For each model, conformational sampling was performed with Crest (v.3.0.1)[21]. The resulting raw conformers were clustered using a maximum distance matrix difference threshold of 0.5 Å, and only the lowest-energy representative from each cluster was retained. From this unique set, conformers within a 4 kcal/mol energy window underwent geometry optimization at the B3LYP-D3BJ/6-31G(d) level in the gas phase, followed by frequency analysis to confirm true minima. More accurate single-point energies were then obtained at the M06-2X-D3/6-311+G (2d, p) level. The Gibbs free energy for each conformer was calculated by adding this electronic energy to the thermal correction from frequency analysis. A final deduplication step removed any DFT-optimized structures with an RMSD < 0.25 Å and an energy difference < 0.25 kcal/mol. The room-temperature (298.15 K) equilibrium population (p_i) for each remaining conformer was then calculated based on its relative Gibbs free energy (ΔG_i) using the Boltzmann distribution:

$$p_i = \frac{n_i}{\sum_j n_j} = \frac{e^{-\Delta G_i/RT}}{\sum_j e^{-\Delta G_j/RT}}$$

Where T is room temperature (298.15 K), R is the ideal gas constant (0.0019858995). These Boltzmann weights were subsequently used for the conformational averaging of calculated chemical shifts.

(3) NMR Chemical Shift Calculation. NMR shielding tensors for the weighted conformers were computed using the GIAO method at mPW1PW91-SCRF/6-31+G(d,p) level (dimethyl sulfoxide, IEFPCM solvent model). The shielding constants obtained were converted into chemical shifts by referencing to TMS at 0 ppm ($\delta_{cal} = \sigma_{TMS} - \sigma_{cal}$), where the σ_{TMS} was the shielding constant of TMS calculated at the same level. To enable

comparison with experimental data, the final theoretical chemical shift for each nucleus ($\delta_{cal, avg}$) was obtained as the Boltzmann-weighted average of the shifts from all conformers: $\delta_{cal, avg} = \Sigma (Pi \times \delta_{cal, i})$. The parameters *a* and *b* of the linear regression $\delta_{cal} = a\delta_{exp} + b$; the correlation coefficient, R^2; the mean absolute error (*MAE*) defined as $\Sigma n |\delta_{cal} - \delta_{exp}|/n$; the corrected mean absolute error, *CMAE*, defined as $\Sigma n |\delta_{corr} - \delta_{exp}|/n$, where $\delta_{corr} = (\delta_{cal} - b)/a$, were calculated. Then, DP4+ probability analysis[22] were undertaken using the calculated NMR chemical shifts and spreadsheet provided by Sarotti, *et al*, and DP4+ probabilities were obtained.

10. Marfey's Assays. Approximately 1 mg of peptide was dissolved in 175 µL MeOH, 25 µL triisopropylsilane was added, and the mixture was hydrolyzed with 6 M HCl (800 µL) in an ACE high-pressure tube at 110 °C for 1 h[23]. The hydrolysate was evaporated to dryness and re-dissolved in 100 µL H_2O. The acid hydrolysate solution was then placed in a 10 mL reaction vial and treated with a 1% solution of FDAA (200 µL) in acetone, followed by 1.0 M $NaHCO_3$ (20 µL). The amber reaction vials were incubated in a water bath at 40 °C for 1 h. The reactions were then cooled to room temperature, quenched with 1 M HCl (20 µL), and evaporated to dryness. The residue was dissolved in 400 µL MeOH. The FDAA-derivatized amino acids were analyzed by HPLC-UV and HPLC-HRMS.

11. Structure elucidation of dioxanopeptins by MS, NMR, bioinformatics, and chemical calculations. Dioxanopeptin A (**1**), a white powder, was determined to have the molecular formula $C_{48}H_{79}N_6O_6$ based on the HRESIMS ion at *m/z* 915.5801 $[M+H]^+$ and the isotope-feeding experiment results. A comprehensive examination of its ^1H NMR data (Supplementary Table 3) revealed the presence of seven methyl groups [δ_H 1.46 (s, 3-CH_3), 1.07 (d, *J* = 6.0 Hz, 5-CH_3), 0.79 (d, *J* = 6.5 Hz, 31-CH_3), 0.89 (d, *J* = 6.4 Hz, 32-CH_3), 1.02 (d, *J* = 6.4 Hz, 40-CH_3), 0.90 (d, *J* = 7.0 Hz, 46-CH_3), 0.88 (d, *J* = 7.0 Hz, 47-CH_3)]. Analysis of the ^{13}C NMR and HSQC spectra revealed 48 carbon signals. The

diagnostic NMR data suggested the presence of seven carbonyl groups [δ_C 173.2 (C-1), 173.0 (C-27), 172.1 (C-33), 173.6 (C-37), 171.2 (C-38), 172.0 (C-43), 170.6 (C-48)] and several olefinic carbon signals (δ_C 125.9 and 129.6-131.9). The planar structure of **1** was elucidated through analysis of ^{1}H-^{1}H COSY and HMBC spectra (Supplementary Figure 2). The ^{1}H-^{1}H COSY spectrum indicated the presence of a structural fragment comprising leucine (C28−C29−C30−C31/C32), glutamine (C34−C35−C36), alanine (supported by the 40-CH_3 signal), and valine (C44−C45−C46/C47) residues, along with a long unsaturated aliphatic chain (C5−C4−C6−C7−C8−C9−C10−C11~C25−C26). Within the unsaturated fatty chain, the chemical shifts of most methylene groups overlap. Further inspection of the ^{1}H NMR and HSQC spectra confirmed the presence of eight CH_2 groups and eight olefinic protons, with six of the olefinic protons also overlapping in chemical shift. Taken together, these findings suggest a regularly arranged unsaturated fatty chain in which adjacent double bonds are separated by two CH_2 units. The ^{13}C chemical shift of the allylic methylene carbons adjacent to the olefins cluster around δ_C 32 ppm. According to previous studies[24, 25], allylic methylenes next to trans double bonds typically resonate at 32–34 ppm, whereas those next to cis double bonds appear at 27–29 ppm. Therefore, we assigned the polyunsaturated double bonds as *trans*.

Connectivity between the peptide and lipid fragments was established through comprehensive analysis of HMBC and HSQC-TOCSY data. The key HMBC correlations (Supplementary Figure 2) from H-28 to C-33, H-35/H-39 to C-38, H-41 to C-43, H-44/H-45 to C-48, together with the key HSQC-TOCSY correlations from 25-NH to C-24/C-25/C-26, linked the amino acid residues to the unsaturated aliphatic chain.

We further employed Marfey's method to confirm that leucine and glutamine are L-configured, while valine is D-configured (Supplementary Figure 18). These experimental results are consistent with the amino acid stereochemistry predicted by the C/E domain

annotations. The configuration of the alanine residue, which was extended by a polyketide unit, was assigned to be L by C domain inference (Supplementary Table 6).

The stereochemistry of C-4, C-7, and C-41 was tentatively proposed to be 4*R*, 7*S*, and 41*R*, respectively, based on the analysis of the catalytic subdomain of KR (KR_{C}) sequences[13]. In the resulting phylogeny, the Ck-DxpD KR_{C} clustered within the B-type KR clade, whereas the Ck-DxpB KR_{C} grouped with the A-type KR clade, supporting type-specific stereoselectivity assignment (Supplementary Figure 3). In particular, to provide NMR evidence for the C-41 stereochemical assignment, we recorded a PSYCHEDELIC spectrum[26] on dioxanopeptin B (higher abundance) to resolve the congested multiplet and extract $^{3}J_{\mathrm{H39–H41}}$ = 7.5 Hz, which indicates an *anti* relationship between H-39 and H-41 and is in agreement with the C-41 assignment inferred from the KR_{C} phylogeny. ROESY correlations between H-3 and H-7 provided further evidence, supporting the absolute configuration at C-3 as 3*R*. To determine the configuration at C-25, we performed DFT-GIAO NMR calculations[27] on truncated structural models. The computed shifts for the 25*R* isomer (**1T-a**) showed better agreement with the experimental ^{13}C and ^{1}H data than those for the 25*S* isomer (**1T-b**), as reflected by higher R^2 and lower mean absolute error/corrected mean absolute error values. Therefore, DP4+ analysis[22] strongly supported the 25*R* isomer over 25*S*, giving a DP4+ probability of 100.00% (Supplementary Figure 2).

The structures of dioxanopeptins B (**2**) and C (**3**) were confirmed by comparative NMR analysis with dioxanopeptin A (**1**). Dioxanopeptins E (**5**) and F (**6**), with shorter retention time in the HPLC chromatogram, showed MS/MS fragmentation patterns identical to **2** and **1**, respectively, but had a +18 Da mass shift (Supplementary Figures 12 and 13). Therefore, **5** and **6** were assigned as corresponding hydrolytic linear congeners of **2** and **1**.

We observed a series of compounds (**7–10**) that eluted earlier in the HPLC chromatogram than dioxanopeptins A–C (**1–3**). The MS/MS spectra of **1–3** and **7–10** showed conserved fragmentation for the peptide moiety, while diagnostic fragments of the lipid portion differed by -70 Da ($C_3H_2O_2$, Supplementary Figures 14–17). This mass deficit is consistent with loss of a pyruvyl-like moiety, indicating that compounds **7–10** lack the pyruvyl-derived ketal.

12. Chemical Conversion of Dioxanopeptin B (2) to Dedioxanopeptin B (8). Compound 2 (10 µmol) was dissolved in MeCN/H_2O (7:3, v/v; 8 mL) in a glass vial. Aqueous HCl (0.5 M, 2 mL) was added, and the mixture was kept at 25 °C for 1 h, as previsous decribed[28]. The reaction was then neutralized to pH 7 with solid $NaHCO_3$, followed by extraction with EtOAc to afford a crude extract after solvent removal. Dedioxanopeptin B (**8**) was purified by HPLC to yield 1.7 mg for in vitro enzymatic reactions and antibacterial assays.

13. Ck-*dxpH* cloning. The Ck-*dxpH* gene was amplified by PCR from genomic DNA of *Chitinimonas koreensis* DSM 17726 using primers of DxpH-forward (ACTTTAATAAGGAGATATACCatgtccaggatgctcgaag) and DxpH-reverse (GTGGTGATGATGGTGATGgccataccgcgccaccagc). Primers DxpH-forward and DxpH-reverse were designed to introduce homology arms compatible with the insertion site of the linearized pCOLA_Duet-1 vector. In parallel, the pCOLA_Duet-1 backbone was PCR-amplified using primers pCOLA-Duet-F (CATCACCATCATCACCACtgaTTAACCTAGGCTGCTGCCACC) and pCOLA-Duet-R (GGTATATCTCCTTATTAAAGTTAAACAAAATTATTTCTACAGG). The Ck-*dxpH* insert and vector backbone fragments were assembled using Gibson Assembly Master Mix to afford pCOLA_Duet-1-DxpH, encoding DxpH fused to a C-terminal hexahistidine tag. The assembly mixture was directly transformed into chemically competent *E. coli* DH5α cells. Positive clones were screened by colony PCR using primers DxpH-Ve-F

(ACGCCAATCAGCAACGACTG) and DxpH-Ve-R (GATGTTGGACGAGTCGGAATCG), and a representative plasmid was isolated and verified by Sanger sequencing. The sequence-confirmed construct was then transformed into chemically competent *E. coli* Rosetta (DE3) cells for heterologous protein expression.

14. CK-DxpH Expression and Purification. *E. coli* cells harboring the Ck-*dxpH* expression plasmid were grown overnight at 37 °C in 3 mL of LB medium. A 1 mL aliquot of the overnight culture was used to inoculate 100 mL LB for protein expression. The culture was grown at 37 °C to an OD_{600} of ~0.6, chilled on ice for 30 min, and induced with isopropyl β-D-1-thiogalactopyranoside (IPTG) to a final concentration of 0.5 mM. After induction, the temperature was reduced to 16 °C and incubation continued for 20 h.

Cells were harvested by centrifugation at 4 °C (4,000 × *g*, 20 min). The cell pellet was thawed and resuspended in 30 mL lysis buffer (20 mM Tris–HCl, pH 7.5, 200 mM NaCl) and kept on ice. Cells were disrupted using a pre-cooled high-pressure homogenizer (Yonglian Bio UH-06) at 600 bar for three passes, with the sample maintained below 4 °C in an ice–water bath. The lysate was clarified by centrifugation at 4 °C (16,000 × *g*, 20 min), and 5 µL aliquots of the supernatant and pellet fractions were reserved for SDS–PAGE analysis.

The clarified supernatant (~30 mL) was incubated with Ni resin on ice for 20 min and then loaded onto a pre-equilibrated Ni–NTA column (Lablead N30210; 20 mL bed volume). The column was washed with 20 mL wash buffer (20 mM Tris–HCl, pH 7.5, 200 mM NaCl, 10 mM imidazole), and the bound protein was eluted with 15 mL elution buffer (20 mM Tris–HCl, pH 7.5, 200 mM NaCl, 150 mM imidazole). The eluate was collected in full and buffer-exchanged into lysis buffer using a 30 kDa MWCO ultrafiltration device.

For SDS–PAGE, 5 µL of the protein solution was mixed with loading buffer (Yeasen 20315ES), heated at 99 °C for 10 min, and resolved on a 12% precast gel (Sangon BBI

C691302-0001) at 150 V. The gel was stained with Coomassie Brilliant Blue R-250 (Biodragon BF06152) with gentle agitation at room temperature for 1 h, then destained with ddH_2O until the background was clear and protein bands were visible.

16. In Vitro Assay of Ck-DxpH. An in vitro reaction was prepared containing dedioxanopeptin B (**8**, 50 μM), potassium phosphoenolpyruvate (10 mM), Bicine (25 mM), $MgCl_2$ (10 mM), DMSO (10%, v/v), and purified Ck-DxpH (20 μM), and incubated at 25 °C for 1 h[29]. The reaction was quenched by the addition of an equal volume of acetonitrile, and the resulting mixture was subjected to LC–HRMS analysis.

17. Antibacterial Assays.

(1) Antibacterial Tests. The MICs of dioxanopeptins and other antibiotics were determined by the broth microdilution method according to the CLSI 2021 guideline. Briefly, dioxanopeptins or other antibiotics were diluted in cation adjusted Mueller Hinton Broth (CAMHB) and mixed with an equal volume of the bacterial supernatant in CAMHB containing approximately 10^6 colony-forming units (CFUs)/mL in a 96-well microplate. The lowest concentrations of antibiotics with no visible growth of bacteria were the MICs and the cells in MIC or higher than it were performed colony count with no visible colony of bacteria were the MBCs. Both were determined after incubation at 37 °C for 16 to 18 h. The pH of the medium was adjusted to 5–9 by HCl or NaOH if needed.

(2) Growth Curve Determination. Strains were cultured in 1 mL of LB broth for approximately 16 h at 37 °C with shaking at 200 rpm. The overnight cultures were diluted in Mueller-Hinton Broth (MHB) mixed with 25 μg/mL resazurin and adjusted to approximately 10^6 CFUs/mL. Different concentrations of dioxanopeptin or other antibiotics were added to a 96-well microplate and mixed with an equal volume of bacterial dilutions. Growth curves were established under absorption spectroscopy at the wavelength of OD_{500} nm and fluorescence spectroscopy using an excitation wavelength at 550 nm and

an emission wavelength at 590 nm with an interval of 10 min at 37 °C for 24 h, by SpectraMax iD3 multi-mode microplate reader (Molecular Devices)

(3) Time-dependent Killing Assay. *S. aureus* ATCC 29213 grown to the exponential phase in MHB at 37°C with 200 rpm shaking was diluted to a final concentration of 10^7 CFU/mL, and briefly modified as previously described[30]. Different concentrations of dioxanopeptin or other antibiotics were added and cultured at 0°C or 37°C with shaking at 220 rpm. The bacterial suspension was precooled in an ice bath before measurement of bactericidal kinetics at 0°C. 500 μL aliquots were removed and serially diluted to plate on LB plates after culturing for 2, 4, 6, 8, and 10 h to calculate the CFUs after incubation at 37°C for 24 h.

(4) Membrane Integrity Assay. *S. aureus* ATCC 29213 was centrifuged and resuspended in 0.01 mol/L PBS (pH 7.4) to adjust bacterial suspensions to an OD_{600} nm of 0.5, followed by the addition of propidium iodide (PI) at a final concentration of 10 μM. The bacterial suspensions were incubated at 37°C for 20 min without light and then centrifuged and removed the supernatant, then challenged by dioxanopeptin at 0.5×, 1×, 2×, and 4× MICs. Nisin was used as a positive control at a final concentration of 100 μg/mL. The fluorescence was measured under the excitation wavelength at 535 nm and emission wavelength at 615 nm.

(5) ∆pH Measurement. Overnight culture of *S. aureus* ATCC 29213 was washed three times with HEPES (5 mM, pH 7.0, plus 5 mM glucose) and resuspended to obtain an OD_{600} nm of 0.5. The cytoplasmic pH was determined by the pH-sensitive fluorescence probe BCECF-AM[31] at a final concentration of 10 μM and incubated at 37°C for 20 min. The fluorescence value was determined per 5 min for 2 h, with the excitation wavelength at 488 nm and emission wavelength at 535 nm. Carbonyl cyanide 3-chlorophenylhydrazone (CCCP) was used as a positive control at a final concentration of

10 μM. Then, dioxanopeptin (0.5×, 1×, 2×, and 4× MICs) was added and the fluorescence value was continuously monitored.

(6) Membrane Depolarization Assay. *S. aureus* ATCC 29213 suspension was prepared as ΔpH measurement method described. 3,3-dipropylthiadicarbocyanine iodide $DiSC_3(5)$ at a final concentration of 5 μM was added and incubated at 37°C for 20 min[32]. The membrane potential of *S. aureus* ATCC 29213 in the presence of dioxanopeptin or CCCP (at a final concentration of 10 μM) was measured using an excitation wavelength at 622 nm and an emission wavelength at 670 nm.

(7) Membrane Fluidity Assays. Overnight culture of *S. aureus* ATCC 29213 grown to the exponential phase for 6–8 h at 37 °C was collected and resuspended with 0.01 mol/L PBS (pH 7.4) to adjust bacterial suspensions to approximately an OD600 nm of 0.5. Then, *S. aureus* was stained by a final concentration of 10 μmol/L laurdan, and incubated at 37 °C for 30 min. Subsequently, bacterial suspensions were treated with 0.5×, 1×, 2×, and 4× MICs concentrations of dioxanopeptin or tetracycline (50 μg/mL). The fluorescence intensity was monitored for 2 h per 5 min with excitation wavelength at 350 nm and emission wavelength at 438 nm. Laurdan GP was calculated according to toprevious studies[33].

(8) ROS Measurement. *S. aureus* ATCC 29213 cultured overnight was washed three times with 0.01 mol/L PBS (pH 7.4) and resuspended to reach an OD_{600} nm of 0.5. 2',7'-dichlorodihydrofluorescein diacetate (DCFH-DA) was added at a final concentration of 10 μM) to the bacterial suspension and incubated at 37°C for 30 min. After washing with PBS three times, 190 μL bacterial suspension was added to a 96-well plate and mixed with 10 μL dioxanopeptin at 0.5×, 1×, 2×, and 4× MICs. Ciprofloxacin (CIP) was used as a positive control at a final concentration of 50 μg/mL. The fluorescence intensity was monitored for

2 h per 5 min by SpectraMax iD3 multi-mode microplate reader (Molecular Devices), with the excitation wavelength at 488 nm and emission wavelength at 528 nm.

(9) ATP Determination. Intracellular and extracellular ATP levels of *S. aureus* ATCC 29213 were determined using an Enhanced ATP Assay Kit as previously described[34]. *S. aureus* ATCC 29213 suspension was prepared as ROS measurement method described above. Then, the bacterial suspensions were treated with dioxanopeptin (at final concentrations of 0.5×, 1×, 2×, and 4× MICs) for 30 min. The bacterial supernatant was collected by centrifugation at 12,000 rpm at 4°C for 5 min and used for measuring extracellular ATP levels. The bacterial precipitates were collected and lysed by lysostaphin for 20 min at 37°C, then resuspended at the equal volume of the supernatant used for measuring intracellular ATP levels. The detecting solution was added to a 96-well plate and incubated at room temperature for 5 min. Subsequently, the supernatants were added to the well and mixed quickly before recording in the model of luminescence with SpectraMax iD3 multi-mode microplate reader (Molecular Devices). Nisin was used as a positive control at a final concentration of 100 μg/mL.

18. Antifungal Assays. The in vitro antifungal activity against five plant-pathogenic fungi (*Rhizoctonia solani*, *Colletotrichum fructicola*, *Colletotrichum gloeosporioides*, *Fusarium graminearum*, and *Fusarium oxysporum*) was evaluated using a 96-well microdilution assay. Amphotericin B was used as a positive control. The dioxanopeptins A, B, and C in DMSO solutions were two-fold serially diluted vertically in 96-well plates in a volume of 100 μL to obtain final concentrations ranging from 64 to 0.5 μg/mL. Subsequently, 100 μL of the inoculated fungal suspension was added to each well. For the negative control, 2 μL of DMSO was added to the first well, and the subsequent operations were the same as those for the experimental groups. For the blank control, 2 μL of DMSO was added to the first well, and the PDB culture medium was used to perform a two-fold dilution up to the

last well. Finally, PDB culture medium was added to each well to a final volume of 200 μL. The plates were incubated at 30°C for 48 hours. The absorbance was measured at 600 nm using a microplate reader, and the MIC was defined as the lowest concentration at which the absorbance was the same as that of the blank control. All experiments were performed in three replicates.

Supplementary Table 1 | A-domain substrate specificity predictions across three tools (Top-3 candidates). Scores are method-specific and used only to rank candidates within each tool; scores are not directly comparable across tools.

Protein	Locus tag	Domain Start-End	NRPSTransformer Top-1/2/3 (score)	PARAS Top-1/2/3 (score)	PARASECT Top-1/2/3 (score)	Consensus/Agreement (Top-1)
Chitinimonas koreensis DSM 17726						
Ck-DxpC	*F559_RS0116905*	542-947	Val (0.7466)<br>Leu (0.2261)<br>Ile (0.01498)	Leu (0.338)<br>Val (0.183)<br>Ile (0.156)	Leu (0.887)<br>Val (0.768)<br>Ile (0.702)	Leu (2/3)
Ck-DxpB	*F559_RS28020*	493-902	Gln (0.8941)<br>Arg (0.06183)<br>Glu (0.02281)	Gln (0.494)<br>Glu (0.153)<br>Asp (0.058)	Gln (0.790)<br>Glu (0.742)<br>Arg (0.641)	Gln (3/3)
Ck-DxpB	*F559_RS28020*	1586-1979	Ala (0.9997)<br>Ser (7.968e-05)<br>Gly(6.009e-05)	Ala (0.478)<br>Ser (0.307)<br>Dehydrotryptophan (0.049)	Ser (0.853)<br>Ala (0.664)<br>Ile (0.336)	Ala (2/3)
Ck-DxpA	*F559_RS26275*	2102-2499	Gln (0.7579)<br>Pipecolate (0.1276)<br>Hxorn (0.03254)	Ala (0.131)<br>Leu (0.130)<br>Gly (0.098)	Ala (0.562)<br>Leu (0.518)<br>Gly (0.462)	Ala (2/3)
Tistlia consotensis DSM 21585						
Tc-DxpC	*CHC03_RS11320*	532-929	Ala (0.9058)<br>Val (0.0634)<br>Pro (0.0259)	Val (0.325)<br>Ala (0.240)<br>Leu (0.09)	Val (0.878)<br>Ala (0.774)<br>Ile (0.717)	Val (2/3)
Tc-DxpB	*CHC03_RS11325*	1023-1048	Ser (0.9999)<br>Dpg (1.512e-05)<br>Ala (1.345e-05)	Ser (0.987)<br>Ala (0.003)<br>Phe (0.003)	Ser (0.992)<br>Ala (0.172)<br>Gly (0.052)	Ser (3/3)
Tc-DxpA	*CHC03_RS11330*	3494-3899	Ala (0.9889)<br>Ser (0.0099)<br>Pro (0.0005)	Ser (0.263)<br>Ala (0.147)<br>Val (0.063)	Ser (0.737)<br>Ala (0.508)<br>Gly (0.349)	Ser (2/3)
Chromobacterium subtsuga DSM 17043						
Cs-DxpC	*U6115_RS10910*	533-937	Val (0.9999)<br>Thr (6.809e-05)<br>Gly (5.401e-05)	Leu (0.265)<br>Val (0.244)<br>Ile (0.128)	Val (0.867)<br>Ile (0.767)<br>Leu (0.672)	Val (2/3)

Cs-DxpB	*U6115_RS10915*	476-876	Orn (0.5679)<br>Arg (0.3546)<br>Lys (0.0460)	Lys (0.255)<br>Gln (0.190)<br>Arg (0.087)	Lys (0.714)<br>Orn (0.689)<br>Arg (0,688)	Lys (2/3)
Cs-DxpB	*U6115_RS10915*	1536-1932	Ala (0.9950)<br>Ser (0.0023)<br>Hxorn (0.0007)	Ala (0.369)<br>Ser (0.211)<br>Gln (0.044)	Ala (0.707)<br>Ser (0.601)<br>Ile (0.336)	Ala (3/3)
Cs-DxpA	*U6115_RS10920*	2176-2579	Hxorn (0.6164)<br>Gln (0.2259)<br>Ala (0.0720)	Ala (0.164)<br>Leu (0,150)<br>4-Hydroxyphenylglycine (0.076)	Ala (0.581)<br>Gly (0.409)<br>Val (0.399)	Ala (2/3)

Supplementary Table 2 | Bacterial strains used in this study.

Species	Strain designation	NCBI accession number	Source	Reference
Chitinimonas koreensis	DSM 17726	GCF_000428465.1	DSM	[35]
Chromobacterium subtsuga	DSM 17043	GCF_001676875.1	DSM	[36]
Tistlia consotensis	DSM 21585	GCF_900188055.1	DSM	[37]
Escherichia coli	ATCC 25922	-	ATCC	[38]
Escherichia coli	B2	-	Reference	[38]
Klebsiella pneumoniae	ATCC 43816	-	ATCC	[38]
Klebsiella pneumoniae	ATCC 43816 Δ*waaC*	-	Reference	[38]
Salmonella enterica subsp.enterica serovar Typhimurium	ATCC 14028	-	ATCC	[38]
Acinetobacter baumannii	7-2	-	Reference	[38]
Acinetobacter baumannii	7-2<br>LPS deficiency	-	Reference	[38]
Staphylococcus aureus	ATCC 29213	-	ATCC	[34]
Staphylococcus aureus	T144	-	Reference	[34]
Staphylococcus aureus	USA300	-	Reference	[34]
Staphylococcus aureus	ATCC 43300	-	ATCC	[34]
Enterococcus faecium	BM1405	-	Reference	[39]
Enterococcus faecium	Vancomycin resistant strains: CAU 362, CAU 367, CAU 370, CAU 375, and CAU 376.	-	Reference	[39]
Enterococcus faecalis	CAU 469	-	Reference	[39]
Enterococcus faecalis	ATCC 29212	-	ATCC	[39]
Pseudomonas aeruginosa	PAO1	-	Reference	[40]

ATCC: American Type Culture Collection; DSM: German Collection of Microorganisms and Cell Cultures.

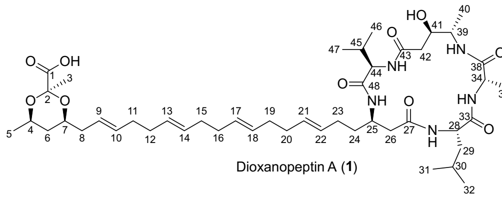

Dioxanopeptin A (**1**)

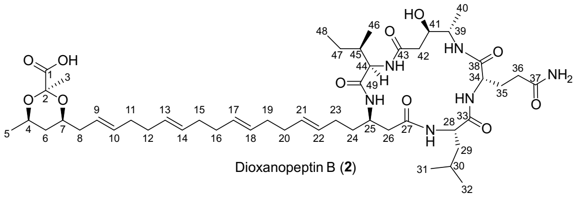

Dioxanopeptin B (**2**)

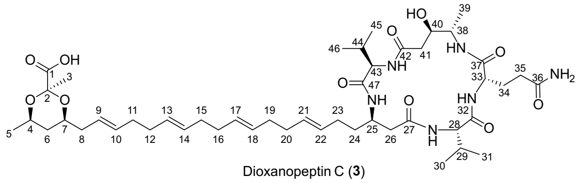

Dioxanopeptin C (**3**)

Supplementary Table 3 | ^{1}H (700 MHz) and ^{13}C (175 MHz) NMR data assignments for dioxanopeptins A–C (**1**–**3**) in DMSO-d_6 (for NMR spectra see Supplementary Figures 25–49)

	No.	Dioxanopeptin A (**1**)		Dioxanopeptin B (**2**)		No.	Dioxanopeptin C (**3**)	
		δ_H (mult., *J*)	δ_C (mult., *J*)	δ_H (mult., *J*)	δ_C (mult., *J*)		δ_H (mult., *J*)	δ_C (mult., *J*)
PUFA								
	1	-	173.2, C	-	173.2, C	1	-	173.0 C
	2	-	97.8, C	-	97.8, C	2	-	97.8, C
	3	1.46 (s)	17.5, CH_3	1.46 (s)	17.5, CH_3	3	1.46 (s)	17.5, CH_3
	4	3.98 (m)	64.8, CH	3.99 (m)	64.8, CH	4	3.96 (m)	64.6, CH
	5	1.07 (d, 6.0)	22.0, CH_3	1.07 (d, 6.0)	22.0, CH_3	5	1.07 (d, 6.0)	22.0, CH_3
	6	0.99 (m)	38.0, CH_2	0.98 (m)	37.9, CH_2	6	0.99 (m)	38.0, CH_2
		1.48 (m)		1.50 (m)			1.48 (m)	
	7	3.86 (m)	68.5, CH	3.86 (m)	68.6, CH	7	3.83 (m)	68.5, CH
	8	2.18 (m)	39.1, CH_2	2.19 (m)	39.1, CH_2	8	2.19 (m)	39.1, CH_2
		1.98 (m)		1.99 (m)			1.98 (m)	
	9	5.38 (m)	125.9, CH	5.38 (m)	125.7, CH	9	5.38 (m)	126.1, CH
	10	5.42 (m)	131.9, CH	5.42 (m)	132.0, CH	10	5.42 (m)	131.8, CH
	11	1.99 (m)	32.1, CH_2	2.00 (m)	32.1, CH_2	11	1.99 (m)	32.1, CH_2
	12	1.99 (m)	32.1, CH_2	2.00 (m)	32.1, CH_2	12	1.99 (m)	32.1, CH_2
	13	5.37 (m)	129.7, CH	5.37 (m)	129.7, CH	13	5.37 (m)	129.7, CH
	14	5.37 (m)	129.7, CH	5.37 (m)	129.7, CH	14	5.37 (m)	129.7, CH
	15	1.99 (m)	32.1, CH_2	1.98 (m)	32.1, CH_2	15	1.99 (m)	32.1, CH_2
	16	1.99 (m)	32.1, CH_2	1.98 (m)	32.1, CH_2	16	1.99 (m)	32.1, CH_2
	17	5.37 (m)	129.8, CH	5.37 (m)	129.7, CH	17	5.37 (m)	129.8, CH
	18	5.37 (m)	129.7, CH	5.37 (m)	129.7, CH	18	5.37 (m)	129.7, CH
	19	1.99 (m)	32.1, CH_2	1.98 (m)	32.1, CH_2	19	1.99 (m)	32.1, CH_2
	20	1.99 (m)	32.1, CH_2	1.98 (m)	32.1, CH_2	20	1.99 (m)	32.1, CH_2
	21	5.37 (m)	129.7, CH	5.37 (m)	129.7, CH	21	5.37 (m)	129.7, CH
	22	5.37 (m)	129.6, CH	5.37 (m)	129.7, CH	22	5.37 (m)	129.6, CH
	23	1.91 (m)	29.2, CH_2	1.91 (m)	29.2, CH_2	23	1.91 (m)	29.1, CH_2
	24	1.29 (m)	33.2, CH_2	1.30 (m)	33.2, CH_2	24	1.32 (m)	33.0, CH_2
		1.33 (m)		1.35 (m)			1.40 (m)	
	25	3.86 (m)	45.9, CH	3.87 (m)	45.9, CH	25	3.83 (m)	45.6, CH
	25-NH	8.09 (d, 8.4)	-	8.15 (d, 8.6)	-	25-NH	8.05 (d, 8.4)	-
	26	2.45 (m)	37.1, CH_2	2.45 (m)	37.1, CH_2	26	2.47 (m)	36.8 CH_2
		2.30 (m)		2.30 (m)			2.35 (m)	
	27	-	173.0, C	-	172.2, C	27	-	173.0, C
Leu/Val								
	28	3.98 (m)	52.7, CH	3.99 (m)	52.8, CH	28	4.00 (m)	52.7, CH
	28-NH	8.55 (m)	-	8.43 (m)	-	28-NH	8.44 (m)	-
	29	1.56 (m)	39.4, CH_2	1.56 (m)	39.4, CH_2	-	-	-
		1.49 (m)		1.49 (m)				
	30	1.72 (m)	24.3, CH	1.72 (m)	24.3, CH	29	1.72 (m)	24.3, CH
	31	0.79 (d, 6.5)	20.4, CH_3	0.78 (d, 6.5)	20.4, CH_3	30	0.90 (d, 6.5)	18.9 CH_3
	32	0,89 (d, 6.4)	23.1, CH_3	0,89 (d, 6.4)	23.4, CH_3	31	0,89 (d, 6.4)	19. 3, CH_3
	33	-	172.1, C	-	172.1, C	32	-	172.1, C
Gln								
	34	4.06 (m)	54.7, CH	4.06 (m)	54.8, CH	33	4.06 (m)	54.7, CH
	34-NH	8.22 (m)	-	7.99 (m)	-	33-NH	8.17 (m)	-

	35	1.83 (m)	27.5, CH_2	1.84 (m)	27.4, CH_2	34	1.83 (m)	27.5, CH_2
	36	1.98 (m)	31.8, CH_2	1.98 (m)	31.7, CH_2	35	1.98 (m)	31.8, CH_2
	37	-	173.6, C	-	173.6, C	36	-	173.7, C
	37-NH_2	6.70 (s)	-	6.72 (s)	-	36-NH_2	6.70 (s)	-
	38	-	171.2, C	-	171.2, C	37	-	171.2, C
Ala-polyketide								
	39	3.42 (m)	49.4, CH	3.42 (m)	49.4, CH	38	3.42 (m)	49.4, CH
	39-NH	7.62 (s)	-	7.55 (s)	-	38-NH	7.58 (s)	-
	40	1.02 (d, 6.4)	17.0, CH_3	1.03 (d, 6.3)	17.1, CH_3	39	1.02 (d, 6.4)	16.7, CH_3
	41	3.86 (m)	69.5, CH	3.86 (m)	69.5, CH	40	3.88 (m)	69.5, CH
	42	2.10 (m)<br>2.07 (m)	39.8, CH_2	2.04 (m)<br>2.10 (m)	40.0, CH_2	41	2.10 (m)<br>2.07 (m)	39.8, CH_2
	43	-	172.0, C	-	172.0, C	42	-	171.9, C
Val/Ile								
	44	3.76 (m)	60.8, CH	3.99 (m)	58.2, CH	43	3.78 (m)	60.6, CH
	44-NH	8.14 (d, 6.6)	-	8.08 (d, 6.8)	-	43-NH	8.15 (d, 6.6)	-
	45	1.96 (m)	28.7, CH	1.78 (m)	35.0, CH	44	1.93 (m)	28.9, CH
	46	0.90 (d, 7.0)	19.3, CH_3	0.85 (d, 7.0)	14.8, CH_3	45	0.88 (d, 7.0)	19.0, CH_3
	47	0.88 (d, 7.0)	18.5, CH_3	1.17 (m)<br>1.34 (m)	25.7, CH_2	46	0.89 (d, 7.0)	18.5, CH_3
	48	-	170.6, C	0.82 (t, 6.6)	11.6, CH_3	47	-	170.6, C
	49			-	170.7, C			

Supplementary Table 4 | HR-ESI-MS data of all compounds described in this work.

**Compound**	**Detected mass**	**Calculated mass**	**Error (ppm)**	**Ion formula**
Dioxanopeptin A (**1**)	915.5804 $[M + H]^+$	915.5701 $[M + H]^+$	0.3	$C_{48}H_{79}N_6O_{11}$ $[M + H]^+$
Dioxanopeptin B (**2**)	929.5968 $[M + H]^+$	929.5957 $[M + H]^+$	1.1	$C_{49}H_{81}N_6O_{11}$ $[M + H]^+$
Dioxanopeptin C (**3**)	901.5656 $[M + H]^+$	901.5644 $[M + H]^+$	1.2	$C_{47}H_{77}N_6O_{11}$ $[M + H]^+$
Dioxanopeptin D (**4**)	945.5917 $[M + H]^+$	945.5907 $[M + H]^+$	1.1	$C_{49}H_{81}N_6O_{12}$ $[M + H]^+$
Dioxanopeptin E (**5**)	947.6083 $[M + H]^+$	947.6063 $[M + H]^+$	2.1	$C_{49}H_{83}N_6O_{12}$ $[M + H]^+$
Dioxanopeptin F (**6**)	933.5922 $[M + H]^+$	933.5907 $[M + H]^+$	0.3	$C_{48}H_{81}N_6O_{12}$ $[M + H]^+$
Dedioxanopeptin A (**7**)	845.5774 $[M + H]^+$	845.5746 $[M + H]^+$	3.2	$C_{45}H_{77}N_6O_9$ $[M + H]^+$
Dedioxanopeptin B (**8**)	859.5931 $[M + H]^+$	859.5903 $[M + H]^+$	3.3	$C_{46}H_{79}N_6O_9$ $[M + H]^+$
Dedioxanopeptin C (**9**)	831.5619 $[M + H]^+$	831.5590 $[M + H]^+$	3.5	$C_{44}H_{75}N_6O_9$ $[M + H]^+$
Dedioxanopeptin D (**10**)	817.5482 $[M + H]^+$	817.5433 $[M + H]^+$	5.9	$C_{43}H_{73}N_6O_9$ $[M + H]^+$

Supplementary Table 5 | Putative functional assignments of the Ck-*dxp* BGC in *Chitinimonas koreensis* DSM 17726.

**Protein**	**Locus tag**	**Length (aa)**	**Domain Architecture**	**Reference protein function**	**Reference accession**	**Identity/ Coverage (%)**
Ck-DxpA	*F559_RS26275*	2715	KS–AT–ACP–AMT–$^{L}C_{L}$–A–T	Mycosubtilin synthase subunit A	AAF08795.1	32/85
Ck-DxpB	*F559_RS28020*	3568	$^{L}C_{L}$–A–T–$^{L}C_{L}$–A–T–KS–AT–KR–ACP	Tyrocidine synthase III	AAC45930.1	35/61
Ck-DxpC	*F559_RS0116905*	2379	$^{L}C_{L}$–A–T–E–$^{D}C_{L}$–T–TE	Linear gramicidin synthase subunit C	CAD92851.1	34/88
Ck-DxpD	*F559_RS0116910*	2176	KS–AT–ACP–ACP–ACP–ACP–KR	Omega-3 polyunsaturated fatty acid synthase subunit	AAN54658.1	36/90
Ck-DxpE	*F559_RS0116915*	1837	KS–CLF–DH	Multi-domain beta-ketoacyl synthase	AAN54656.1	35/61
Ck-DxpF	*F559_RS26285*	602	2-Nitropropane dioxygenase (NPD) like domain–ACP	Omega-3 polyunsaturated fatty acid synthase subunit	AAN54654.1	53/74
Ck-DxpG	*F559_RS0116930*	265	TE	Gramicidin S biosynthesis protein GrsT	AAA58717.1	35/91
Ck-DxpH	*F559_RS0116935*	477	Polysaccharide pyruvyl transferase	Polysaccharide pyruvyl transferase family protein WcaK	SFJ25624.1	59/99

Supplementary Table 6 | Stereochemistry assignment of amino acid residues based on NRPS domain annotations and Marfey's analysis.

Amino acid residue in dioxanopeptins	NRPS domain	C domain subtype	Carbon position	Predicted configuration	Marfey's assay-determined configuration
Leucine	Ck-DxpA C_2	$^{L}C_{L}$	C-28	L	L
Glutamine	Ck-DxpB C_3	$^{L}C_{L}$	C-34	L	L
Alanine	Ck-DxpB C_4	$^{L}C_{L}$	C-39	L	N.A.
Valine/Isoleucine	Ck-DxpC E–C_5	$^{D}C_{L}$	C-44	D	D

For hydrolysis-accessible canonical amino acids, absolute configurations were determined by Marfey's derivatization (see Supplementary Figure 18). Predicted configurations were inferred from antiSMASH annotations of the downstream C domain subtype and from the presence/absence of E domains. The alanine-derived, C2-extended residue lacks an authentic standard and therefore, was not assigned by Marfey's analysis, and its configuration was inferred from domain annotations.

Supplementary Table 7 | Antibacterial activities of dioxanopeptin A (**1**) against VRE and MRSA.

Species	Strains	**1**	**Vancomycin**
		MICs	
E. faecium	CAU 362	2	>128
	CAU 367	2	>128
	CAU 370	2	>128
	CAU 375	4	>128
	CAU 376	2	>128
E. faecalis	ATCC 29212	2	0.5
	CAU 469	2	>128
S. aureus	USA300	8	0.5
	ATCC 43300	4	0.25

MICs, μg/mL

Supplementary Table 8 | Antifungal activities of dioxanopeptins A–C (**1**–**3**) against Plant-pathogenic Fungi.

Species	**1**	**2**	**3**	**Amphotericin B**
	MICs			
Rhizoctonia solani	32	>64	32	<0.5
Colletotrichum fructicola	>64	>64	64	1
Colletotrichum gloeosporioides	>64	>64	>64	4
Fusarium graminearum	>64	>64	>64	8
Fusarium oxysporum	>64	>64	>64	8

MICs, μg/mL

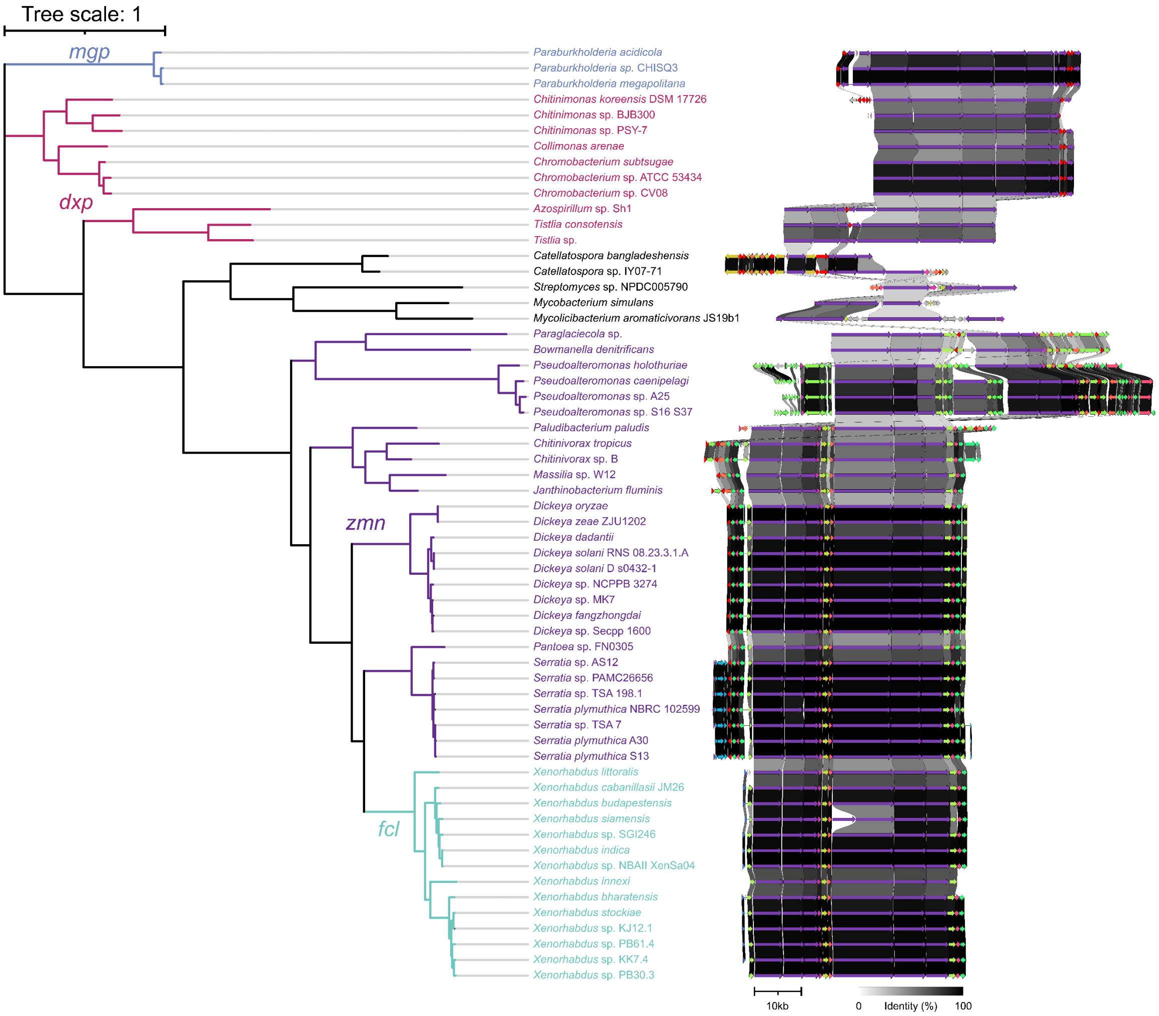

Supplementary Figure 1 | Phylogenetic and synteny analyses PUFAS–NRPS BGCs found in bacteria domain. Maximum-likelihood phylogeny (left) of 60 PUFAS–NRPS BGCs inferred from a concatenated core-gene alignment comprising *pfaA*, *pfaC*, and *pfaD* together with the co-localized NRPS component. Phylogenetic inference used the Le and Gascuel (LG) amino-acid replacement model with a proportion of invariant sites (I), gamma-distributed rate heterogeneity across sites with four discrete categories (G4), and empirical amino-acid frequencies (F); node support was assessed with 1,000 bootstrap replicates. Branch lengths represent amino-acid substitutions per site (tree scale shown). Major clades are labelled on the tree as *mgp*, *dxp*, *zmn*, and *fcl*. Gene-cluster architecture is shown to the right in the same order as the phylogeny, with arrows indicating predicted coding sequences and transcriptional orientation. Synteny across the 60 BGCs was evaluated using clinker; homologous genes are connected by shaded links whose intensity reflects amino-acid identity, summarizing conserved gene order and local rearrangements among clusters.

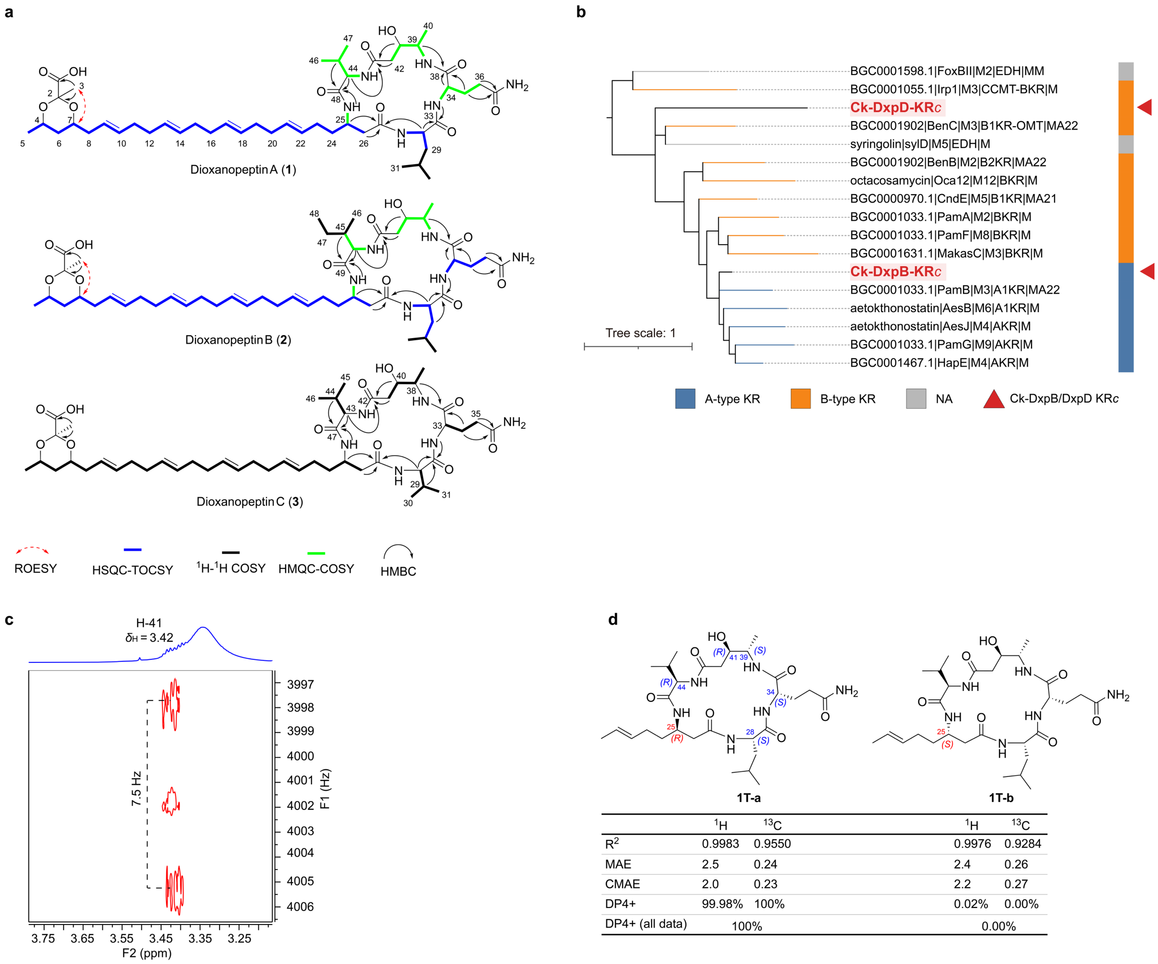

	^{1}H	^{13}C	^{1}H	^{13}C
R^2	0.9983	0.9550	0.9976	0.9284
MAE	2.5	0.24	2.4	0.26
CMAE	2.0	0.23	2.2	0.27
DP4+	99.98%	100%	0.02%	0.00%
DP4+ (all data)	100%		0.00%	

Supplementary Figure 2 | Structure elucidation of dioxanopeptins A–C (**1**–**3**). (a) Key 2D NMR correlations to establish the planar structure. (b) Global maximum-likelihood phylogeny of the catalytic subdomains of KR (KR_C) in Ck-DxpB and Ck-DxpD (see also Supplementary Figure 3). (c) PSYCHEDELIC 2D *J* spectrum of dioxanopeptin B (**2**) recorded with selective 180° pulses applied to H-41 (δ_H 3.86) to retain only couplings involving H-41, while suppressing other homonuclear couplings in F2 (pure-shift dimension). The H-39 resonance shows a doublet splitting along F1, from which $^{3}J_{H39–H-41}$ = 7.5 Hz was obtained. By Karplus-type correlations, a vicinal ^{3}J in this range is an *anti* H39–C39–C41–H41 dihedral angle, which supports the assigned stereochemistry of 41R and is consistent with the KR_C-based prediction. (d) Comparison of experimental and DFT-calculated ^{13}C chemical shifts supporting the assigned stereochemistry of 25*R*.

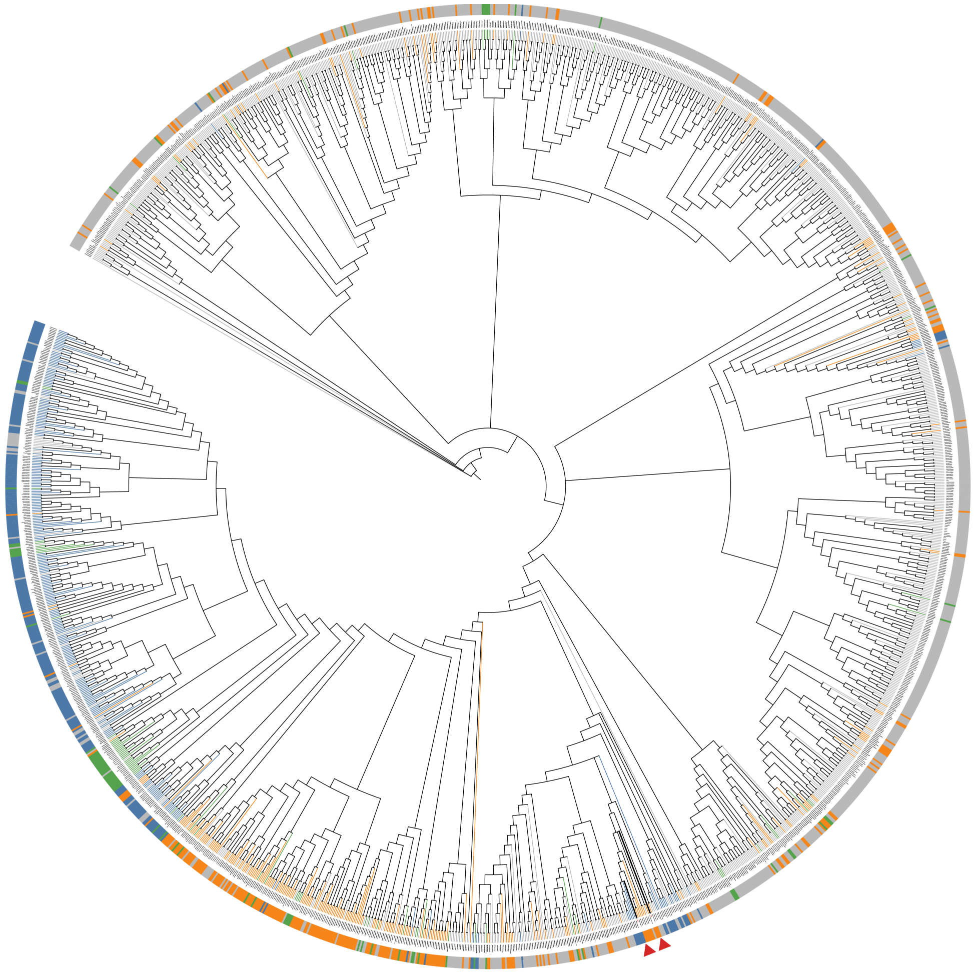

A-type KR B-type KR C-type KR NA Ck-DxpB/DxpD KR*c*

Supplementary Figure 3 | Global maximum-likelihood phylogeny of the catalytic subdomains of KR (KR_{C}) in Ck-DxpB and Ck-DxpD. The phylogeny of the KR_{C} is shown with reference to KR_{C} sequences with experimentally characterized KR stereochemical outcomes. Motif-based KR stereochemical fingerprints and their mechanistic basis have been established in earlier studies and structural analyses. Given that these fingerprint rules were developed primarily from modular bacterial PKSs and have recognized context dependence, stereochemical assignments were inferred from the clade placement of the query sequences within the reference KR_{C} phylogeny and reported with explicit confidence grading based on bootstrap support. CK-DxpD is assigned as B-type with higher confidence, supported by its phylogenetic placement (Bootstrap = 72). CK-DxpB is assigned as A-type with moderate confidence based on its phylogenetic placement within A-type reference clades (Bootstrap = 56).

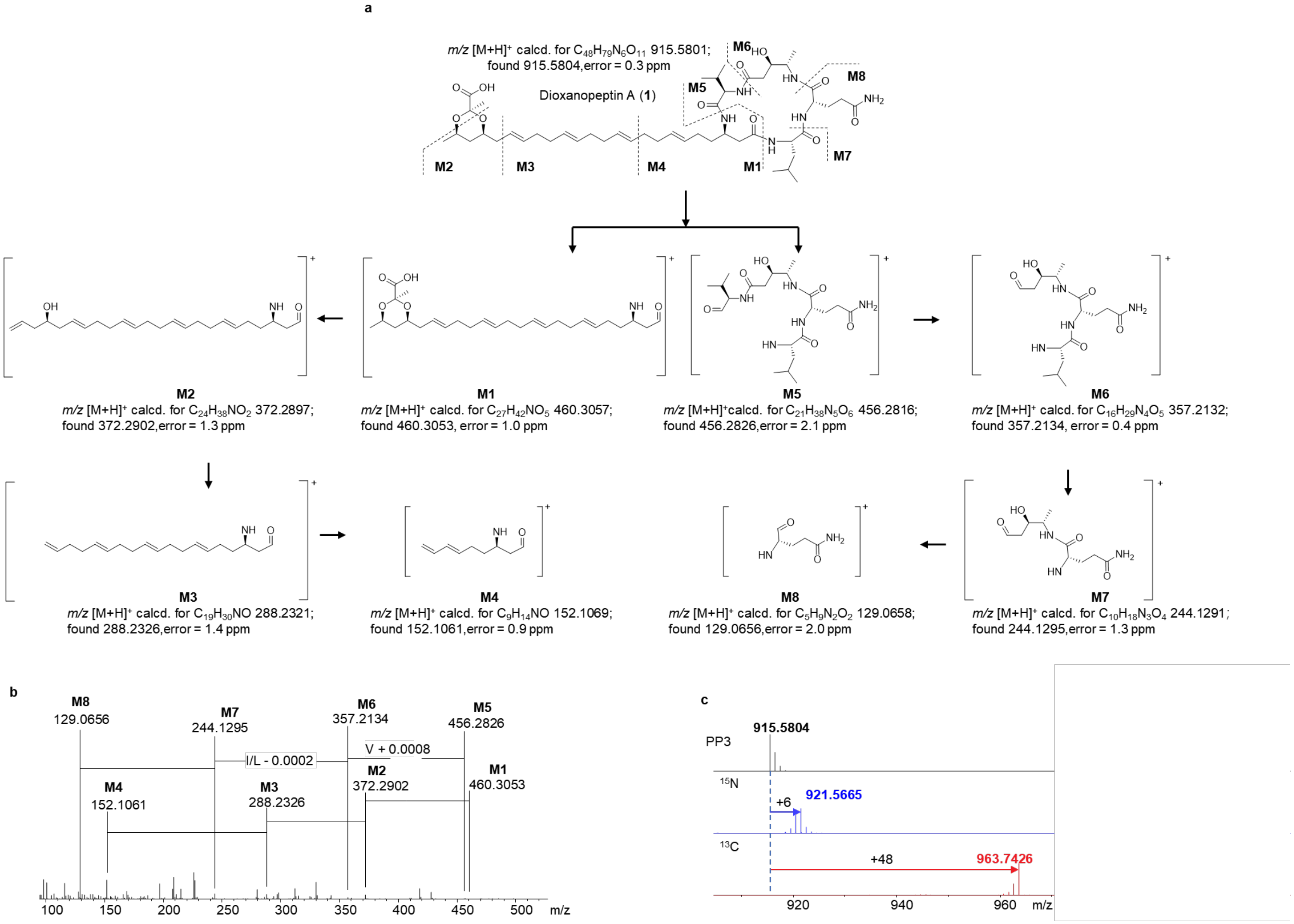

Supplementary Figure 4 | MS/MS fragmentation patterns and isotope labeling experiments for dioxanopeptin A (**1**).

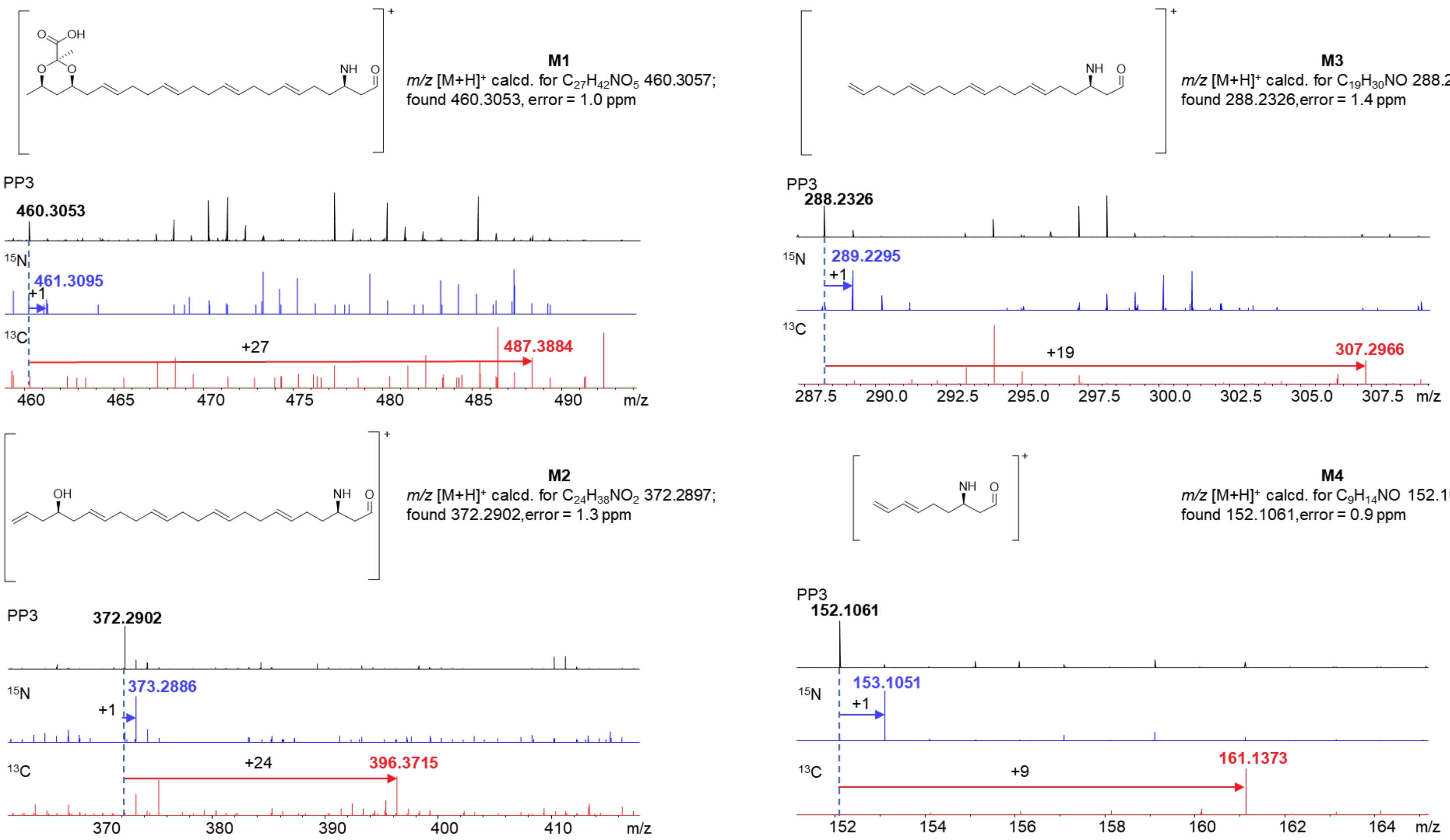

Supplementary Figure 5 | MS/MS fragmentation patterns of the lipid tail (**M1–M4**) of dioxanopeptin A (**1**).

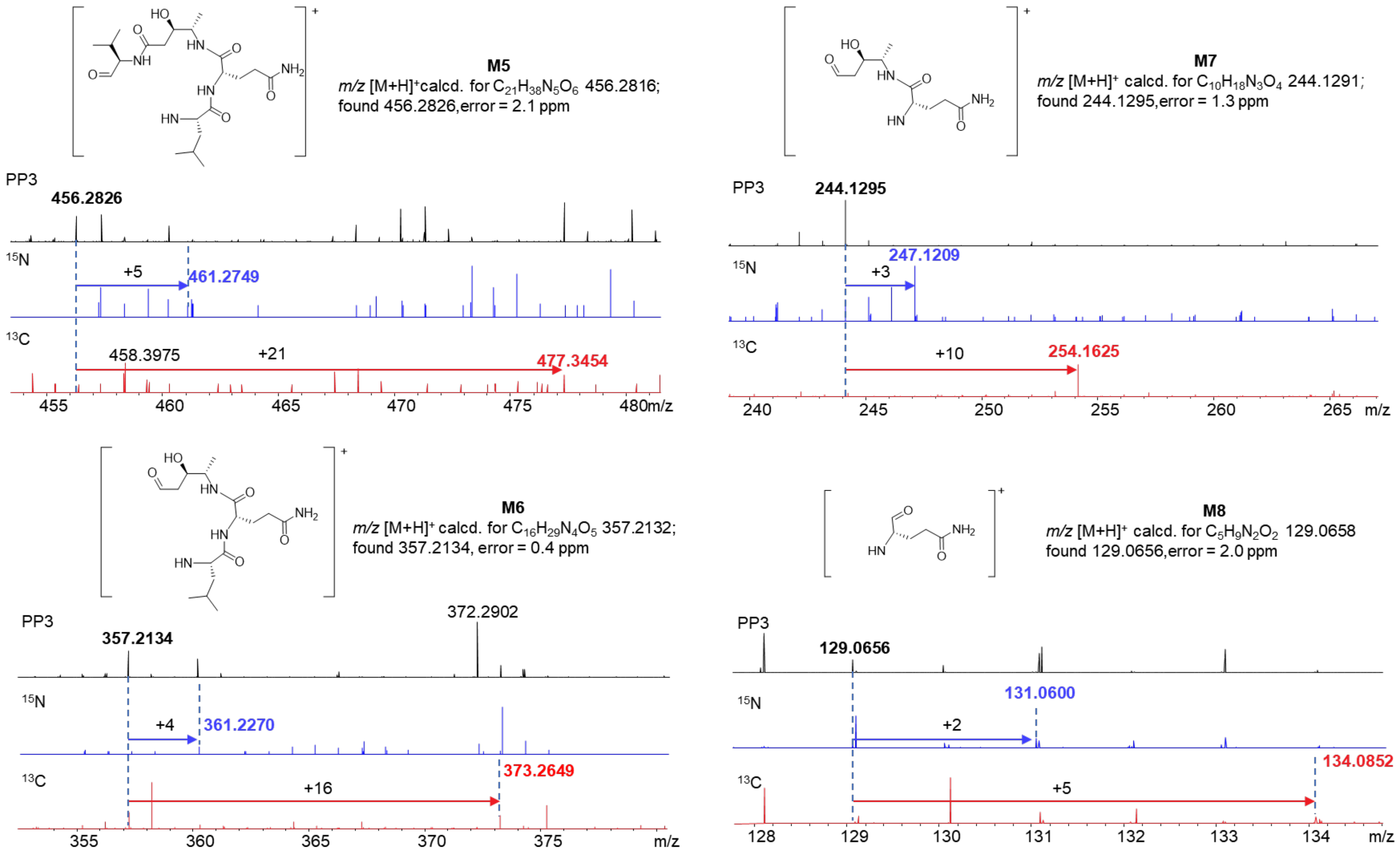

Supplementary Figure 6 | MS/MS fragmentation patterns of the peptide moiety (**M5–M8**) of dioxanopeptin A (**1**).

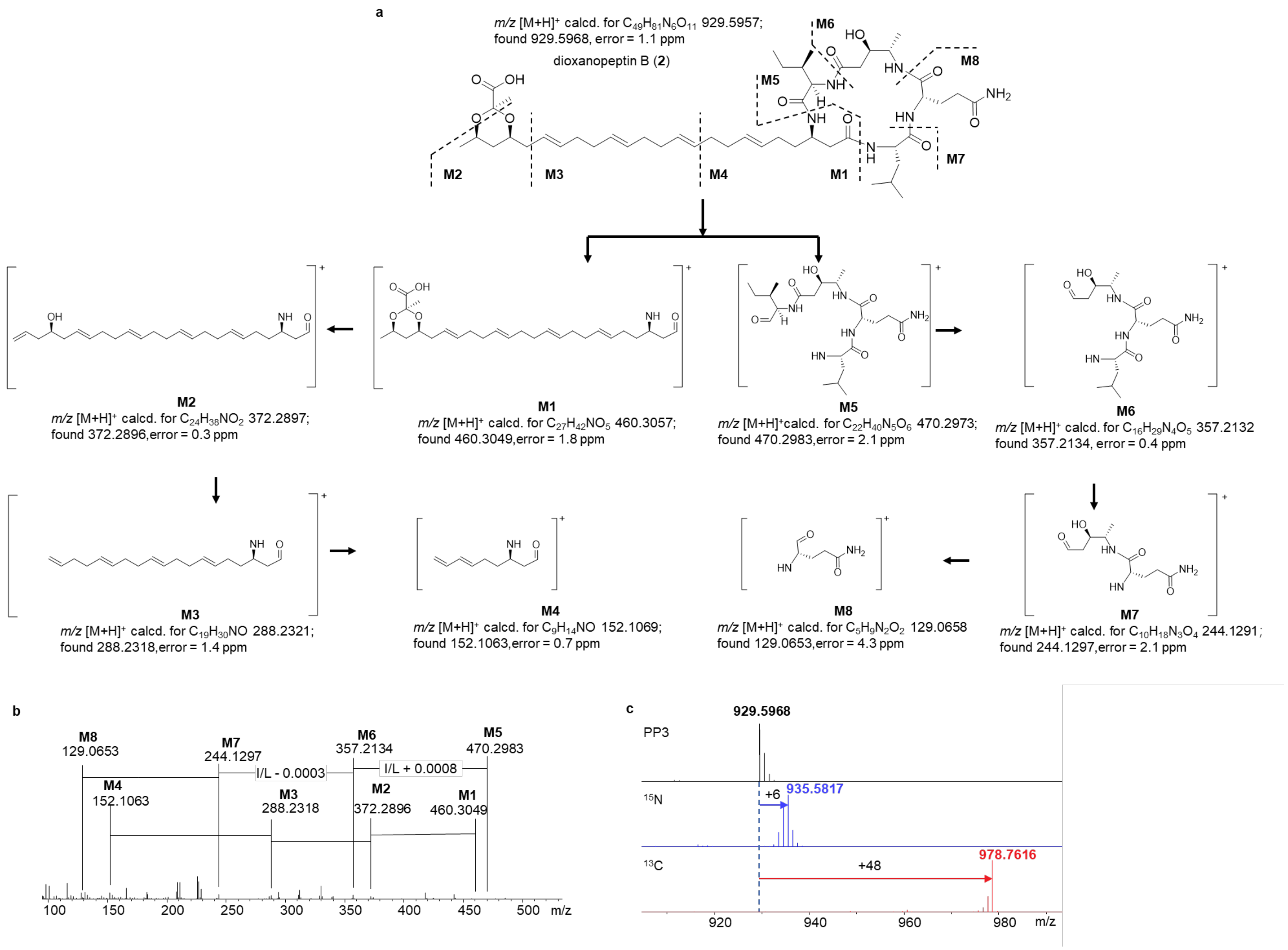

Supplementary Figure 7 | MS/MS fragmentation patterns and isotope labeling experiments for dioxanopeptin B (**2**).

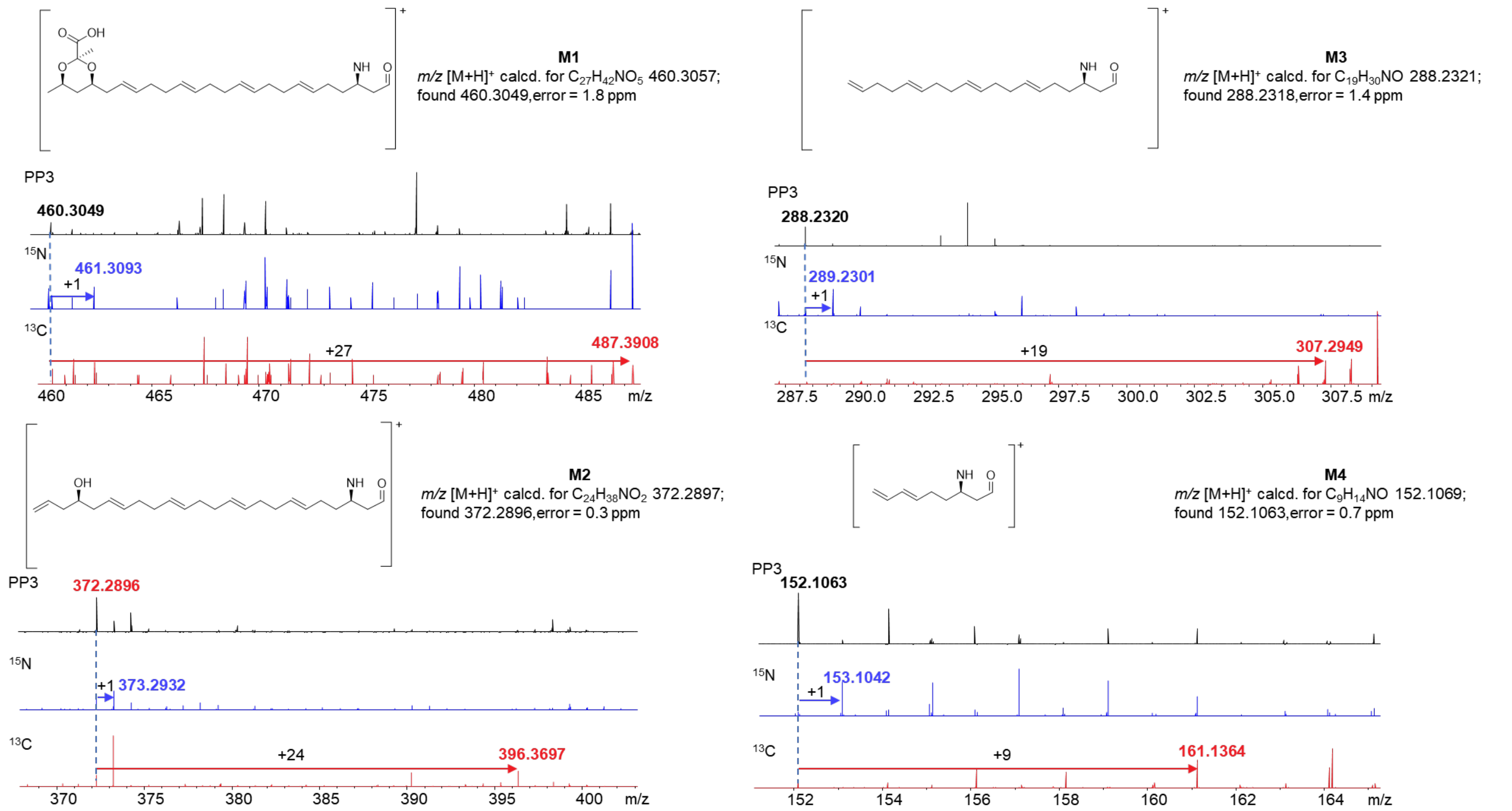

Supplementary Figure 8 | MS/MS fragmentation patterns of the lipid tail (**M1–M4**) of dioxanopeptin B (**2**).

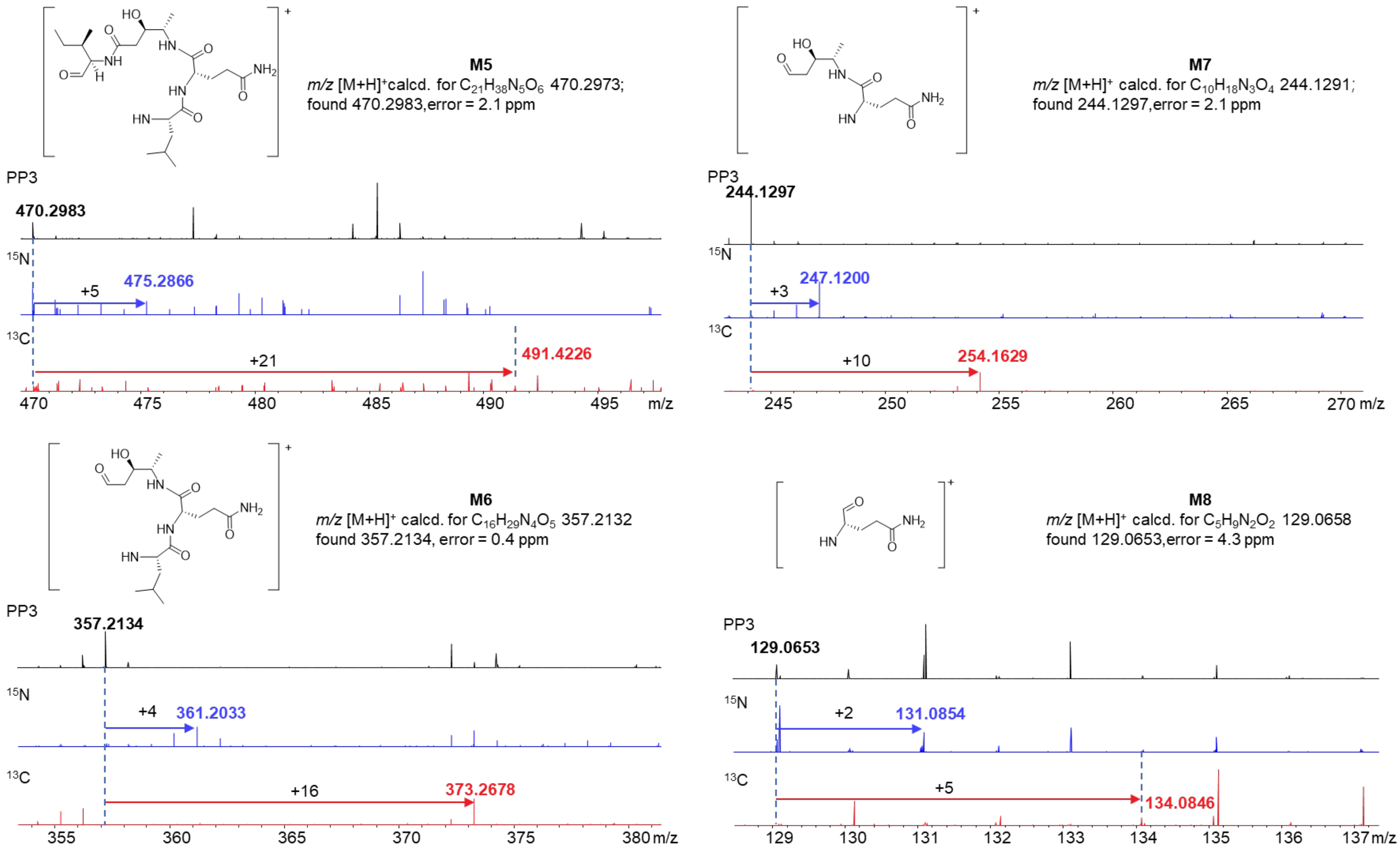

Supplementary Figure 9 | MS/MS fragmentation patterns of the peptide moiety (**M5–M8**) of dioxanopeptin B (**2**).

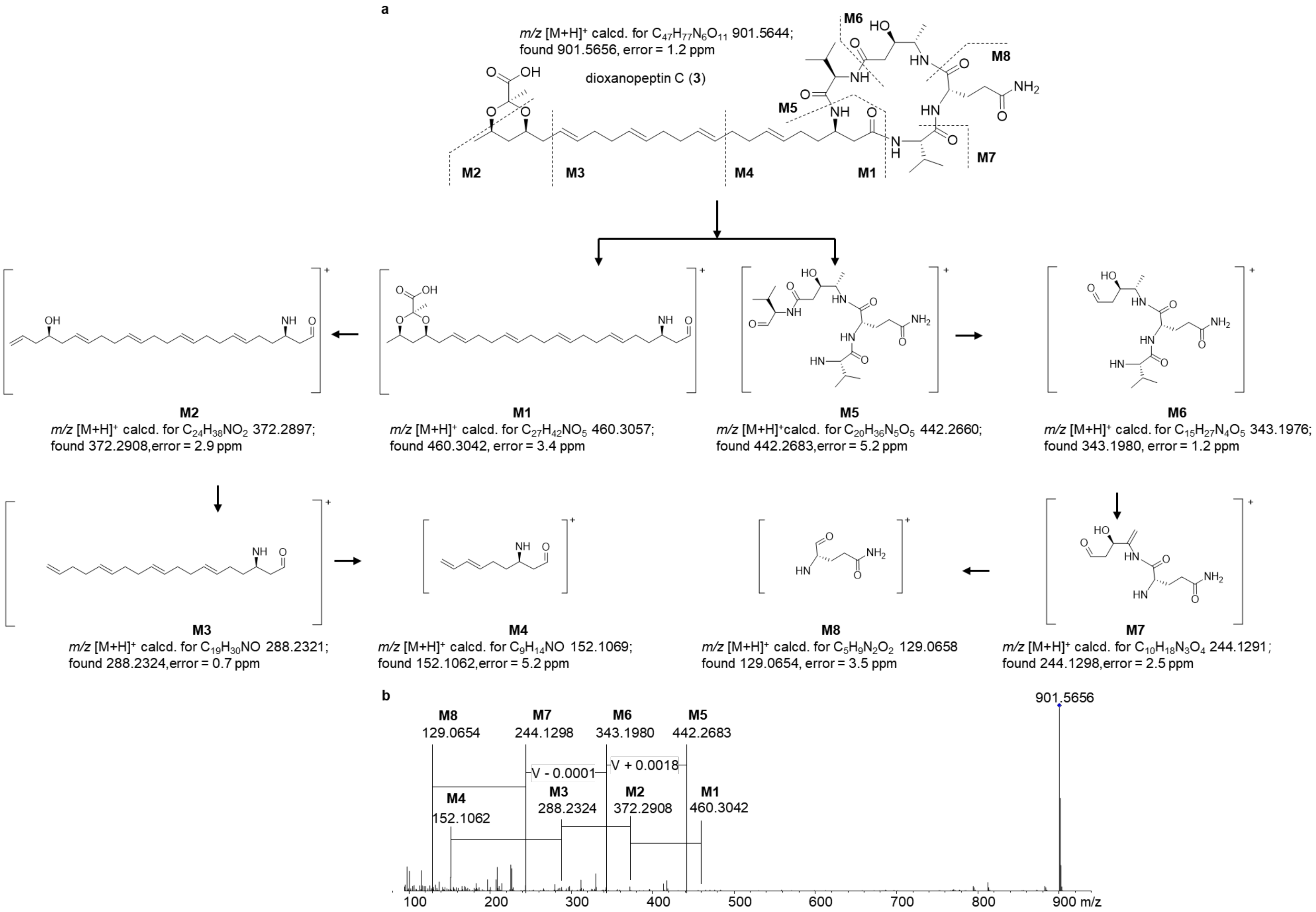

Supplementary Figure 10 | MS/MS fragmentation patterns of dioxanopeptin C (**3**).

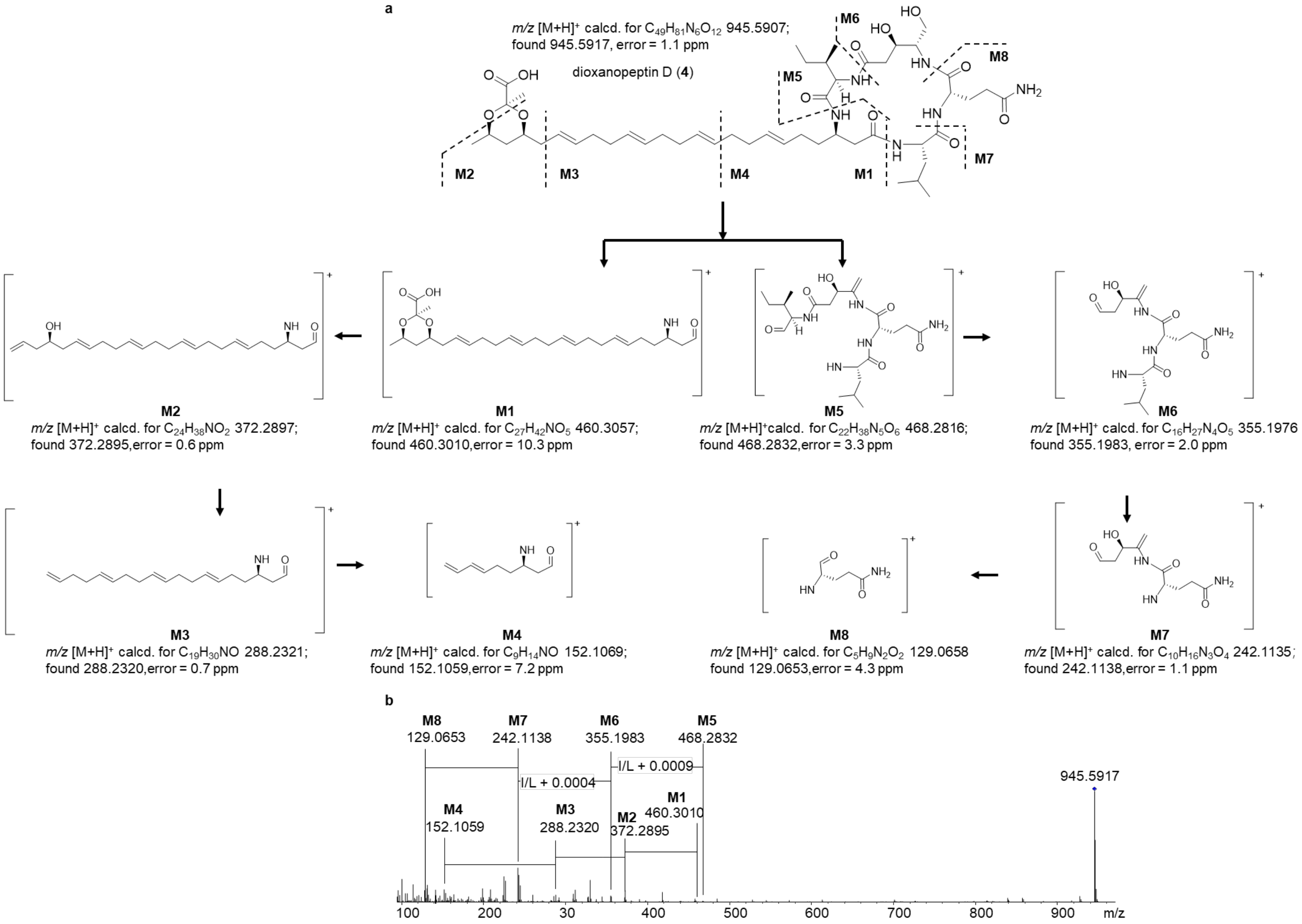

Supplementary Figure 11 | MS/MS fragmentation patterns of dioxanopeptin D (**4**).

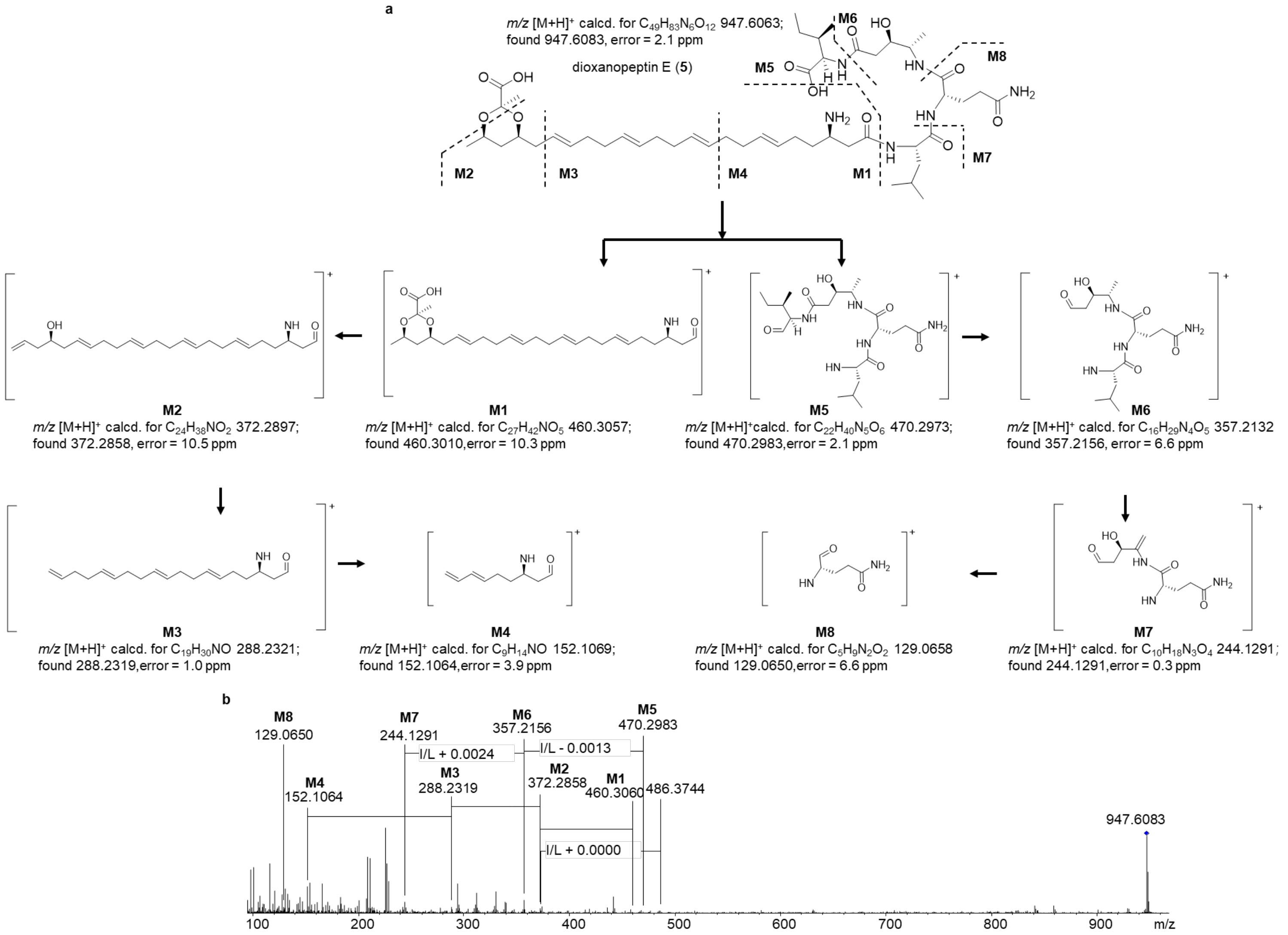

Supplementary Figure 12 | MS/MS fragmentation patterns of dioxanopeptin E (**5**).

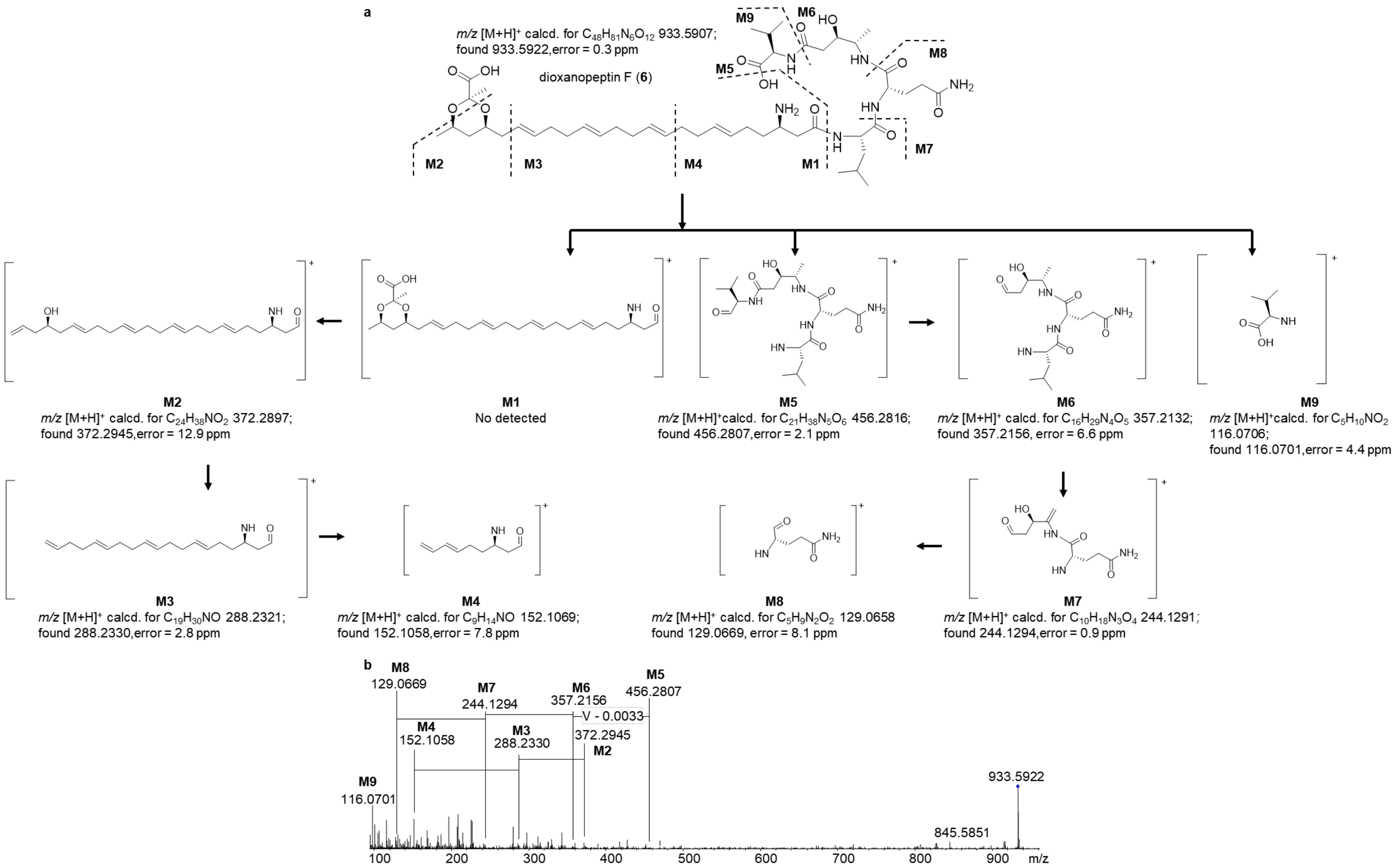

Supplementary Figure 13 | MS/MS fragmentation patterns of dioxanopeptin F (**6**).

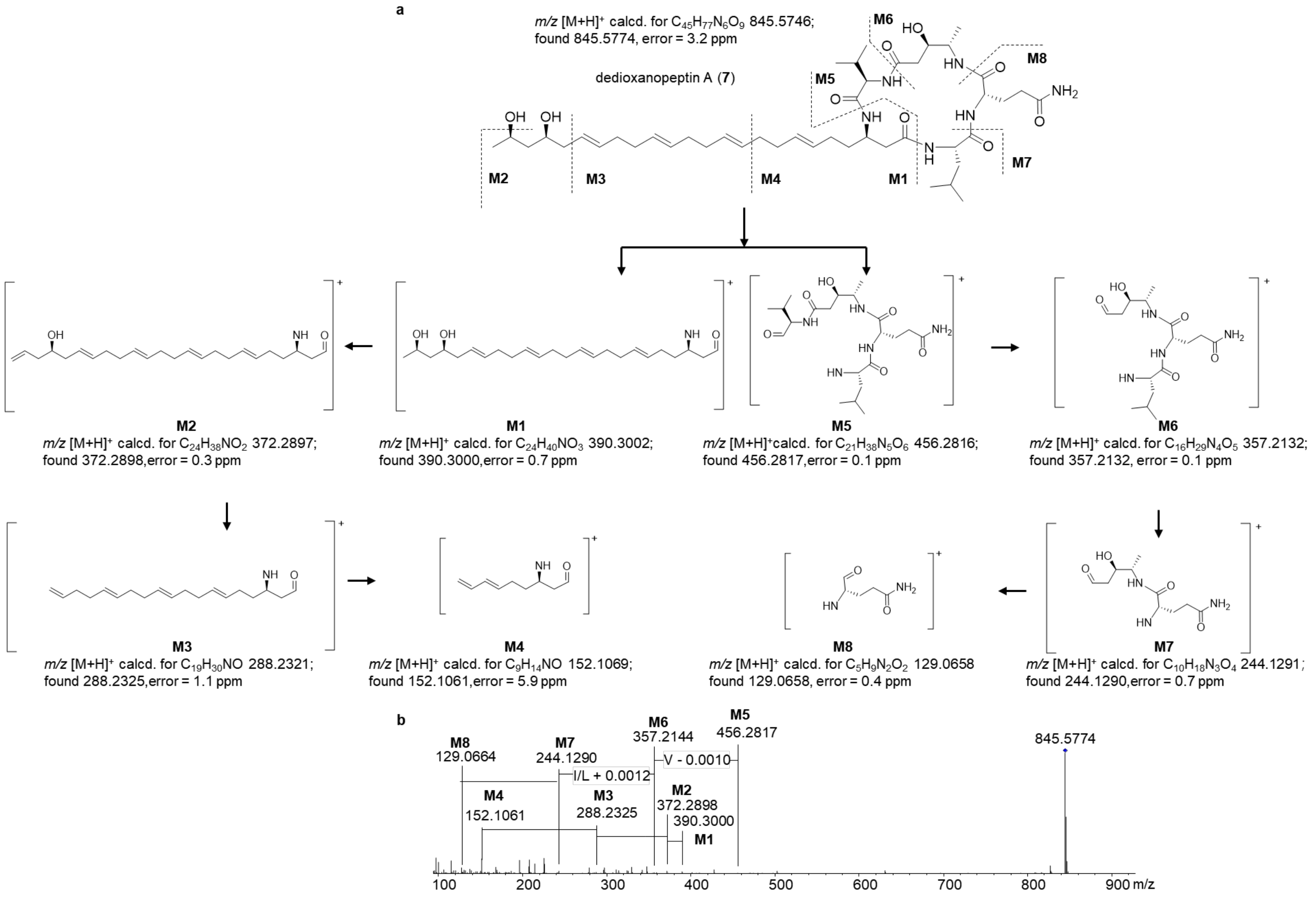

Supplementary Figure 14 | MS/MS fragmentation patterns of dedioxanopeptin A (**7**).

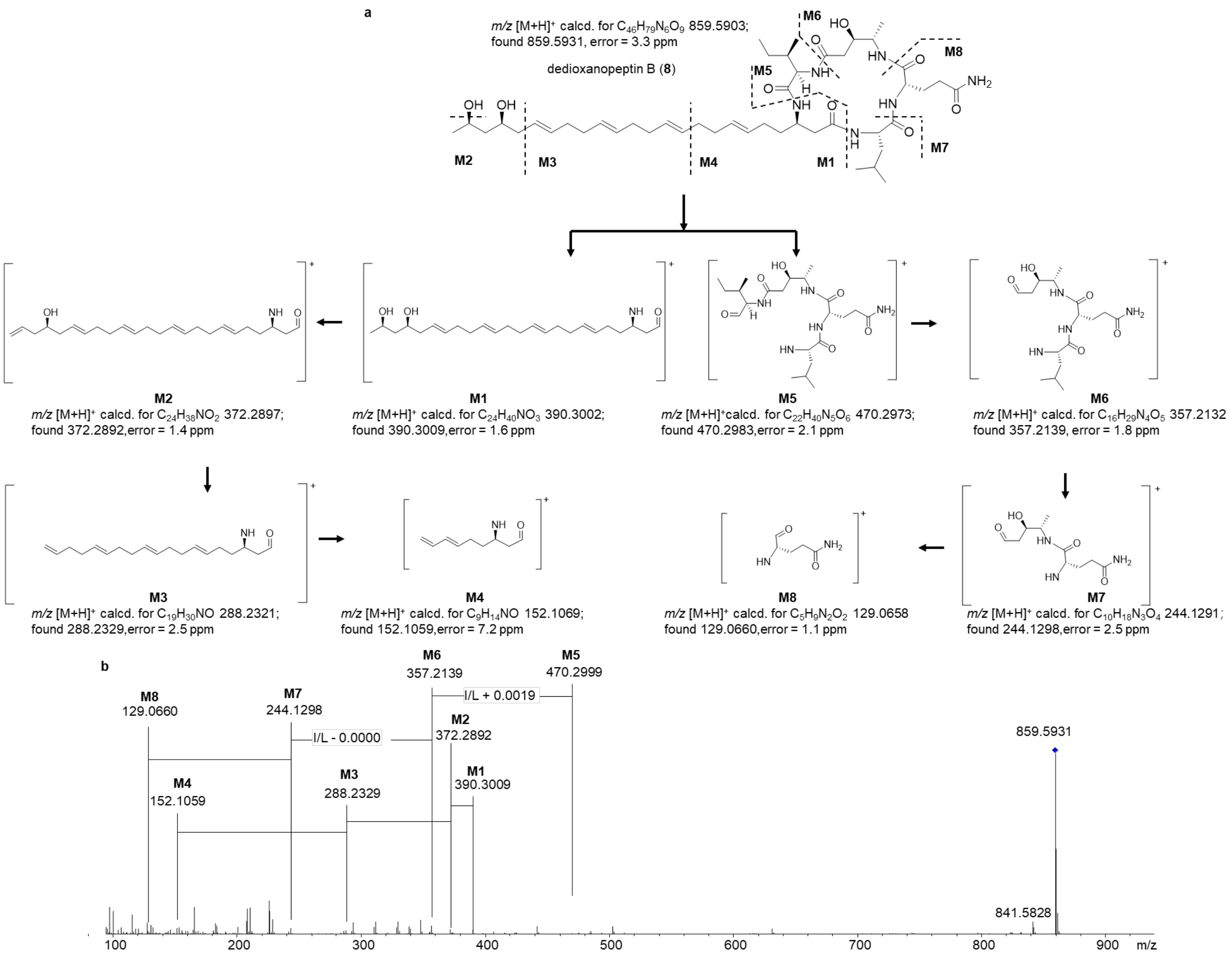

Supplementary Figure 15 | MS/MS fragmentation patterns of dedioxanopeptin B (**8**).

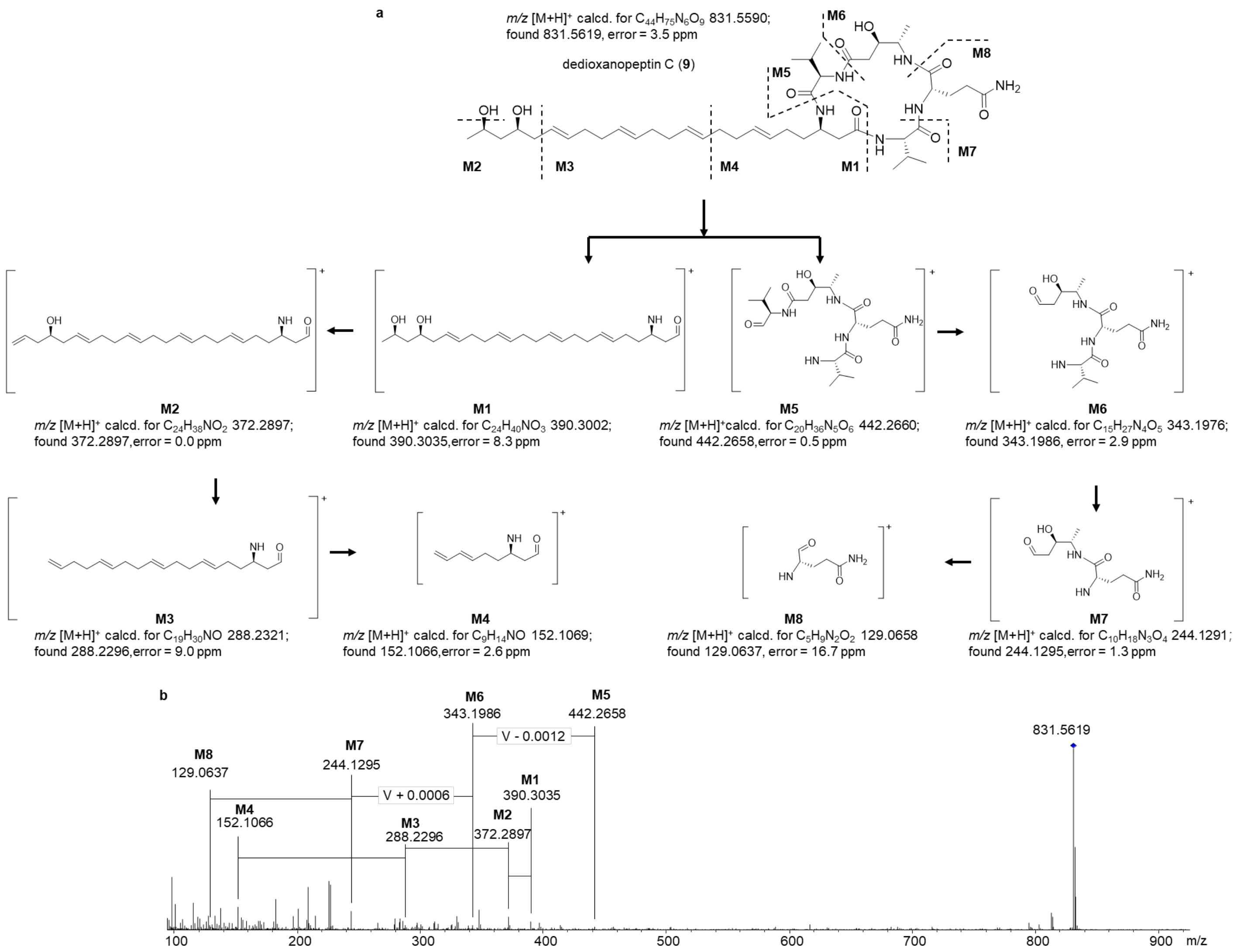

Supplementary Figure 16 | MS/MS fragmentation patterns of dedioxanopeptin C (**9**).

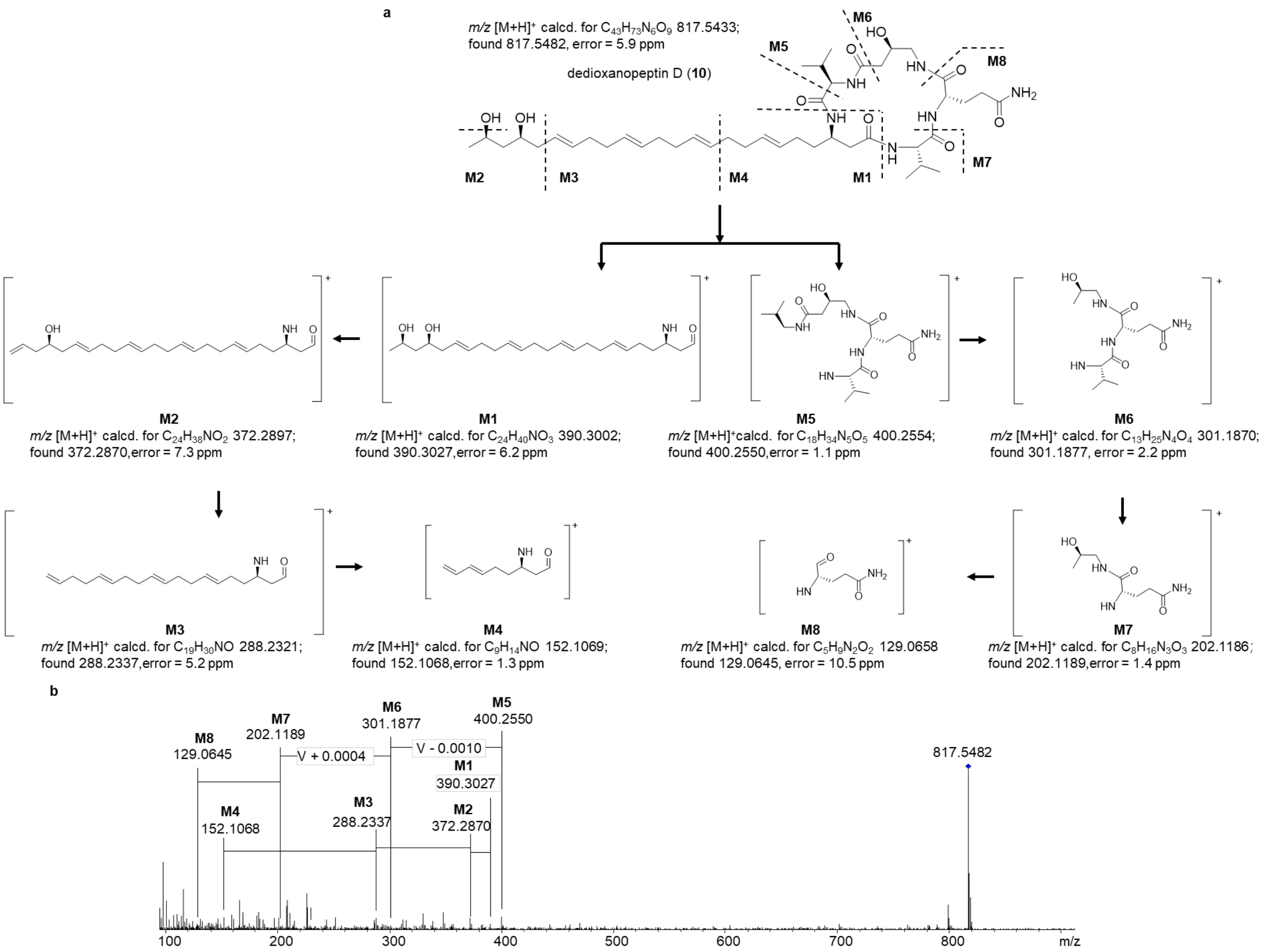

Supplementary Figure 17 | MS/MS fragmentation patterns of dedioxanopeptin D (**10**).

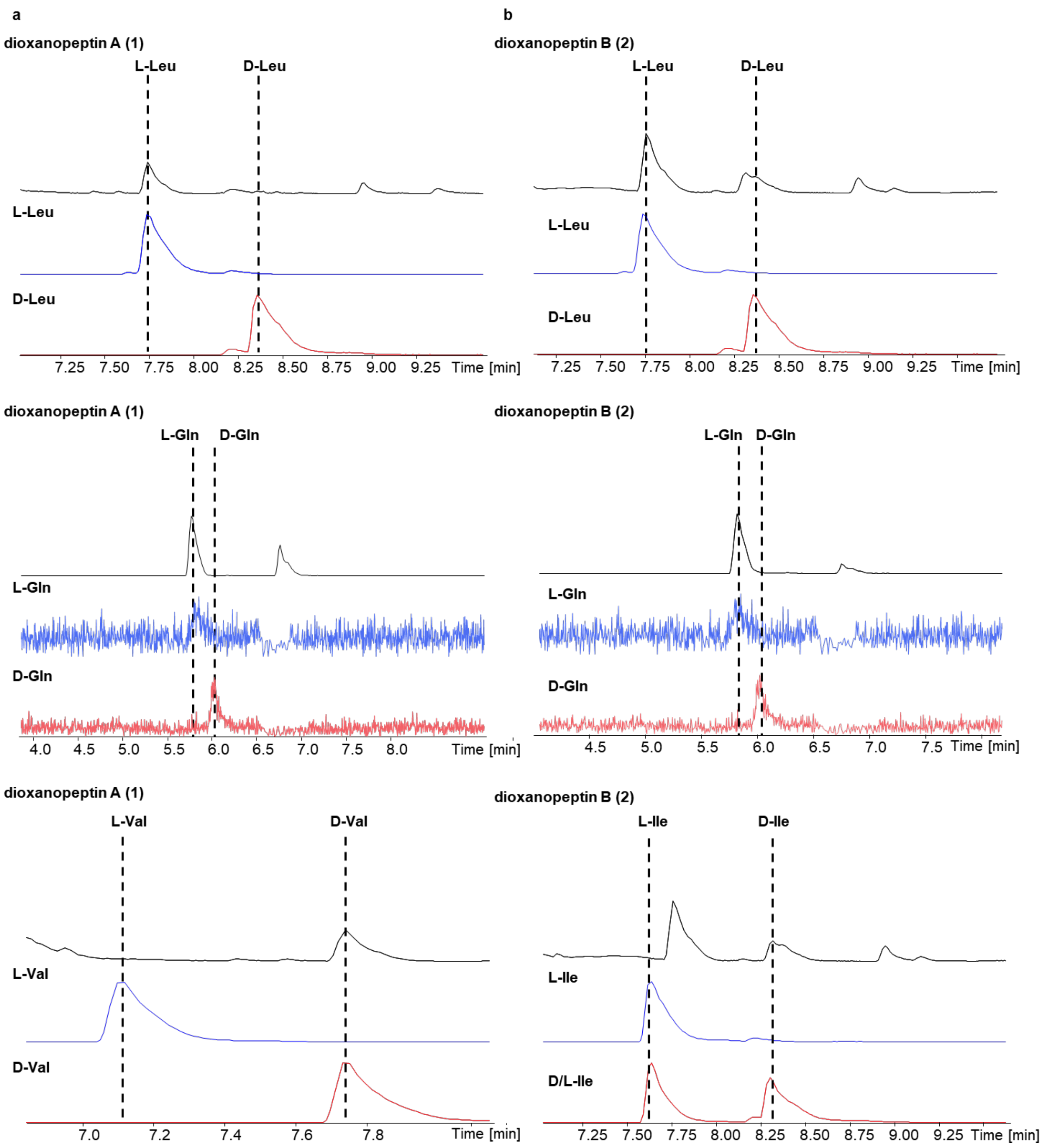

Supplementary Figure 18 | Absolute configuration determination of dioxanopeptins A (**1**) and B (**2**) by Marfey's method.

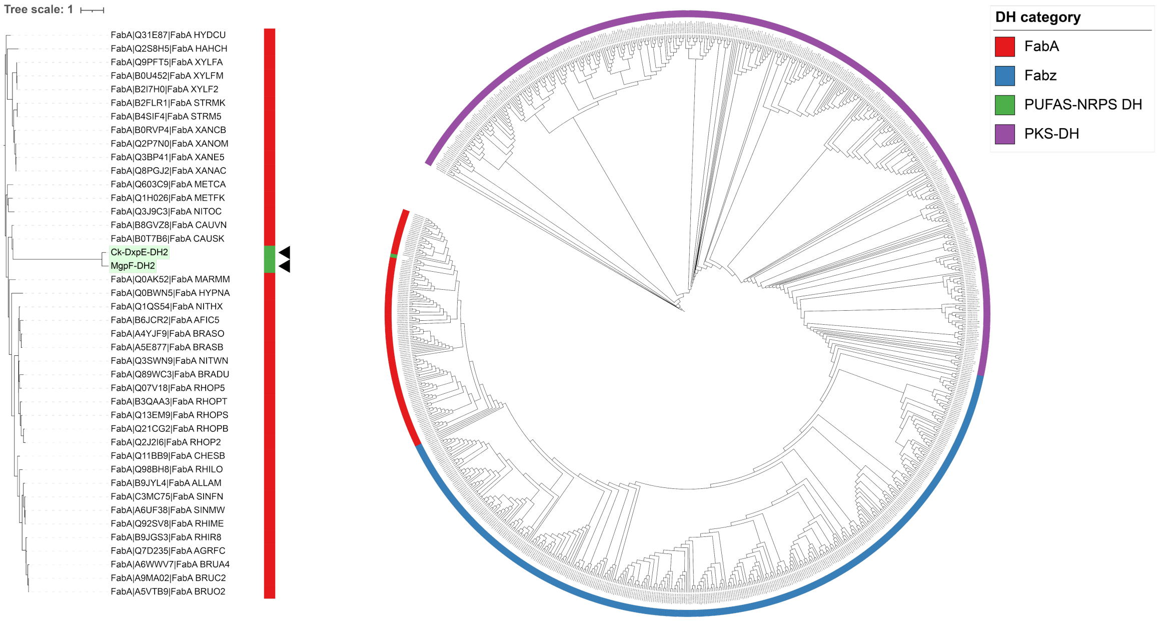

Supplementary Figure 19 | Phylogenetic analysis of FabA, FabZ, PKS DH domains, Ck-DxpE DH_2, and MgpF DH_2. FabA family, β-hydroxyacyl-ACP dehydratase/isomerases from type II fatty acid biosynthesis (dehydration to *trans*-2-enoyl-ACP and, on C10 substrates, isomerization to *cis*-3-enoyl-ACP). FabZ family, β-hydroxyacyl-ACP dehydratases from type II fatty acid elongation (dehydration to *trans*-2-enoyl-ACP without the FabA-type isomerization). Type I PKS DH domains that dehydrate β-hydroxyacyl thioesters to predominantly *trans* α,β-unsaturated enoyl intermediates. PUFAS DH domains from the *mgp* (megapolipeptin) PUFAS–NRPS BGC. The Ck-DxpE DH_2 (*trans*-2 dehydratase) clusters with the FabA branch together with the MgpF DH_2.

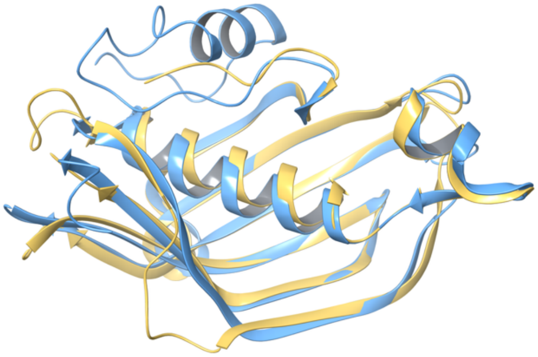

Supplementary Figure 20 | Structural comparison of the Ck-DxpE FabA-like region (yellow) with *Escherichia coli* FabA (PDB 1MKB, blue). antiSMASH annotated a FabA-like region within Ck-DxpE[4]. We predicted the structure of this segment using AlphaFold2 (v2.3.2; monomer_casp14 model)[41] and assessed its similarity to the canonical *E. coli* FabA structure (PDB 1MKB, chain A)[42] using Foldseek[43]. The alignment spanned 146 residues, covering 98.6% of the Ck-DxpE FabA-like segment and 81.3% of 1MKB_A. Despite only 29.4% sequence identity, it yielded a high TM-score (TM-score = 0.93; qTM = 0.87; tTM = 0.76) and a low backbone RMSD (2.0 Å), with a mean lDDT of 0.77 across the aligned region (E-value 2.9×10^{-13}; bit score 433). These metrics indicate that the Ck-DxpE FabA-like region adopts a highly similar fold to 1MKB_A and is consistent with a FabA-type β-hydroxyacyl-ACP dehydratase/isomerase homolog.

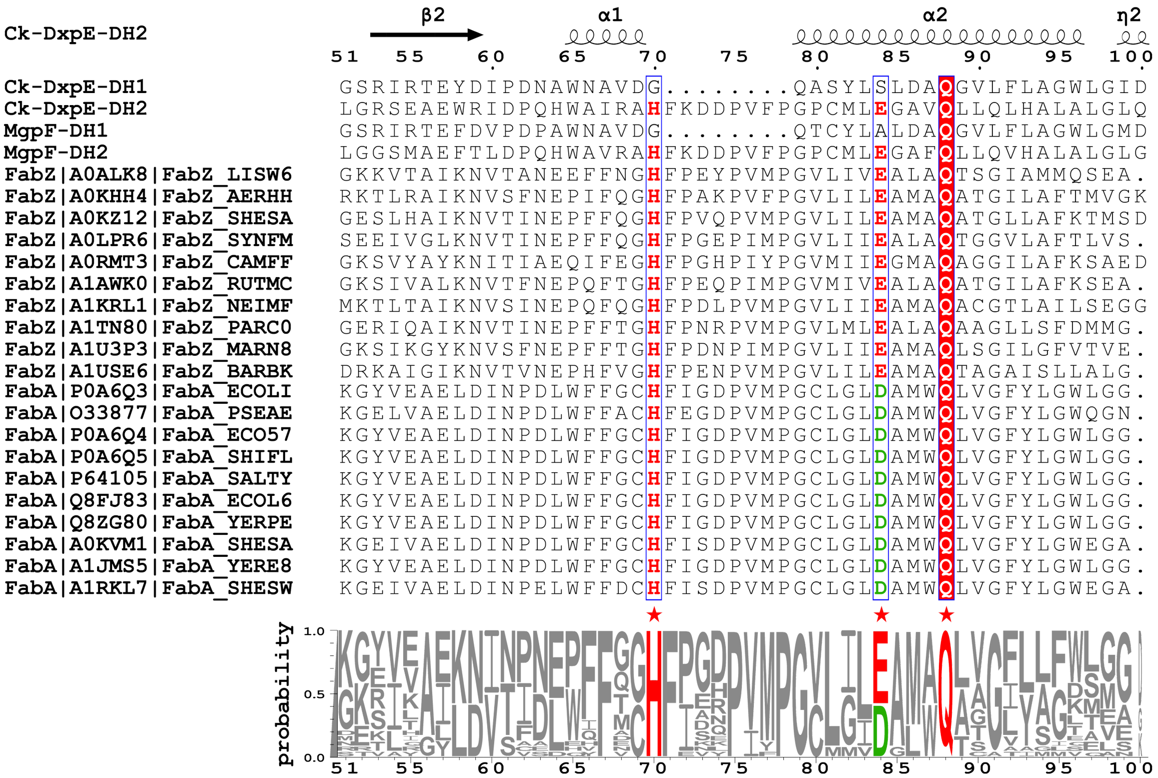

Supplementary Figure 21 | Multiple sequence alignment of the key catalytic residues in PUFA-associated hotdog-fold dehydratase domains. This composite figure combines a multiple sequence alignment and the corresponding sequence logo for the same region. The upper panel shows an alignment of CK-DxpE DHs and MgpF DHs together with UniProt-reviewed FabA and FabZ reference sequences. The lower panel shows the sequence logo derived from the identical alignment window, reporting site-wise information content and conservation. Blue boxes indicate the catalytic histidine position corresponding to His70 and the partner acidic residue position corresponding to Asp84 in FabA or Glu84 in FabZ. Red highlights denote key conserved positions anchoring the catalytic core and adjacent conserved blocks, whereas green highlights denote FabA specific positions used to discriminate FabA-type from FabZ-type domains. Red asterisks indicate the positions examined in this study.

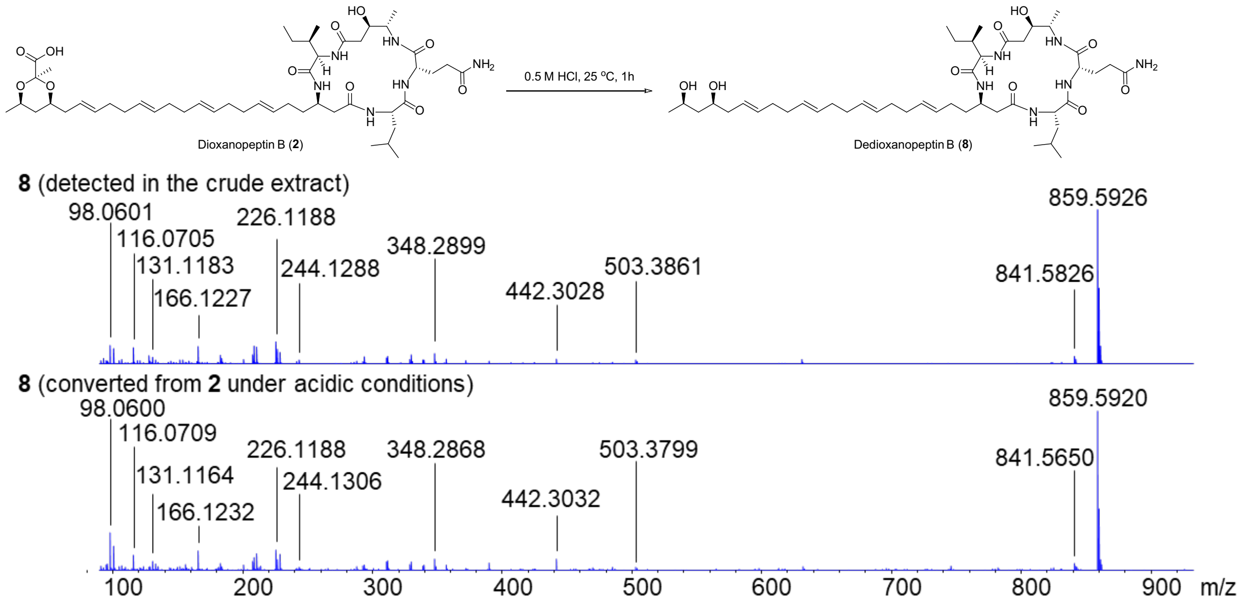

Supplementary Figure 22 | Chemical conversion of dioxanopeptin B (**2**) to dedioxanopeptin B (**8**) and MS/MS comparison of **8** obtained by conversion with **8** detected in the crude extract.

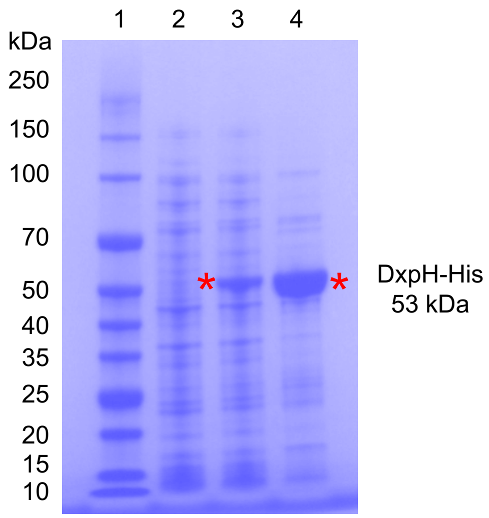

Supplementary Figure 23 | SDS–PAGE analysis of recombinant Ck-DxpH. The expected size of the C-terminal His_6-tagged Ck-DxpH (53 kDa) is indicated by an asterisk. Lane 1, protein marker. Lane 2, soluble fraction (supernatant after cell lysis) from IPTG-induced cells harboring the empty vector. Lane 3, soluble fraction from (supernatant after cell lysis) IPTG-induced cells expressing *dxpH*. Lane 4, purified DxpH (C-terminal His_6-tagged).

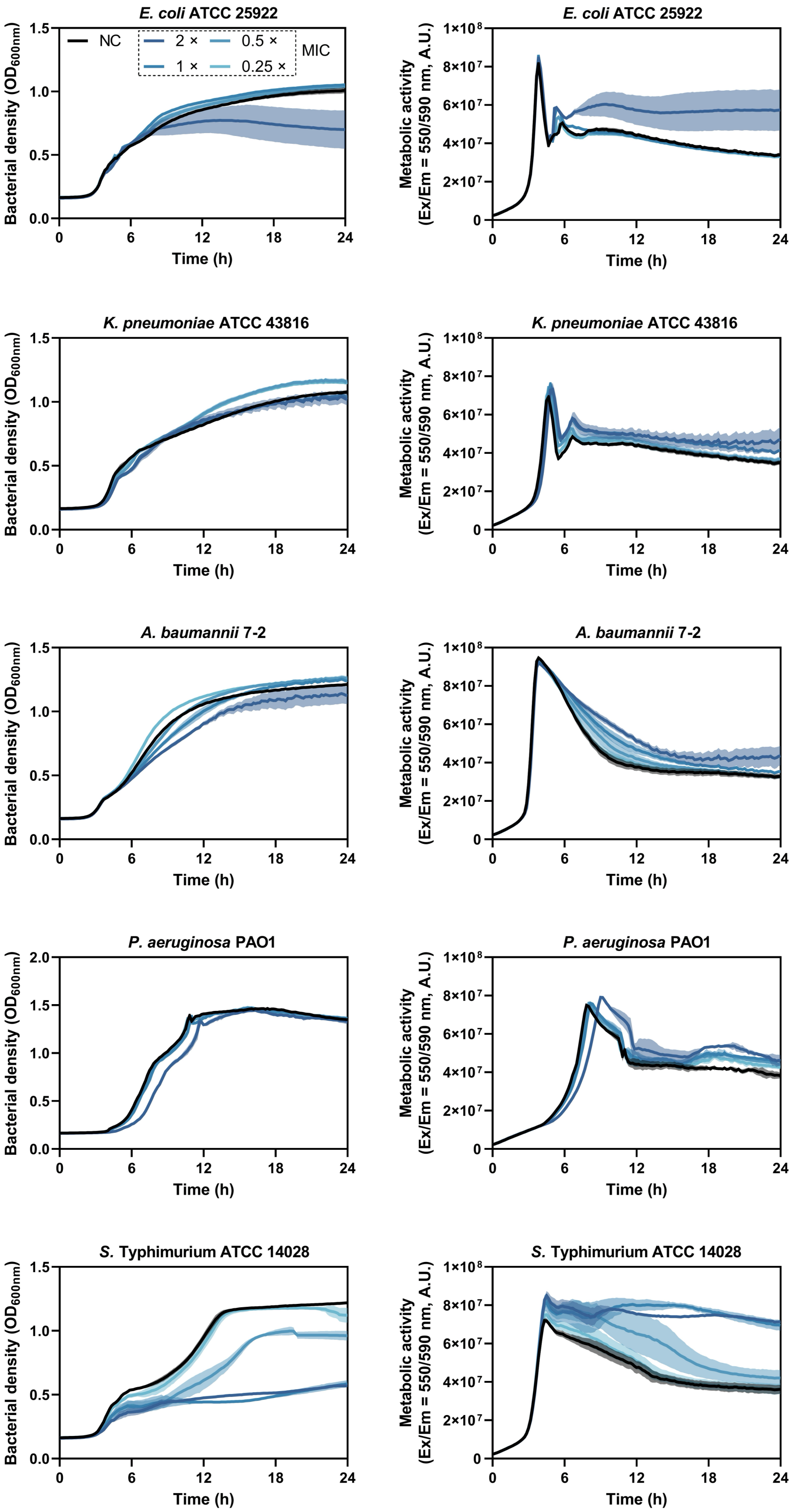

Supplementary Figure 24 | Growth dynamics of gram-negative bacteria under the concentration of 0.25×, 0.5×, 1×, and 2× MICs of dioxanopeptin A (**1**).

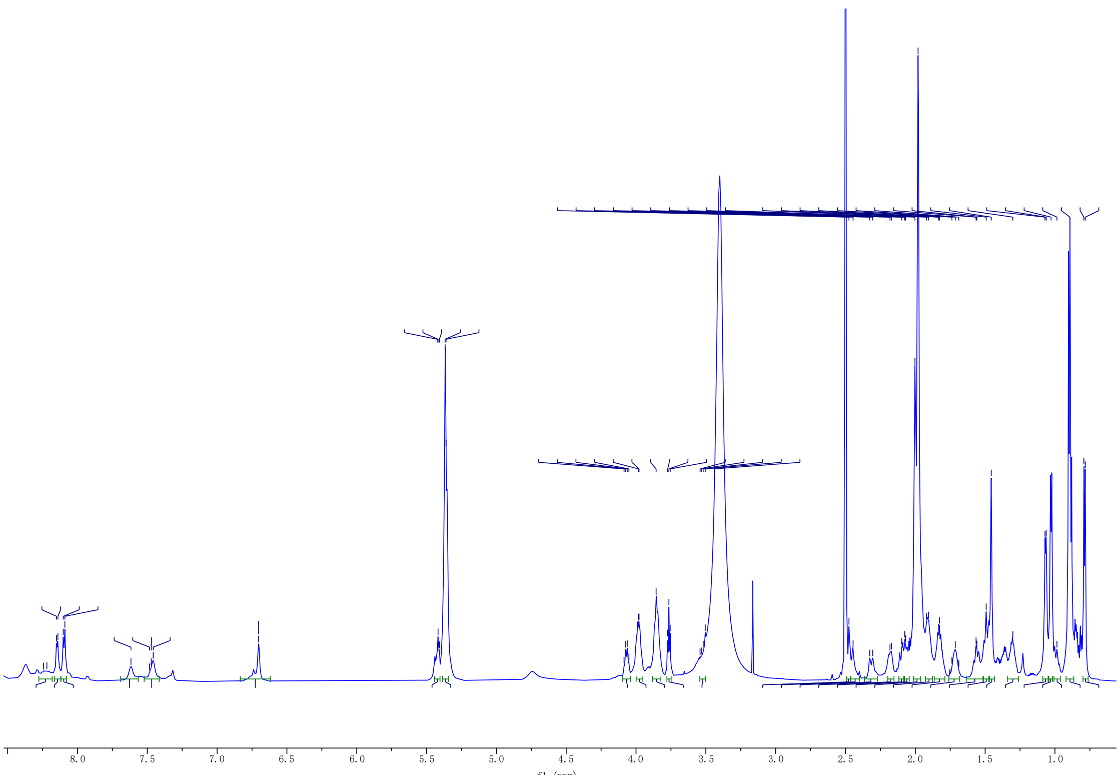

Supplementary Figure 25 | ^{1}H NMR spectrum of dioxanopeptin A (**1**) in DMSO-d_6.

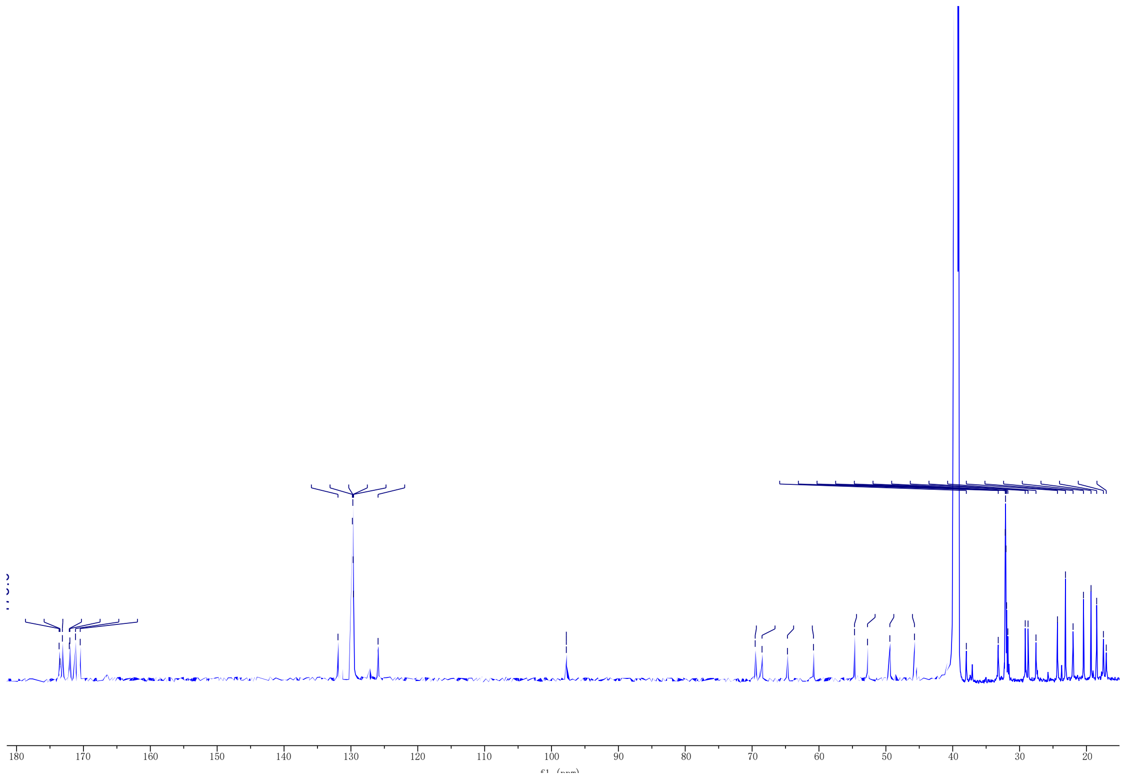

Supplementary Figure 26 | ^{13}C NMR spectrum of dioxanopeptin A (**1**) in DMSO-d_6.

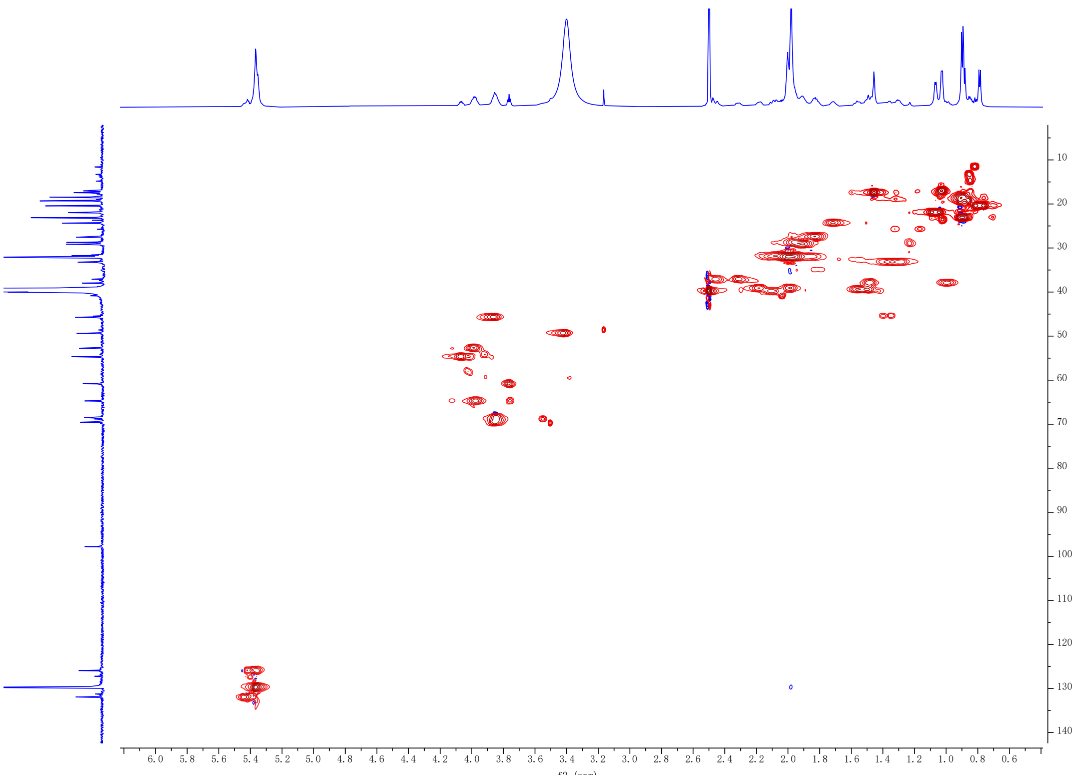

Supplementary Figure 27 | HSQC spectrum of dioxanopeptin A (**1**) in DMSO-d_6.

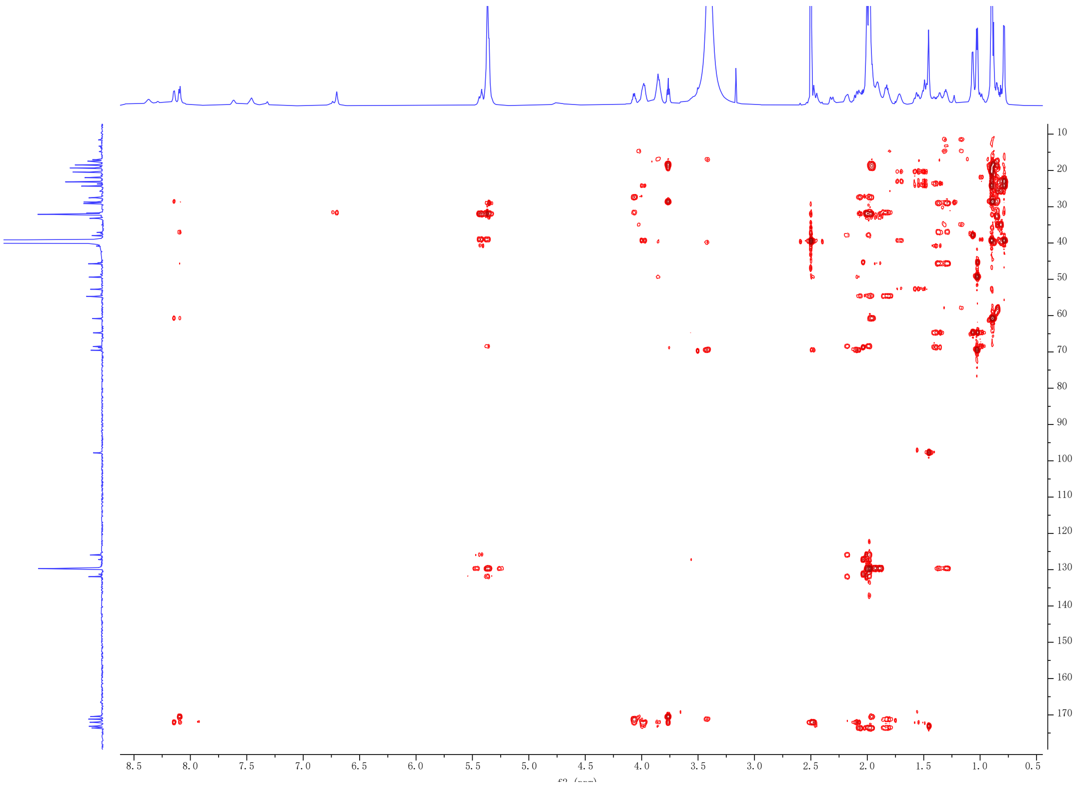

Supplementary Figure 28 | HMBC spectrum of dioxanopeptin A (**1**) in DMSO-d_6.

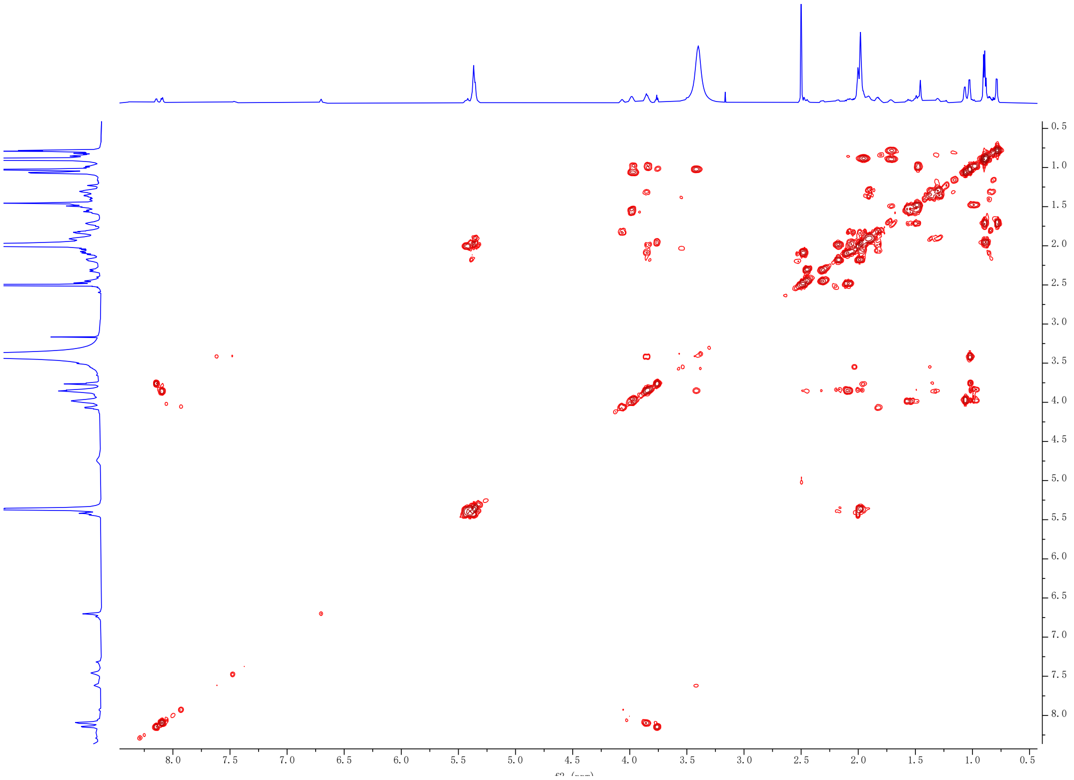

Supplementary Figure 29 | ^{1}H-^{1}H COSY spectrum of dioxanopeptin A (**1**) in DMSO-d_6.

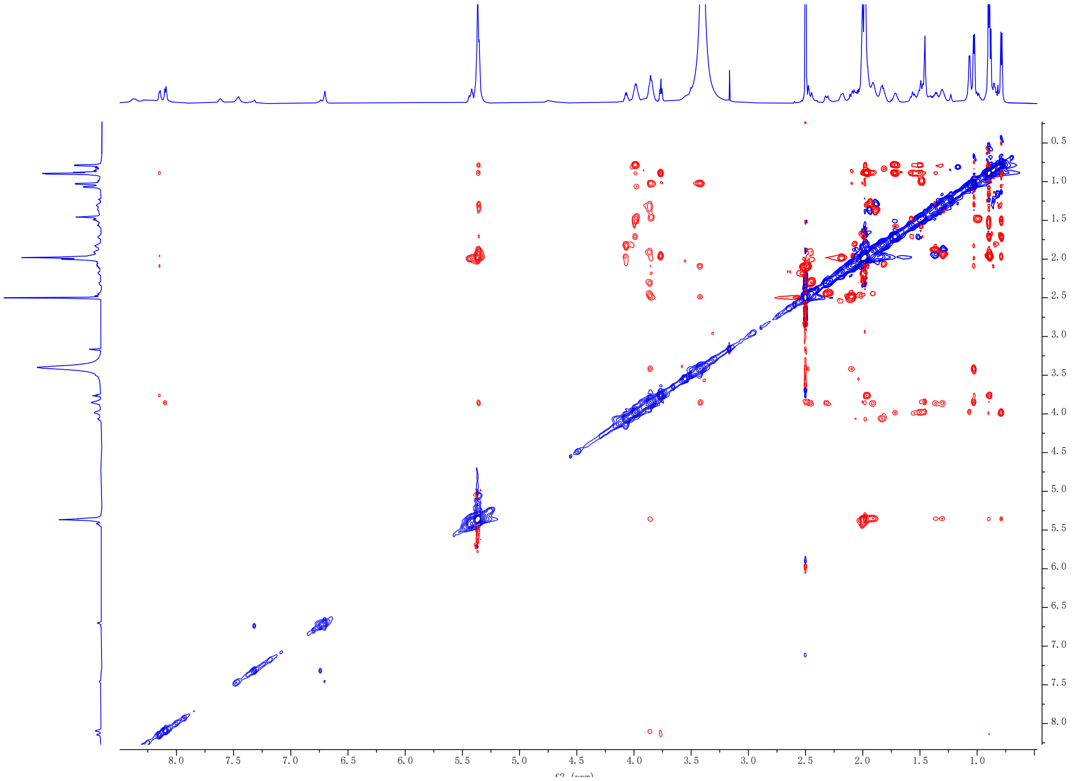

Supplementary Figure 30 | ROESY spectrum of dioxanopeptin A (**1**) in DMSO-d_6.

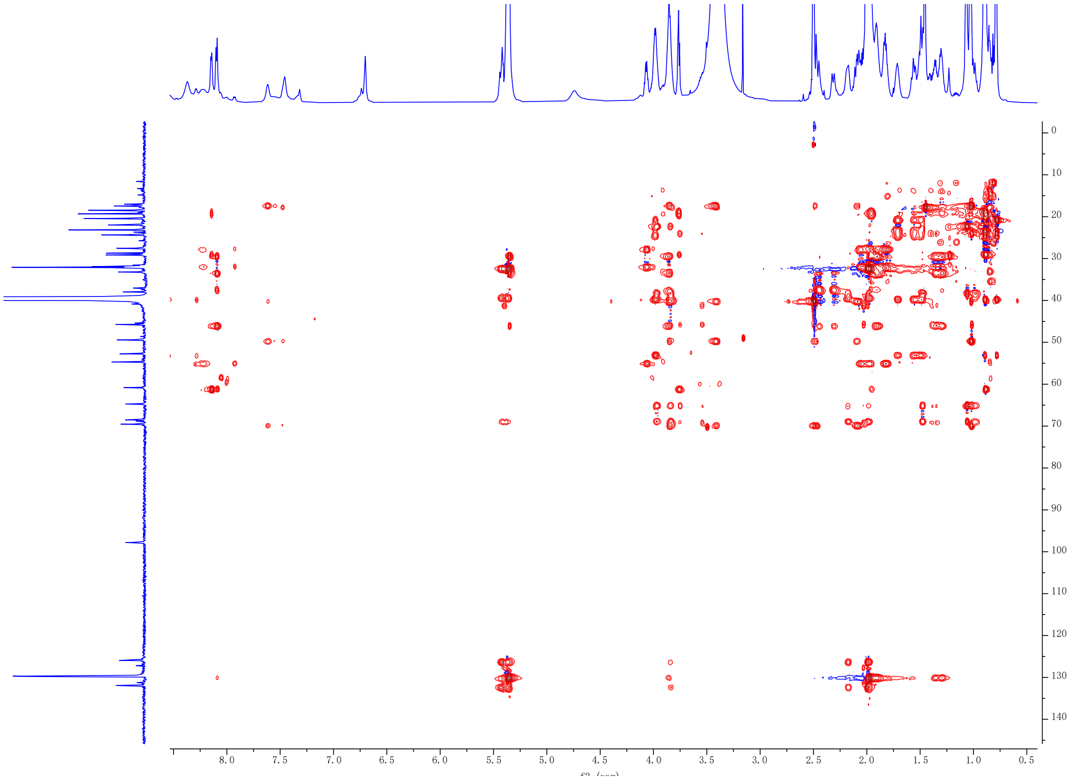

Supplementary Figure 31 | HSQC-TOCSY spectrum of dioxanopeptin A (**1**) in DMSO-d_6.

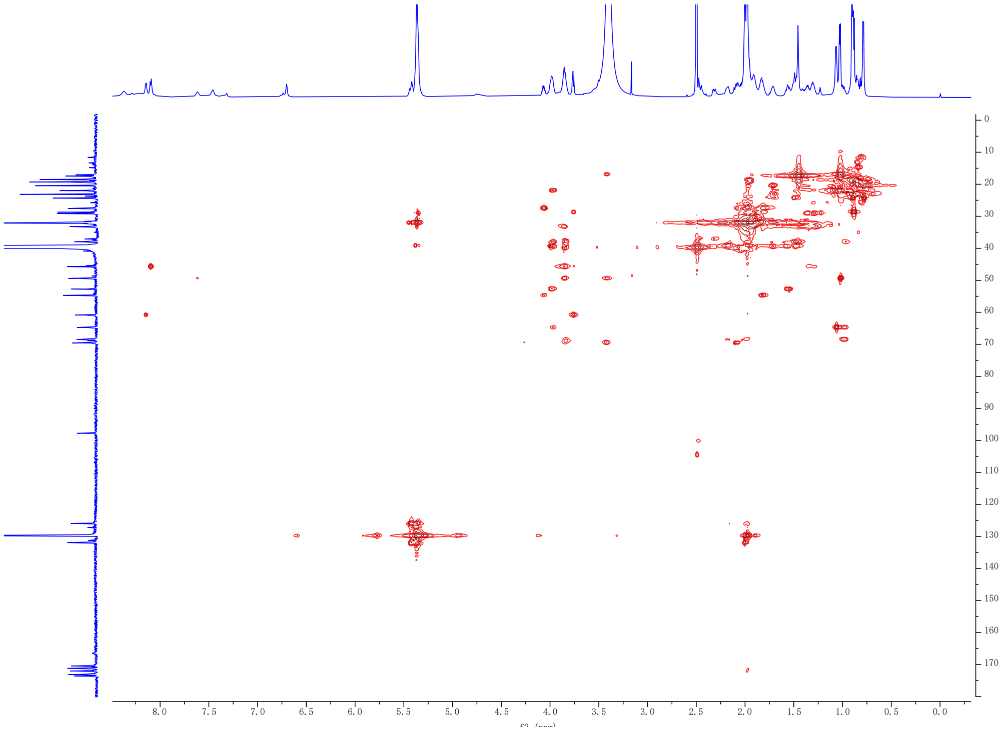

Supplementary Figure 32 | HMQC-COSY spectrum of dioxanopeptin A (**1**) in DMSO-d_6.

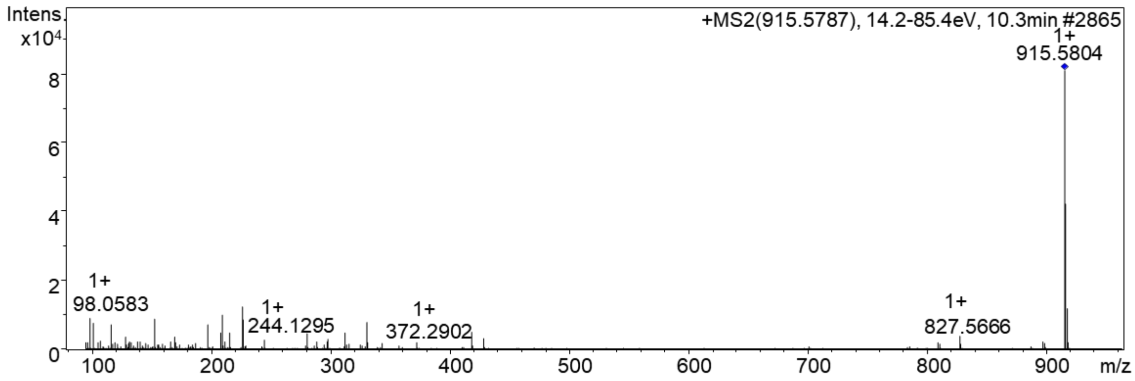

Supplementary Figure 33 | HR-ESI-MS of dioxanopeptin A (**1**).

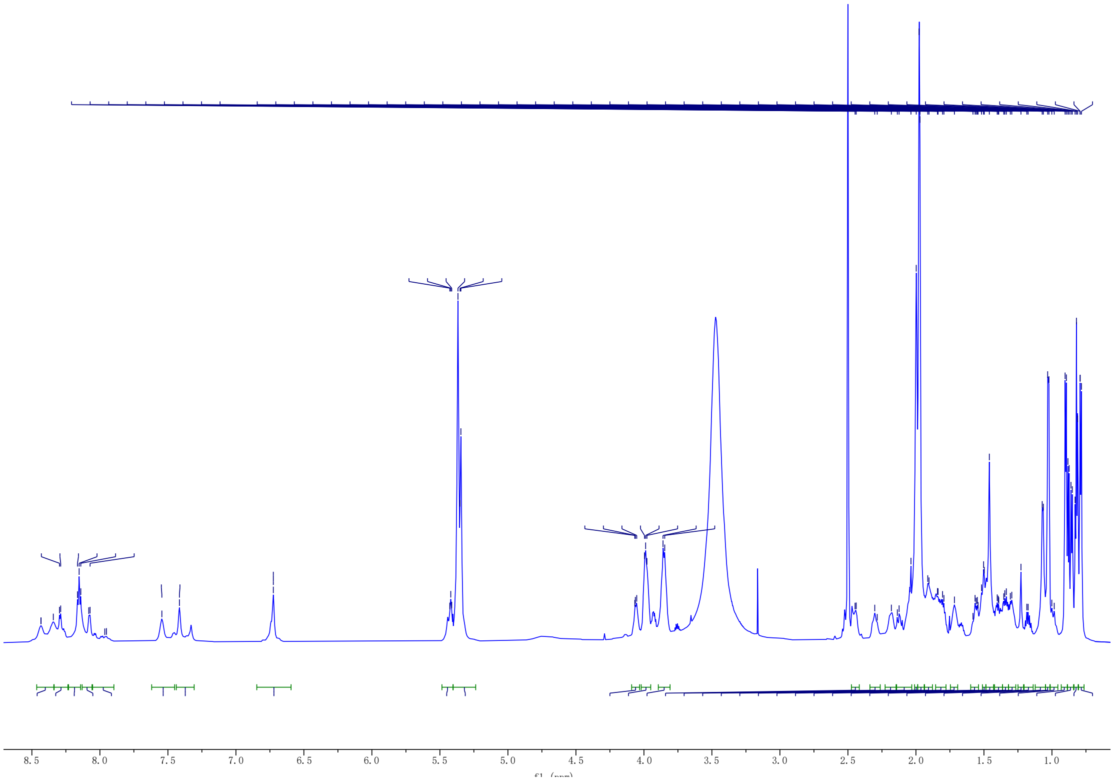

Supplementary Figure 34 | ^{1}H NMR spectrum of dioxanopeptin B (**2**) in DMSO-d_6.

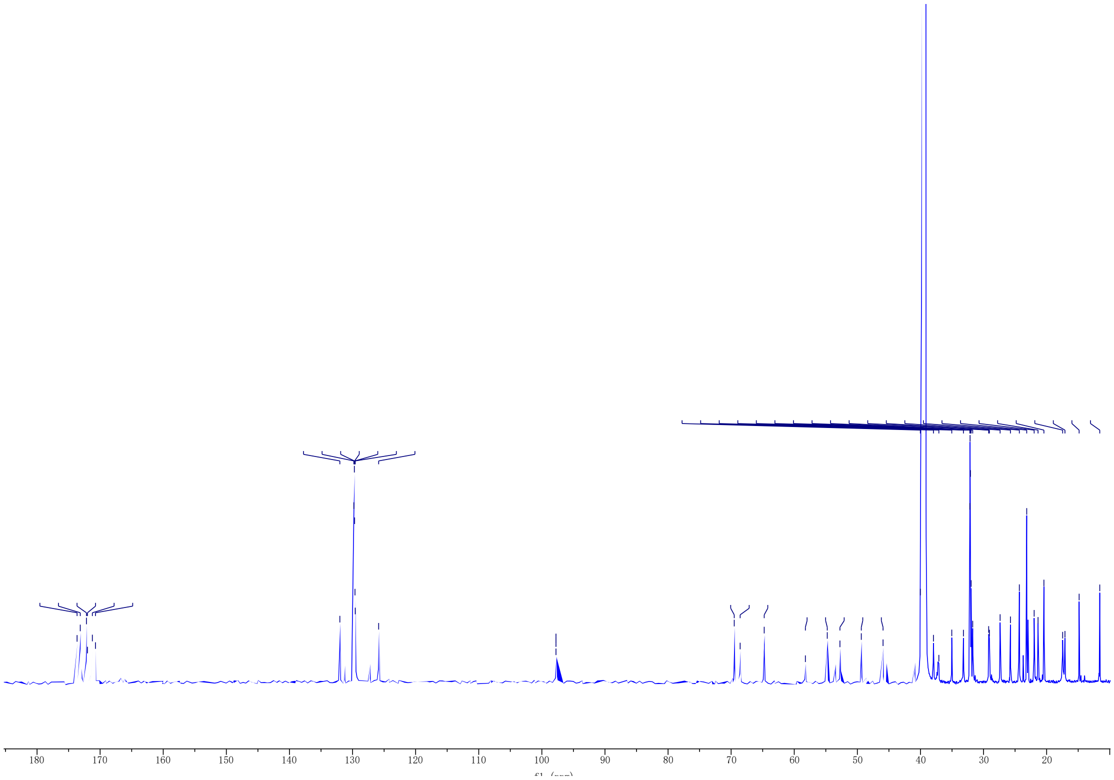

Supplementary Figure 35 | ^{13}C NMR spectrum of dioxanopeptin B (**2**) in DMSO-*d*$_6$.

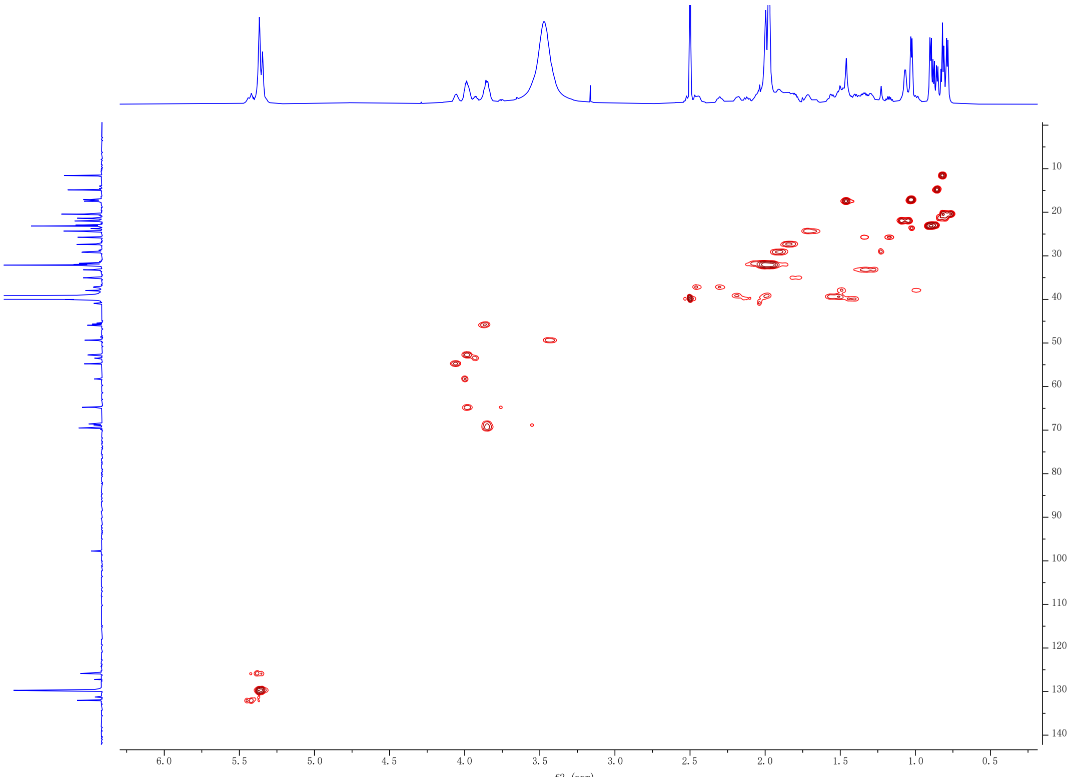

Supplementary Figure 36 | HSQC spectrum of dioxanopeptin B (**2**) in DMSO-*d*$_6$.

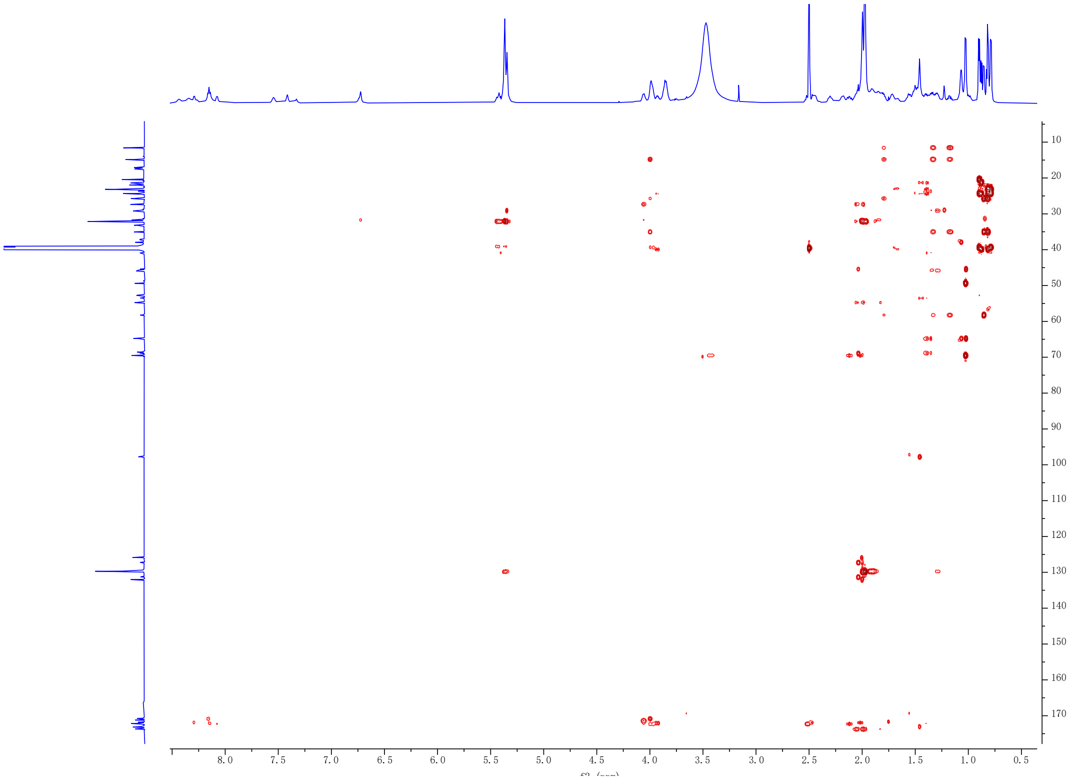

Supplementary Figure 37 | HMBC spectrum of dioxanopeptin B (**2**) in DMSO-d_6.

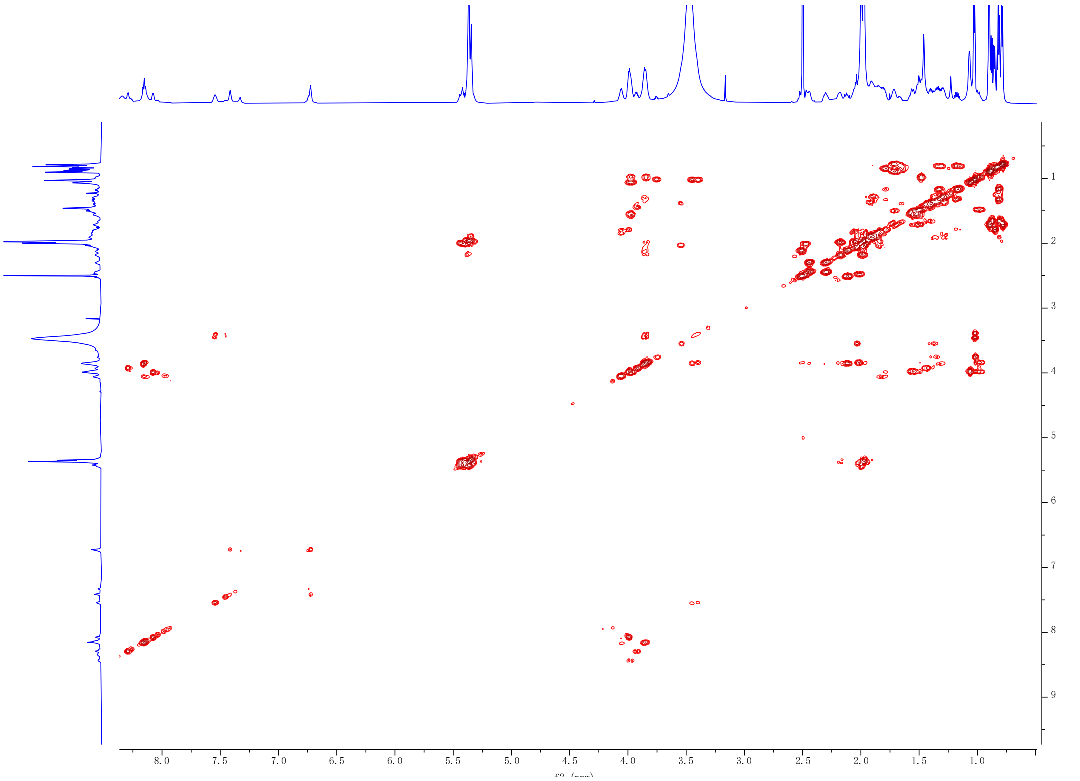

Supplementary Figure 38 | ^{1}H-^{1}H COSY spectrum of dioxanopeptin B (**2**) in DMSO-d_6.

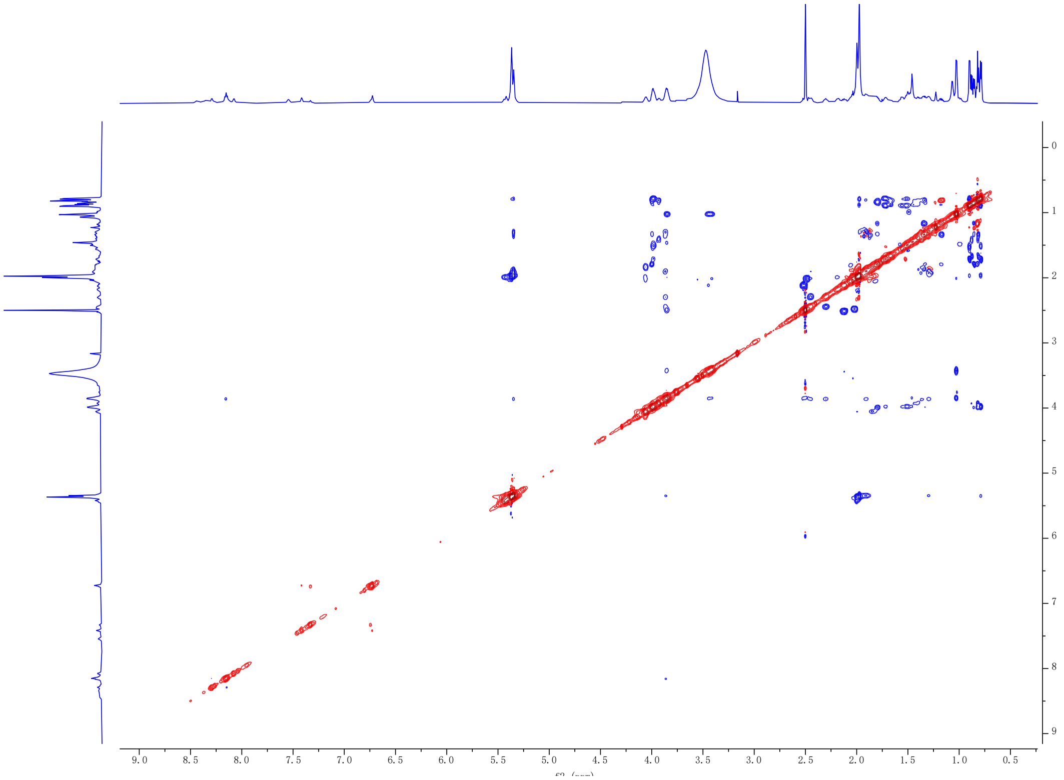

Supplementary Figure 39 | ROESY spectrum of dioxanopeptin B (**2**) in DMSO-d_6.

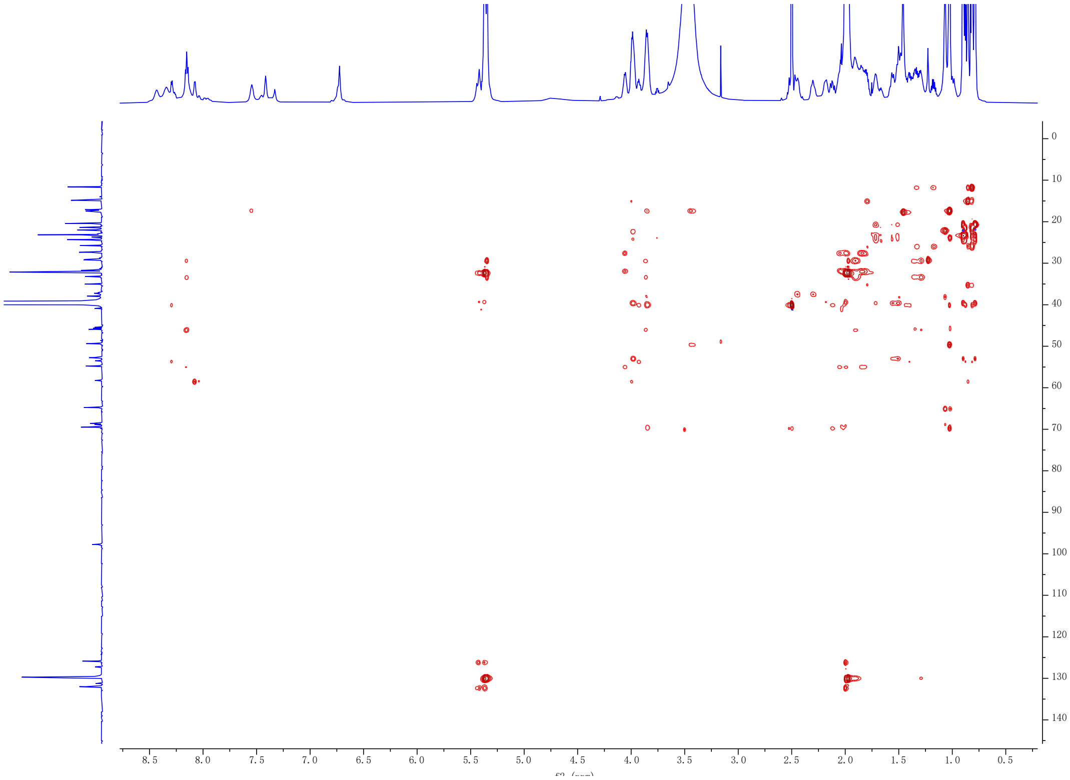

Supplementary Figure 40 | HSQC-TOCSY spectrum of dioxanopeptin B (**2**) in DMSO-d_6.

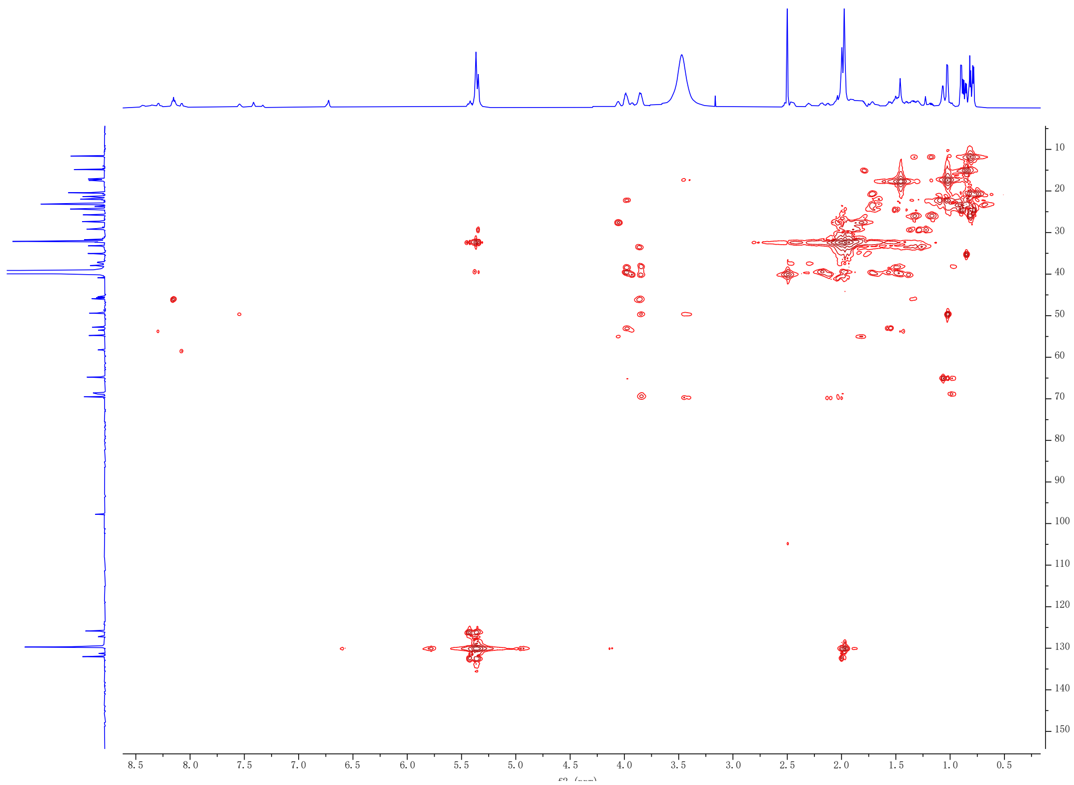

Supplementary Figure 41 | HMQC-COSY spectrum of dioxanopeptin B (**2**) in DMSO-d_6.

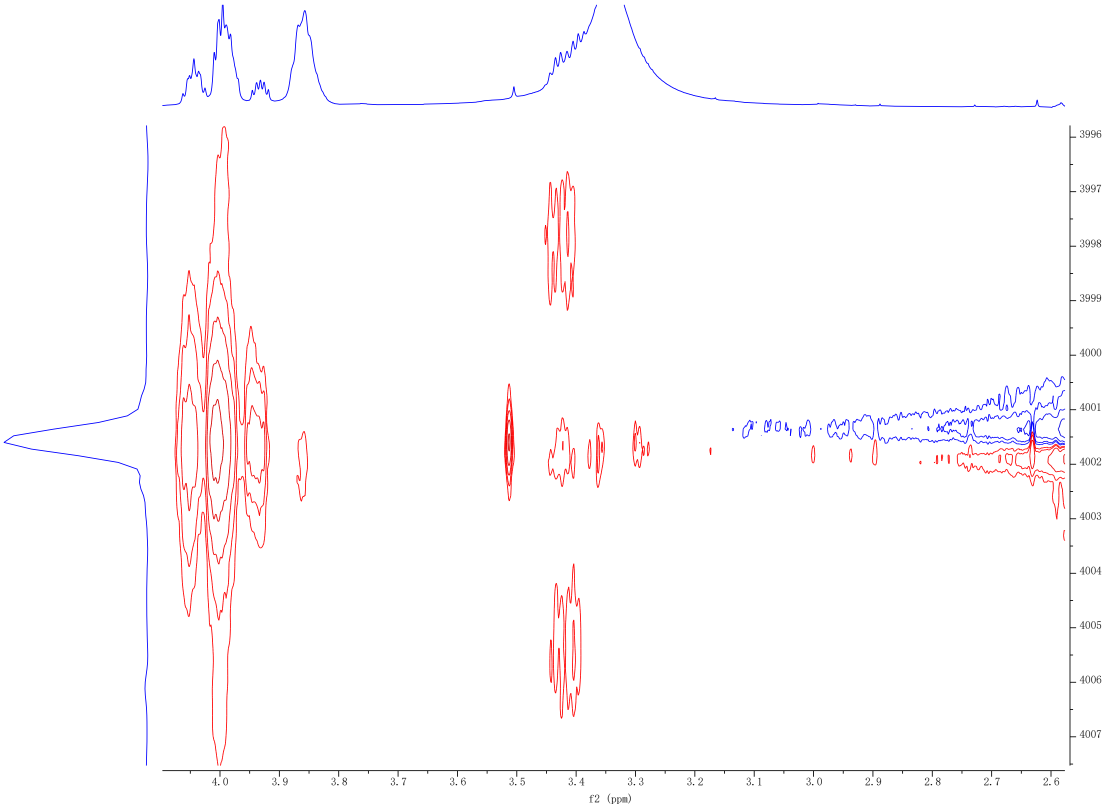

Supplementary Figure 42 | PSYCHEDELIC spectrum of dioxanopeptin B (**2**).

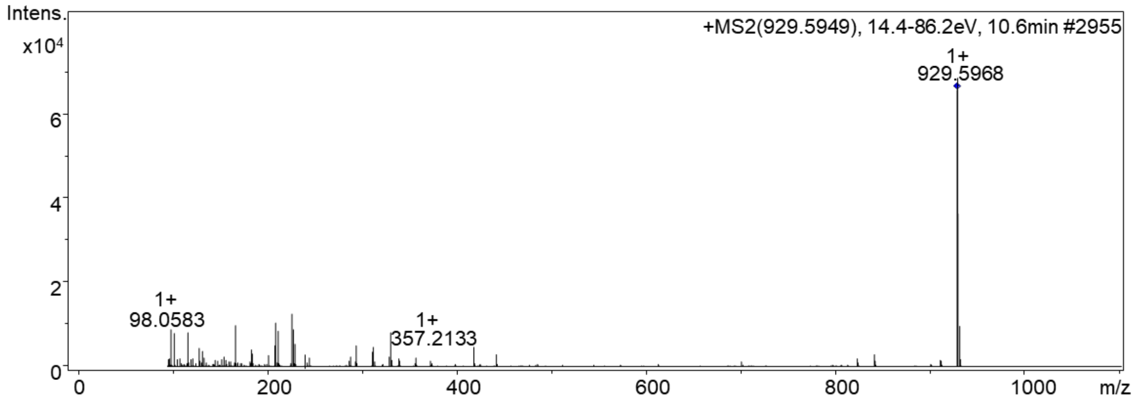

Supplementary Figure 43 | HR-ESI-MS of dioxanopeptin B (**2**).

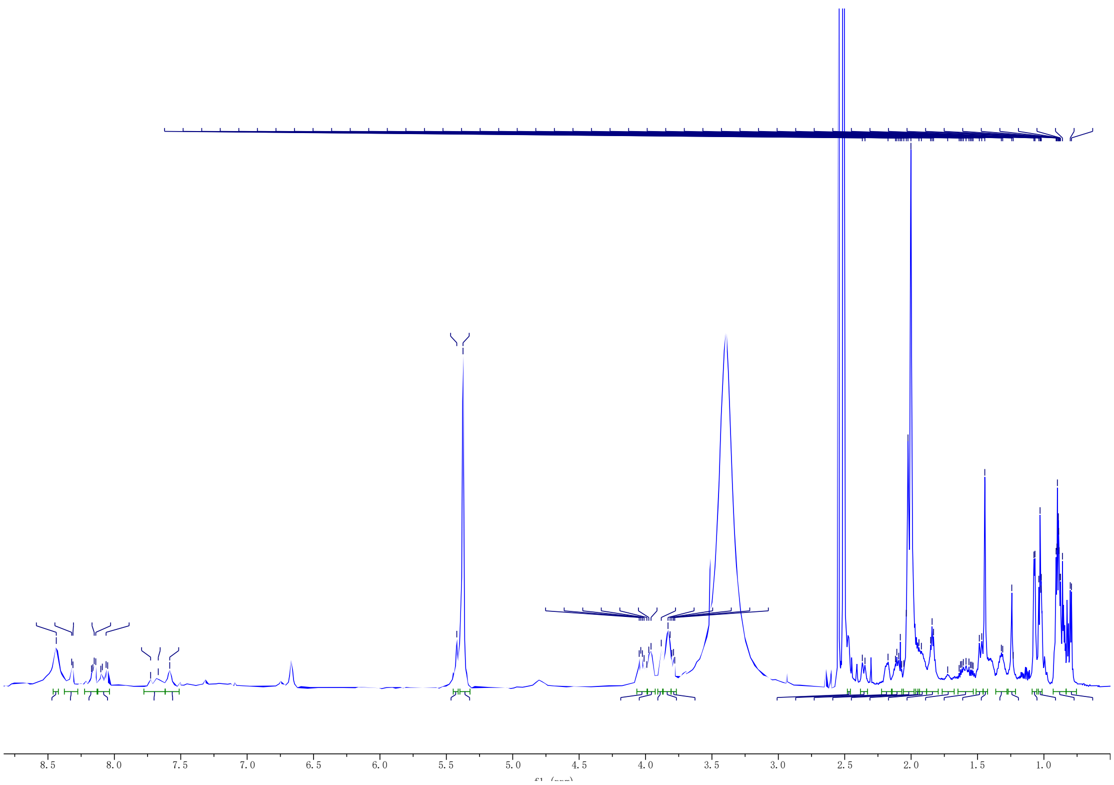

Supplementary Figure 44 | ^{1}H NMR spectrum of dioxanopeptin C (**3**) in DMSO-d_6.

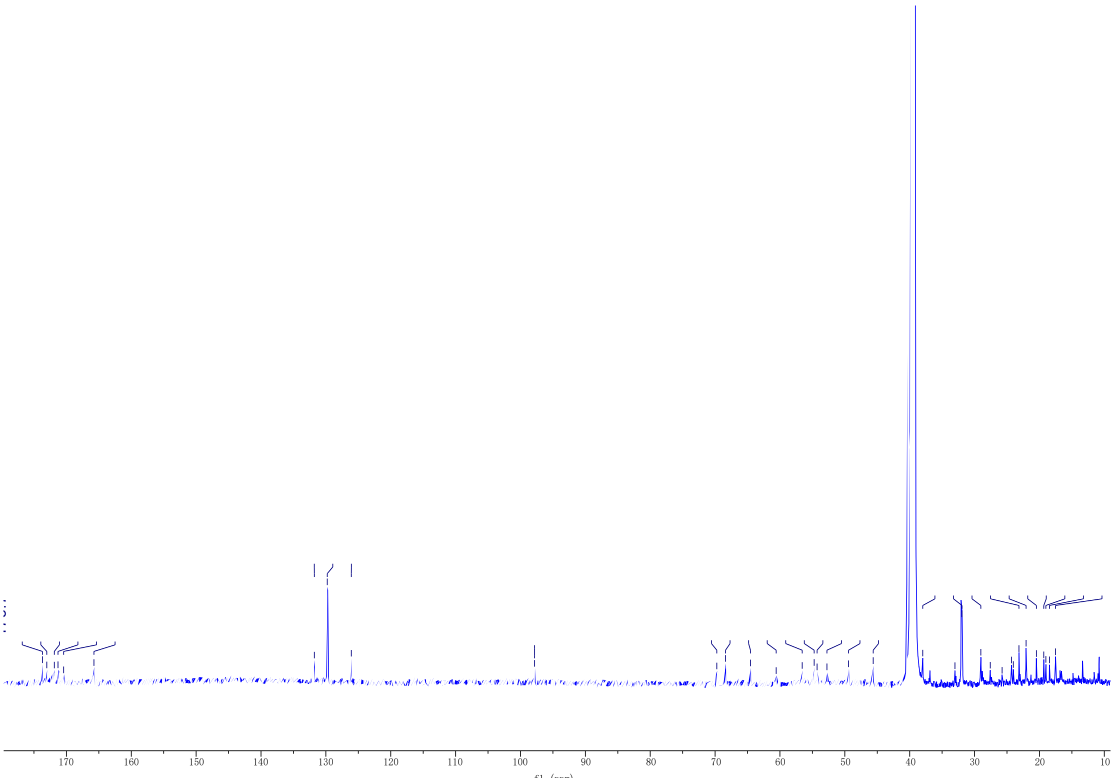

Supplementary Figure 45 | ^{13}C NMR spectrum of dioxanopeptin C (**3**) in DMSO-d_6.

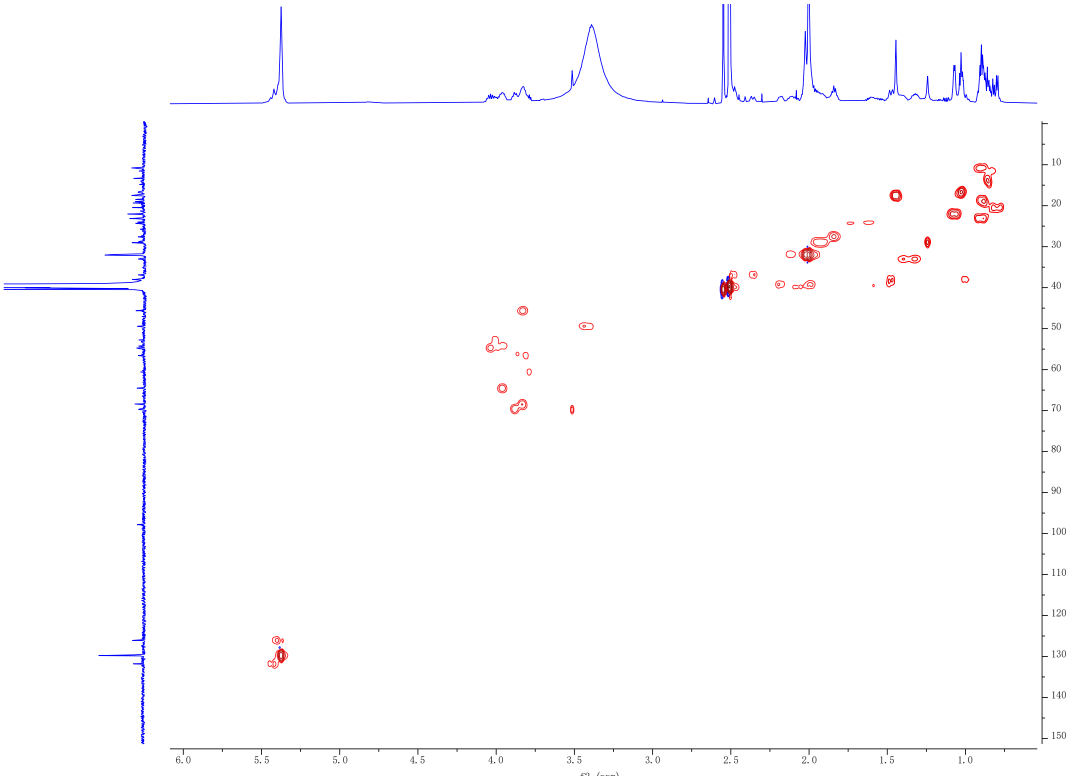

Supplementary Figure 46 | HSQC spectrum of dioxanopeptin C (**3**) in DMSO-d_6.

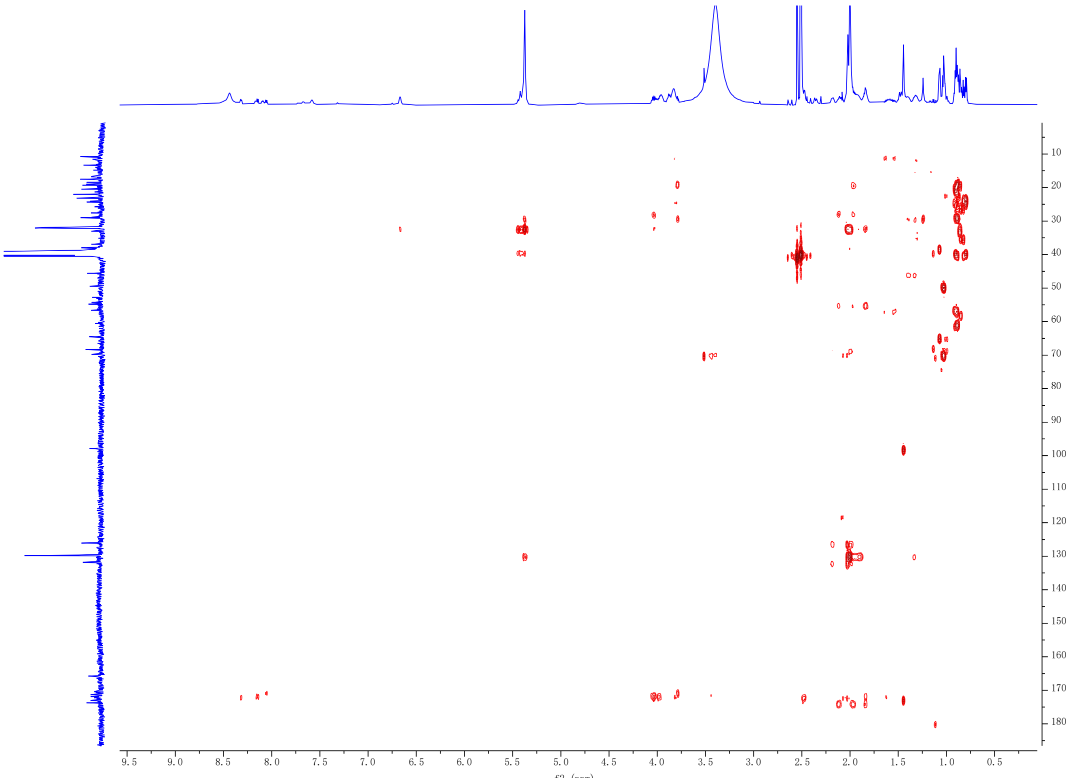

Supplementary Figure 47 | HMBC spectrum of dioxanopeptin C (**3**) in DMSO-d_6.

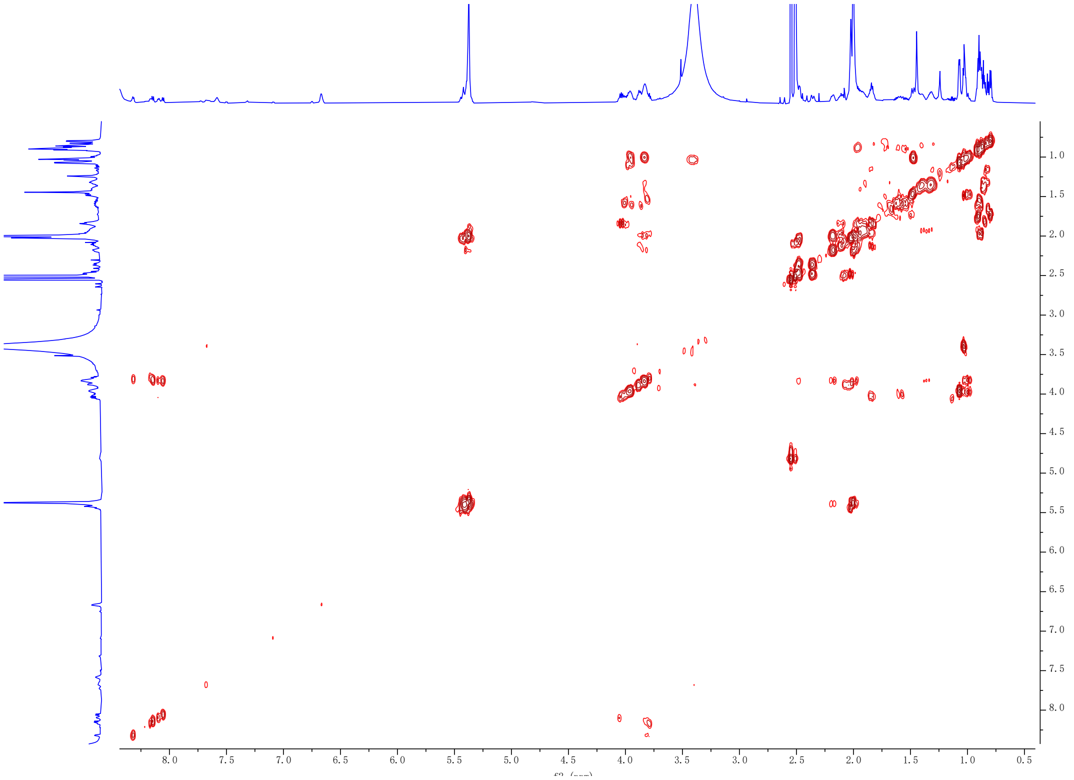

Supplementary Figure 48 | ^{1}H-^{1}H COSY spectrum of dioxanopeptin C (**3**) in DMSO-d_6.

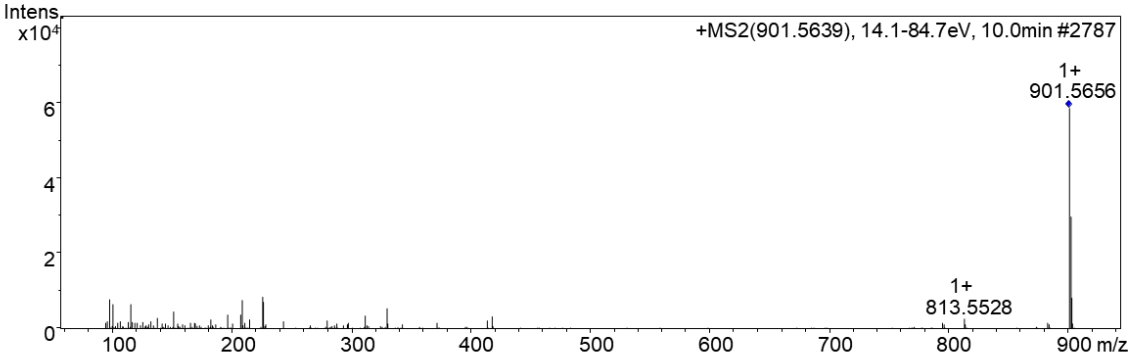

Supplementary Figure 49 | HR-ESI-MS of dioxanopeptin C (**3**).

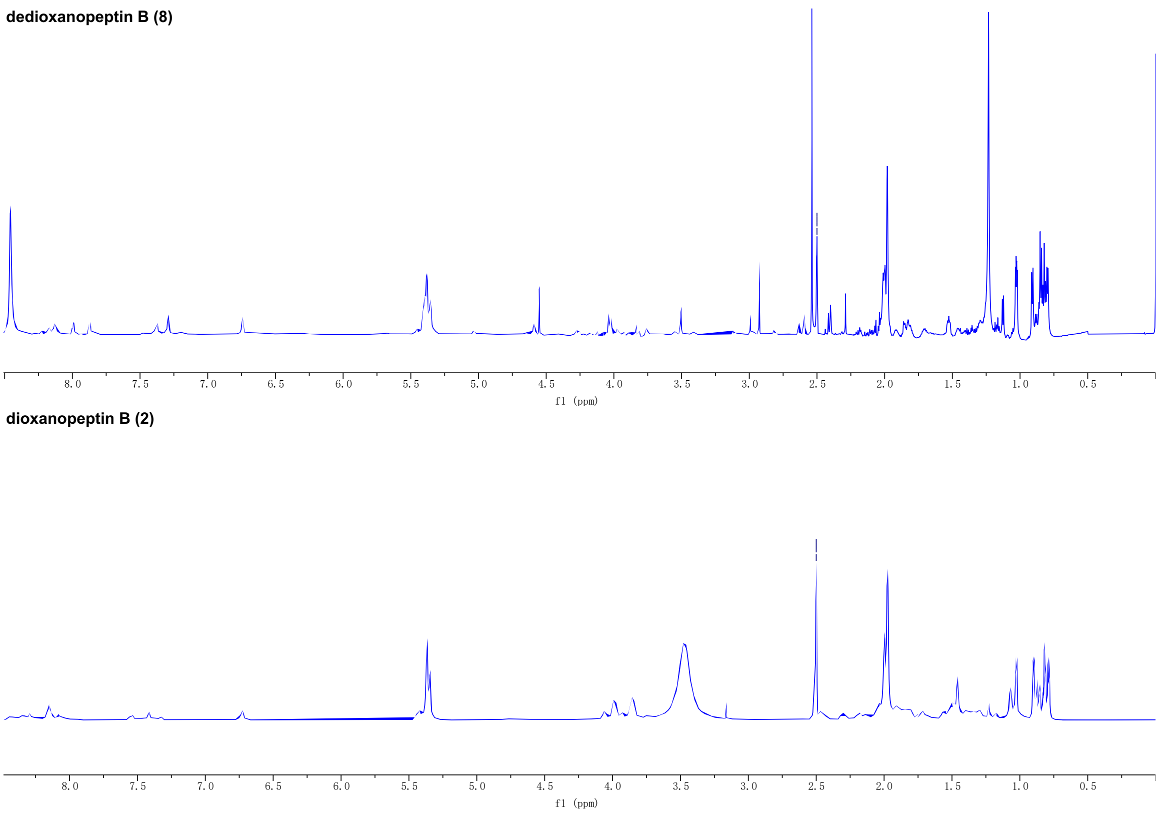

Supplementary Figure 50 | Comparison of the ^{1}H NMR spectra of the chemically converted dedioxanopeptin B (**8**) and the isolated dioxanopeptin B (**2**) in DMSO-d_6.

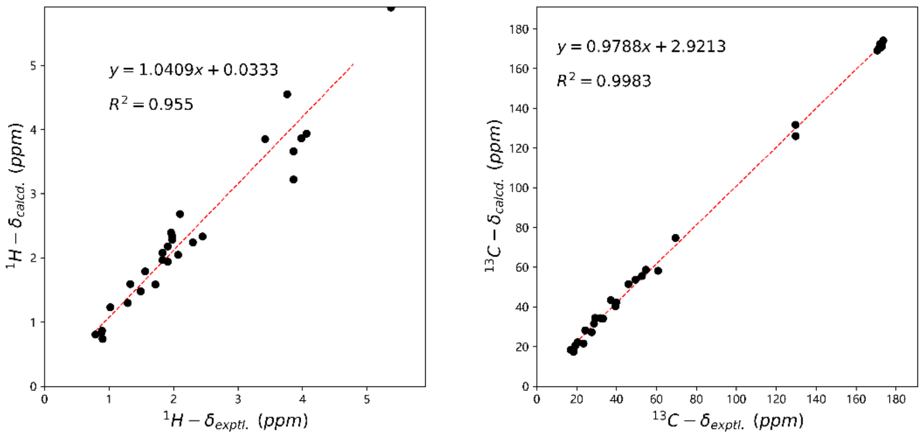

Supplementary Figure 51 | Linear regression analysis between experimental and calculated NMR chemical shifts of **1Ta**-25*R.*

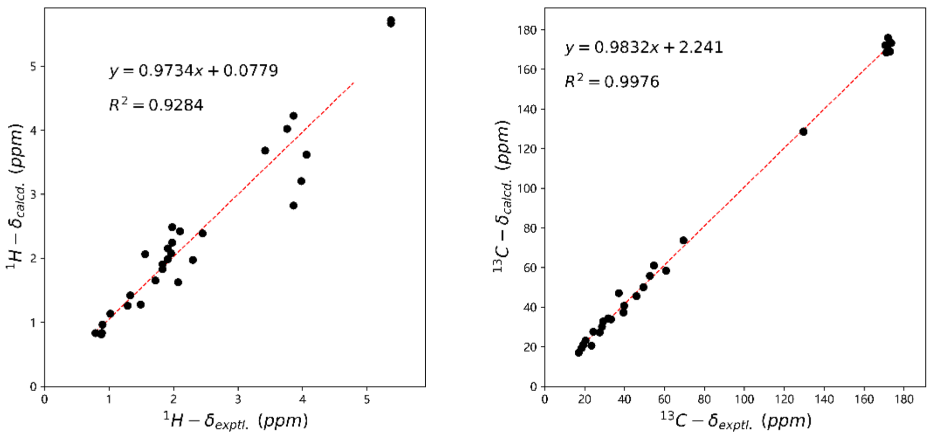

Supplementary Figure 52 | Linear regression analysis between experimental and calculated NMR chemical shifts of **1Tb**-25*S.*

References

1. S. Hayashi, Y. Satoh, T. Ujihara, Y. Takata and T. Dairi, *Sci Rep*, 2016, **6**, 35441.
2. C. N. Shulse and E. E. Allen, *PLoS One*, 2011, **6**, e20146.
3. C. Camacho, G. Coulouris, V. Avagyan, N. Ma, J. Papadopoulos, K. Bealer and T. L. Madden, *BMC Bioinf*, 2009, **10**, 421.
4. K. Blin, S. Shaw, L. Vader, J. Szenei, Zachary L. Reitz, Hannah E. Augustijn, José D. D. Cediel-Becerra, V. de Crécy-Lagard, Robert A. Koetsier, Sam E. Williams, P. Cruz-Morales, S. Wongwas, Alejandro E. Segurado Luchsinger, F. Biermann, A. Korenskaia, Mitja M. Zdouc, D. Meijer, Barbara R. Terlouw, Justin J. J. van der Hooft, N. Ziemert, Eric J. N. Helfrich, J. Masschelein, C. Corre, Marc G. Chevrette, Gilles P. van Wezel, Marnix H. Medema and T. Weber, *Nucleic Acids Res* 2025, **53**, W32–W38.
5. C. L. M. Gilchrist, T. J. Booth, B. van Wersch, L. van Grieken, M. H. Medema and Y.-H. Chooi, *Bioinform Adv*, 2021, **1**.
6. M. M. Zdouc, K. Blin, N. L. L. Louwen, J. Navarro, C. Loureiro, C. D. Bader, C. B. Bailey, L. Barra, T. J. Booth, K. A. J. Bozhuyuk, J. D. D. Cediel-Becerra, Z. Charlop-Powers, M. G. Chevrette, Y. H. Chooi, P. M. D'Agostino, T. de Rond, E. Del Pup, K. R. Duncan, W. Gu, N. Hanif, E. J. N. Helfrich, M. Jenner, Y. Katsuyama, A. Korenskaia, D. Krug, V. Libis, G. A. Lund, S. Mantri, K. D. Morgan, C. Owen, C. S. Phan, B. Philmus, Z. L. Reitz, S. L. Robinson, K. S. Singh, R. Teufel, Y. Tong, F. Tugizimana, D. Ulanova, J. M. Winter, C. Aguilar, D. Y. Akiyama, S. A. A. Al-Salihi, M. Alanjary, F. Alberti, G. Aleti, S. A. Alharthi, M. Y. A. Rojo, A. A. Arishi, H. E. Augustijn, N. E. Avalon, J. A. Avelar-Rivas, K. K. Axt, H. B. Barbieri, J. C. J. Barbosa, L. G. Barboza Segato, S. E. Barrett, M. Baunach, C. Beemelmanns, D. Beqaj, T. Berger, J. Bernaldo-Aguero, S. M. Bettenbuhl, V. A. Bielinski, F. Biermann, R. M. Borges, R. Borriss, M. Breitenbach, K. M. Bretscher, M. W. Brigham, L. Buedenbender, B. W. Bulcock, C. Cano-Prieto, J. Capela, V. J. Carrion, R. S. Carter, R. Castelo-Branco, G. Castro-Falcon, F. O. Chagas, E. Charria-Giron, A. A. Chaudhri, V. Chaudhry, H. Choi, Y. Choi, R. Choupannejad, J. Chromy, M. S. C. Donahey, J. Collemare, J. A. Connolly, K. E. Creamer, M. Crusemann, A. A. Cruz, A. Cumsille, J. F. Dallery, L. C. Damas-Ramos, T. Damiani, M. de Kruijff, B. D. Martin, G. D. Sala, J. Dillen, D. T. Doering, S. R. Dommaraju, S. Durusu, S. Egbert, M. Ellerhorst, B. Faussurier, A. Fetter, M. Feuermann, D. P. Fewer, J. Foldi, A. Frediansyah, E. A. Garza, A. Gavriilidou, A. Gentile, J. Gerke, H. Gerstmans, J. P. Gomez-Escribano, L. A. Gonzalez-Salazar, N. E. Grayson, C. Greco, J. E. G. Gomez, S. Guerra, S. G. Flores, A. Gurevich, K. Gutierrez-Garcia, L. Hart, K. Haslinger, B. He, T. Hebra, J. L. Hemmann, H. Hindra, L. Hoing, D. C. Holland, J. E. Holme, T. Horch, P. Hrab, J. Hu, T. H. Huynh, J. Y. Hwang, R. Iacovelli, D. Iftime, M. Iorio, S. Jayachandran, E. Jeong, J. Jing, J. J. Jung, Y. Kakumu, E. Kalkreuter, K. B. Kang, S. Kang, W. Kim, G. J. Kim, H. Kim, H. U. Kim, M. Klapper, R. A. Koetsier, C. Kollten, A. T. Kovacs, Y. Kriukova, N. Kubach,

A. M. Kunjapur, A. K. Kushnareva, A. Kust, J. Lamber, M. Larralde, N. J. Larsen, A. P. Launay, N. T. Le, S. Lebeer, B. T. Lee, K. Lee, K. L. Lev, S. M. Li, Y. X. Li, C. Licona-Cassani, A. Lien, J. Liu, J. A. V. Lopez, N. V. Machushynets, M. I. Macias, T. Mahmud, M. Maleckis, A. M. Martinez-Martinez, Y. Mast, M. F. Maximo, C. M. McBride, R. M. McLellan, K. M. Bhatt, C. Melkonian, A. Merrild, M. Metsa-Ketela, D. A. Mitchell, A. V. Muller, G. S. Nguyen, H. T. Nguyen, T. H. J. Niedermeyer, J. H. O'Hare, A. Ossowicki, B. O. Ostash, H. Otani, L. Padva, S. Paliyal, X. Pan, M. Panghal, D. S. Parade, J. Park, J. Parra, M. P. Rubio, H. T. Pham, S. J. Pidot, J. Piel, B. Pourmohsenin, M. Rakhmanov, S. Ramesh, M. H. Rasmussen, A. Rego, R. Reher, A. J. Rice, A. Rigolet, A. Romero-Otero, L. R. Rosas-Becerra, P. Y. Rosiles, A. Rutz, B. Ryu, L. A. Sahadeo, M. Saldanha, L. Salvi, E. Sanchez-Carvajal, C. Santos-Medellin, N. Sbaraini, S. M. Schoellhorn, C. Schumm, L. Sehnal, N. Selem, A. D. Shah, T. K. Shishido, S. Sieber, V. Silviani, G. Singh, H. Singh, N. Sokolova, E. C. Sonnenschein, M. Sosio, S. T. Sowa, K. Steffen, E. Stegmann, A. B. Streiff, A. Struder, F. Surup, T. Svenningsen, D. Sweeney, J. Szenei, A. Tagirdzhanov, B. Tan, M. J. Tarnowski, B. R. Terlouw, T. Rey, N. U. Thome, L. R. Torres Ortega, T. Torring, M. Trindade, A. W. Truman, M. Tvilum, D. W. Udwary, C. Ulbricht, L. Vader, G. P. van Wezel, M. Walmsley, R. Warnasinghe, H. G. Weddeling, A. N. M. Weir, K. Williams, S. E. Williams, T. E. Witte, S. M. W. Rocca, K. Yamada, D. Yang, D. Yang, J. Yu, Z. Zhou, N. Ziemert, L. Zimmer, A. Zimmermann, C. Zimmermann, J. J. J. van der Hooft, R. G. Linington, T. Weber and M. H. Medema, *Nucleic Acids Res*, 2025, **53**, D678–D690.

7. J. C. Navarro-Munoz, N. Selem-Mojica, M. W. Mullowney, S. A. Kautsar, J. H. Tryon, E. I. Parkinson, E. L. C. De Los Santos, M. Yeong, P. Cruz-Morales, S. Abubucker, A. Roeters, W. Lokhorst, A. Fernandez-Guerra, L. T. D. Cappelini, A. W. Goering, R. J. Thomson, W. W. Metcalf, N. L. Kelleher, F. Barona-Gomez and M. H. Medema, *Nat Chem Biol*, 2020, **16**, 60–68.

8. A. Draisma, C. Loureiro, N. L. L. Louwen, S. A. Kautsar, J. C. Navarro-Muñoz, D. T. Doering, N. J. Mouncey and M. H. Medema, *Nat. Commun.*, 2026, DOI: 10.1038/s41467-026-68733-5.

9. J. M. Gauglitz, K. A. West, W. Bittremieux, C. L. Williams, K. C. Weldon, M. Panitchpakdi, F. Di Ottavio, C. M. Aceves, E. Brown, N. C. Sikora, A. K. Jarmusch, C. Martino, A. Tripathi, M. J. Meehan, K. Dorrestein, J. P. Shaffer, R. Coras, F. Vargas, L. D. Goldasich, T. Schwartz, M. Bryant, G. Humphrey, A. J. Johnson, K. Spengler, P. Belda-Ferre, E. Diaz, D. McDonald, Q. Zhu, E. O. Elijah, M. Wang, C. Marotz, K. E. Sprecher, D. Vargas-Robles, D. Withrow, G. Ackermann, L. Herrera, B. J. Bradford, L. M. M. Marques, J. G. Amaral, R. M. Silva, F. P. Veras, T. M. Cunha, R. D. R. Oliveira, P. Louzada-Junior, R. H. Mills, P. K. Piotrowski, S. L. Servetas, S. M. Da Silva, C. M. Jones, N. J. Lin, K. A. Lippa, S. A. Jackson, R. K. Daouk, D. Galasko, P. S. Dulai, T. I. Kalashnikova, C. Wittenberg, R. Terkeltaub, M. M. Doty, J. H.

Kim, K. E. Rhee, J. Beauchamp-Walters, K. P. Wright, Jr., M. G. Dominguez-Bello, M. Manary, M. F. Oliveira, B. S. Boland, N. P. Lopes, M. Guma, A. D. Swafford, R. J. Dutton, R. Knight and P. C. Dorrestein, *Nat. Biotechnol.*, 2022, DOI: 10.1038/s41587-022-01368-1.

10. J. L. Steenwyk, T. J. Buida, 3rd, Y. Li, X. X. Shen and A. Rokas, *PLoS Biol.*, 2020, **18**, e3001007.
11. D. Darriba, D. Posada, A. M. Kozlov, A. Stamatakis, B. Morel and T. Flouri, *Mol Biol Evol*, 2019, **37**, 291–294.
12. A. M. Kozlov, D. Darriba, T. Flouri, B. Morel and A. Stamatakis, *Bioinformatics*, 2019, **35**, 4453–4455.
13. C. Xiang, S. Yao, R. Wang and L. Zhang, *Beilstein J.Org. Chem.* 2024, **20**, 1476–1485.
14. F. Sievers, A. Wilm, D. Dineen, T. J. Gibson, K. Karplus, W. Li, R. Lopez, H. McWilliam, M. Remmert, J. Söding, J. D. Thompson and D. G. Higgins, *MolSyst Biol*, 2011, **7**, MSB201175.
15. S. Whelan and N. Goldman, *Mol Biol and Evol*, 2001, **18**, 691–699.
16. J. Felsenstein, *Evolution*, 1985, **39**, 783–791.
17. I. Letunic and P. Bork, *Nucleic Acids Res*, 2024, **52**, W78–W82.
18. T. Stachelhaus, H. D. Mootz and M. A. Marahiel, *Chem Biol*, 1999, **6**, 493–505.
19. Z. Zhang, Y. Zhou, S. Xie, R.-Z. Liu, Z. Huang, P. Saravana Kumar, G. Feng, F. Yuan and L. Zhang, *J. Am. Chem. Soc.* 2025, **147**, 32662–32670.
20. B. R. Terlouw, C. Huang, D. Meijer, J. D. D. Cediel-Becerra, M. L. Rothe, M. Jenner, S. Zhou, Y. Zhang, C. D. Fage, Y. Tsunematsu, G. P. van Wezel, S. L. Robinson, F. Alberti, L. M. Alkhalaf, M. G. Chevrette, G. L. Challis and M. H. Medema, *bioRxiv*, 2025, DOI: 10.1101/2025.01.08.631717, 2025.2001.2008.631717.
21. P. Pracht, S. Grimme, C. Bannwarth, F. Bohle, S. Ehlert, G. Feldmann, J. Gorges, M. Muller, T. Neudecker, C. Plett, S. Spicher, P. Steinbach, P. A. Wesolowski and F. Zeller, *J Chem Phys*, 2024, **160**.
22. N. Grimblat, M. M. Zanardi and A. M. Sarotti, *J Org Chem*, 2015, **80**, 12526–12534.
23. F. Grundmann, M. Kaiser, M. Kurz, M. Schiell, A. Batzer and H. B. Bode, *RSC Advances*, 2013, **3**.
24. J. Bus, I. Sies and M. S. F. Lie Ken Jie, *Chem. Phys. Lipids*, 1976, **17**, 501–518.
25. E. Alexandri, R. Ahmed, H. Siddiqui, M. I. Choudhary, C. G. Tsiafoulis and I. P. Gerothanassis, *Molecules*, 2017, **22**.
26. D. Sinnaeve, M. Foroozandeh, M. Nilsson and G. A. Morris, *Angew. Chem. Int. Ed. Engl.*, 2016, **55**, 1090–1093.
27. K. Ermanis, K. E. Parkes, T. Agback and J. M. Goodman, *Org Biomol Chem*, 2016, **14**, 3943–3949.
28. E. Ueyama, N. Suzuki and K. Kano, *J Pharm Sci*, 2013, **102**, 104–113.

29. S. Sharma, K. M. Erickson and J. M. Troutman, *ACS Chem. Biol.*, 2017, **12**, 92–101.
30. Q. Li, H. Feng, Q. Tian, Y. Xiang, X. Wang, Y. X. He and K. Zhu, *Cell Chem Biol*, 2024, **31**, 1874–1884 e1876.
31. R. Sarker, C. M. Tse, R. Lin, G. McNamara, V. Singh and M. Donowitz, *Cell Physiol Biochem*, 2022, **56**, 39–49.
32. M. A. Farha, C. P. Verschoor, D. Bowdish and E. D. Brown, *Chem Biol*, 2013, **20**, 1168–1178.
33. W. Kim, G. Zou, T. P. A. Hari, I. K. Wilt, W. Zhu, N. Galle, H. A. Faizi, G. L. Hendricks, K. Tori, W. Pan, X. Huang, A. D. Steele, E. E. Csatary, M. M. Dekarske, J. L. Rosen, N. Q. Ribeiro, K. Lee, J. Port, B. B. Fuchs, P. M. Vlahovska, W. M. Wuest, H. Gao, F. M. Ausubel and E. Mylonakis, *Proc Natl Acad Sci U S A*, 2019, **116**, 16529–16534.
34. M. Song, Y. Liu, T. Li, X. Liu, Z. Hao, S. Ding, P. Panichayupakaranant, K. Zhu and J. Shen, *Adv Sci (Weinh)*, 2021, **8**, e2100749.
35. B. Y. Kim, H. Y. Weon, S. H. Yoo, W. M. Chen, S. W. Kwon, S. J. Go and E. Stackebrandt, *Int J Syst Evol Microbiol*, 2006, **56**, 1761–1764.
36. P. A. W. Martin, D. Gundersen-Rindal, M. Blackburn and J. Buyer, *Int J Syst Evol Microbiol*, 2007, **57**, 993–999.
37. C. Diaz-Cardenas, B. K. C. Patel and S. Baena, *Int J Syst Evol Microbiol*, 2010, **60**, 1437–1443.
38. M. Song, Y. Liu, X. Huang, S. Ding, Y. Wang, J. Shen and K. Zhu, *Nat Microbiol*, 2020, **5**, 1040–1050.
39. Q. Li, S. Chen, K. Zhu, X. Huang, Y. Huang, Z. Shen, S. Ding, D. Gu, Q. Yang, H. Sun, F. Hu, H. Wang, J. Cai, B. Ma, R. Zhang and J. Shen, *Nat. Commun.*, 2022, **13**, 1888.
40. W. Peng, H. Li, X. Zhao, B. Shao and K. Zhu, *J Agric Food Chem*, 2022, **70**, 2722–2732.
41. J. Jumper, R. Evans, A. Pritzel, T. Green, M. Figurnov, O. Ronneberger, K. Tunyasuvunakool, R. Bates, A. Zidek, A. Potapenko, A. Bridgland, C. Meyer, S. A. A. Kohl, A. J. Ballard, A. Cowie, B. Romera-Paredes, S. Nikolov, R. Jain, J. Adler, T. Back, S. Petersen, D. Reiman, E. Clancy, M. Zielinski, M. Steinegger, M. Pacholska, T. Berghammer, S. Bodenstein, D. Silver, O. Vinyals, A. W. Senior, K. Kavukcuoglu, P. Kohli and D. Hassabis, *Nature*, 2021, **596**, 583–589.
42. M. Leesong, B. S. Henderson, J. R. Gillig, J. M. Schwab and J. L. Smith, *Structure*, 1996, **4**, 253–264.
43. M. van Kempen, S. S. Kim, C. Tumescheit, M. Mirdita, J. Lee, C. L. M. Gilchrist, J. Soding and M. Steinegger, *Nat Biotechnol*, 2024, **42**, 243–246.
