# Supplementary material for: Lipid-donor-anchored genome mining uncovers dioxanopeptins, antibacterial lipopeptides with a 1,3-dioxane functionalized polyunsaturated lipid tail
Source: Chem Sci. 2026 Feb 27;17(16):8229–41. doi: 10.1039/d6sc00003g (PMC12964715; doi:10.1039/d6sc00003g)
Supplement: SC-017-D6SC00003G-s002 [file SC-017-D6SC00003G-s002.pdf]

Supplementary Information (SI) for Chemical Science.
This journal is © The Royal Society of Chemistry 2026

Supplementary Information

Lipid-donor-anchored genome mining uncovers dioxanopeptins, antibacterial lipopeptides with a 1,3-dioxane functionalized polyunsaturated lipid tail

Ying Chen[1,2#], Yunsheng Chen[2#], Hao Xiang[2,6#], Changqi Luo,[2] Jiaqi Duan[2], Kun Hu[3,6], Xiaohong Zheng[4], Jing Liu[5], Yongbo Xue[1*], Yi-Ming Shi[2,6*]

[1]School of Pharmaceutical Sciences (Shenzhen), Sun Yat-sen University, Shenzhen, China

[2]State Key Laboratory of Quantitative Synthetic Biology, Center for Synthetic Biochemistry, Shenzhen Institute of Synthetic Biology, Shenzhen Institutes of Advanced Technology, Chinese Academy of Sciences, Shenzhen, China

[3]State Key Laboratory of Phytochemistry and Natural Medicines, Kunming Institute of Botany, Chinese Academy of Sciences, Kunming, China

[4]Equipment Public Service Center, South China Sea Institute of Oceanology, Guangzhou, China

[5]Department of Natural Products in Organismic Interactions, Max Planck Institute for Terrestrial Microbiology, Marburg, Germany

[6]University of Chinese Academy of Sciences, Beijing, China

#These authors contributed equally to the work.

*Corresponding author

Supplementary Table 9 | Experimental and calculated ^{13}C NMR chemical shifts of **1T-a** and **1T-b.**

**No.**	$\delta_{exptl.}$	**1T-a-**$\delta_{calcd.}$	**1T-b-**$\delta_{calcd.}$	**No.**	$\delta_{exptl.}$	**1T-a-**$\delta_{calcd.}$	**1T-b-**$\delta_{calcd.}$
21	129.7	126.0	128.5	37	173.6	174.1	173.3
22	129.6	131.7	128.7	38	171.2	169.8	168.5
23	29.2	34.5	32.8	39	49.4	53.7	50.1
24	33.2	34.2	33.9	40	17	18.5	17.1
25	45.9	51.5	45.6	41	69.5	74.7	73.7
26	37.1	43.4	47.1	42	39.8	42.3	40.7
27	173	171.2	169.1	43	172	172.4	175.9
28	52.7	55.6	55.8	44	60.8	58.2	58.4
29	39.4	40.4	37.3	45	28.7	31.5	30.2
30	24.3	28.2	27.7	46	19.3	20.5	21.0
31	20.4	22.2	23.2	47	18.5	17.4	19.4
32	23.4	21.6	20.6	48	170.6	169.1	172.3
33	172.1	170.5	169.8				
34	54.7	58.7	61.1	R^2	-	0.9983	0.9976
35	27.5	27.2	27.3	**MAE**	-	2.5	2.4
36	31.8	34.4	34.3	**CMAE**	-	2.0	2.2

Supplementary Table 10 | Experimental and calculated ^{1}H NMR chemical shifts of **1T-a** and **1T-b.**

No.	$\delta_{exptl.}$	1T-a-$\delta_{calcd.}$	1T-b-$\delta_{calcd.}$	No.	$\delta_{exptl.}$	1T-a-$\delta_{calcd.}$	1T-b-$\delta_{calcd.}$
21	5.37	5.91	5.72	35a	1.83	2.08	1.91
22	5.37	5.90	5.67	35b	1.83	1.97	1.83
23a	1.91	2.18	1.99	36a	1.98	2.33	2.25
23b	1.91	1.94	2.15	36b	1.98	2.28	2.49
24a	1.29	1.30	1.26	39	3.42	3.85	3.68
24b	1.33	1.59	1.42	40	1.02	1.23	1.14
25	3.86	3.66	4.23	41	3.86	3.22	2.82
26a	2.45	2.33	2.39	42a	2.10	2.68	2.42
26b	2.30	2.24	1.98	42b	2.07	2.05	1.62
28	3.98	3.86	3.20	44	3.76	4.55	4.02
29a	1.56	1.79	2.06	45	1.96	2.39	2.08
29b	1.49	1.48	1.28	46	0.90	0.74	0.96
30	1.72	1.59	1.65	47	0.88	0.83	0.81
31	0.79	0.81	0.83	**R^2**	-	0.9550	0.9284
32	0.89	0.87	0.84	**MAE**	-	0.24	0.26
34	4.06	3.94	3.62	**CMAE**	-	0.23	0.27

Supplementary Table 11 | DP4+ probability analysis of **1a** and **1b**.

DP4+ probability	1a	1b
**DP4+ (H data)**	99.98%	0.02%
**DP4 (C data)**	100.00%	0.00%
**DP4+ (all data)**	100.00%	0.00%

Supplementary Table 12 | Conformational analysis of the B3LYP-D3BJ/6-31G(d) optimized conformers of **1T-a** in the gas phase (T=298.15 K)

Conformer	E (Hartree)[a]	C (Hartree)[b]	G (kcal/mol)[c]	ΔG (kcal/mol)[d]	Population[e]
**1T-a-1**	-1990.240548	0.707879	-1248431.749823	0.0	22.33%
**1T-a-2**	-1990.248762	0.716164	-1248431.705333	0.044490	20.71%
**1T-a-3**	-1990.240069	0.707815	-1248431.489649	0.260174	14.39%
**1T-a-4**	-1990.239143	0.706918	-1248431.471413	0.278409	13.95%
**1T-a-5**	-1990.247261	0.715216	-1248431.357949	0.391874	11.52%
**1T-a-6**	-1990.245716	0.714112	-1248431.081667	0.668156	7.22%
**1T-a-7**	-1990.243968	0.712393	-1248431.063513	0.686309	7.01%
**1T-a-8**	-1990.243773	0.713040	-1248430.535083	1.214740	2.87%

[a]Electronic energy obtained at M06-2X-D3/6-311+G (2d, p) level of theory; [b]Thermal correction to Gibbs free energy obtained at B3LYP-D3BJ/6-31G(d) level of theory; [c]Gibbs free energy (E + C); [d]The relative Gibbs free energy; [e]The Boltzmann distribution of each conformer.

Supplementary Table 13 | Atomic coordinates (Å) of **1T-a-1** obtained at the B3LYP-D3BJ/6-31G(d) level of theory in the gas phase.

C	-3.483753	-3.348557	-1.666303	H	-2.108583	-1.217344	-2.734736
C	-3.954119	-1.987580	-2.097354	H	-4.294209	2.125806	-1.247800
C	-3.181149	-1.037209	-2.632086	H	-2.932009	4.137559	-1.807069
C	-3.684785	0.261515	-3.204632	H	-2.916462	3.713317	-0.078780
C	-2.909707	1.528481	-2.798390	H	-1.907068	0.540034	-0.592732
C	-3.213184	2.018825	-1.379666	H	-2.024500	0.297255	2.307274
C	-2.583062	3.404772	-1.075328	H	-4.344292	0.072010	3.121825
C	-1.069590	3.342120	-1.155172	H	-5.698701	-1.935279	2.607421
C	0.898514	2.376939	-0.094596	H	-5.392437	-1.001333	1.135298
C	1.467897	2.114968	1.311877	H	-4.486686	-2.501264	1.458681
C	1.443060	3.332102	2.250541	H	-2.437595	-1.044553	4.314487
C	1.993186	2.925419	3.623084	H	-2.663100	-2.495624	3.327080
C	2.222188	4.518706	1.672432	H	-3.921853	-2.004654	4.471077
C	0.981198	1.089619	-0.938739	H	-2.613078	-1.735494	0.258168
C	2.578340	-0.442274	-2.026071	H	-1.103121	-3.343071	0.044031
C	4.025147	-0.360138	-2.563050	H	0.006288	-2.225016	-0.724124
C	5.127375	-0.740278	-1.563095	H	0.570958	-3.936932	1.756350
C	5.209646	0.133310	-0.315970	H	2.985026	-3.518419	0.644156
C	2.426089	-1.737643	-1.221329	H	-1.057684	2.414448	0.659294
C	2.240231	-2.791064	0.983827	H	2.147044	-0.721226	0.518900
C	2.561097	-2.404870	2.426484	H	1.496318	-4.276893	-0.866319
C	0.872870	-3.517406	0.789987	H	2.577358	-3.301415	3.054210
C	-0.279219	-2.652959	0.242978	H	1.811934	-1.714635	2.817780
C	-0.766624	-1.503510	1.104671	H	3.549070	-1.932601	2.478455
C	-2.795324	-0.202112	1.708800	H	1.887409	-0.531120	-2.869025
C	-3.851000	-0.792971	2.666563	H	4.194787	0.650325	-2.947550
C	-3.178931	-1.629621	3.758543	H	4.101199	-1.051044	-3.406345

C	-4.916860	-1.601476	1.916899	H	3.028291	1.331825	-0.975560
C	-3.413353	0.861817	0.781131	H	6.098394	-0.660446	-2.070216
N	-0.458012	2.864744	-0.019353	H	5.019657	-1.791277	-1.272949
N	2.244457	0.757881	-1.281731	H	5.956015	0.113532	1.585948
N	5.846783	-0.430111	0.741145	H	6.173169	-1.384282	0.730871
N	2.387309	-1.619737	0.121189	H	1.489238	3.138927	-0.609322
N	-2.091120	-1.230887	0.962196	H	0.909456	1.284829	1.763022
N	-2.780994	1.026628	-0.404389	H	2.507974	1.780022	1.205107
O	0.005086	0.390851	-1.235173	H	0.397629	3.643089	2.380084
O	-4.391433	1.522824	1.126812	H	1.936879	3.757808	4.333551
O	4.775004	1.285781	-0.262616	H	1.433779	2.081249	4.042811
O	2.436034	-2.824275	-1.812881	H	3.046057	2.624097	3.545560
O	1.038498	-4.631890	-0.076903	H	2.254172	5.348455	2.387556
O	-0.431561	3.644326	-2.156927	H	1.764995	4.895465	0.751725
O	-0.035757	-0.850454	1.856716	H	3.257120	4.230927	1.445359
H	-1.831884	1.363010	-2.908499	H	-5.022998	-1.794041	-1.991750
H	-3.165753	2.338318	-3.491033	H	-3.996358	-4.137126	-2.233024
H	-3.623722	0.183092	-4.300735	H	-2.408060	-3.473048	-1.826542
H	-4.750049	0.382204	-2.966491	H	-3.703025	-3.549955	-0.607332

Supplementary Table 14 | Atomic coordinates (Å) of **1T-a-2** obtained at the B3LYP-D3BJ/6-31G(d) level of theory in the gas phase.

C	5.112017	-3.324785	-0.860369	H	5.022792	-1.800459	-3.131231
C	5.097904	-1.835102	-1.039254	H	2.446348	-0.740429	-1.945165
C	5.053301	-1.203901	-2.216321	H	1.158087	1.804109	-3.002854
C	4.982567	0.287060	-2.397683	H	0.937109	0.135596	-3.555254
C	3.622250	0.752493	-2.953027	H	3.054341	0.203750	-0.024039
C	2.446748	0.348423	-2.050384	H	3.521827	1.998429	1.562449
C	1.100780	0.764914	-2.675088	H	2.706376	4.320349	1.241231
C	-0.108140	0.568331	-1.765849	H	0.359346	3.995280	0.624791
C	-1.811214	1.860947	-0.457262	H	-0.017418	3.282463	2.210575
C	-2.984825	1.932544	-1.449926	H	0.457579	4.979224	2.090690
C	-4.316174	2.443939	-0.863900	H	3.627718	3.528576	3.427931
C	-4.234923	3.926150	-0.475789	H	2.009686	3.019711	3.936299
C	-5.448233	2.196485	-1.868515	H	2.359282	4.739656	3.690380
C	-1.934719	0.835054	0.687167	H	0.669451	1.238647	1.891848
C	-3.172729	-1.100382	1.589490	H	1.506278	-1.776404	3.249982
C	-4.444009	-1.892555	1.219861	H	0.197322	-0.797391	2.537503
C	-4.283984	-2.883750	0.049520	H	1.269405	-1.438418	0.235236
C	-3.854075	-2.188684	-1.237106	H	-0.037266	-3.622429	1.889462
C	-2.015025	-2.082682	1.838980	H	0.054422	2.514791	-1.259304
C	0.084131	-3.036974	0.975170	H	-1.139675	-1.572828	0.049948
C	0.264168	-3.958549	-0.231951	H	3.171709	-2.230510	1.435314
C	1.281492	-2.090205	1.126370	H	0.317893	-3.367654	-1.155247
C	1.202479	-1.186164	2.380285	H	-0.575695	-4.659757	-0.304717
C	2.194087	-0.077297	2.122441	H	1.189125	-4.531546	-0.140523
C	2.479760	2.217701	1.306298	H	-3.331368	-0.601999	2.550307
C	2.122326	3.613854	1.843221	H	-5.244719	-1.183392	0.985933

C	2.557133	3.732046	3.309229	H	-4.742460	-2.455599	2.107654
C	0.639854	3.980070	1.680150	H	-3.470683	-0.113110	-0.265917
C	2.357466	2.091785	-0.234257	H	-5.256500	-3.352985	-0.139742
N	-0.542946	1.699025	-1.167357	H	-3.585848	-3.681234	0.320229
N	-2.904342	-0.088456	0.575180	H	-2.349369	-2.141542	-2.581761
N	-2.809139	-2.738077	-1.903314	H	-2.202939	-3.382232	-1.414035
N	-1.126663	-2.236390	0.823386	H	-1.720786	2.818114	0.062661
N	1.687759	1.159789	1.936161	H	-2.677299	2.611959	-2.255116
N	2.621785	0.843267	-0.683832	H	-3.126659	0.953198	-1.920507
O	-1.181460	0.894238	1.667624	H	-4.546625	1.871328	0.044272
O	1.999222	3.023695	-0.963201	H	-5.190442	4.272495	-0.066802
O	-4.427056	-1.168362	-1.634266	H	-3.467348	4.120395	0.282253
O	-1.996350	-2.726003	2.882977	H	-4.004290	4.544414	-1.352866
O	2.473597	-2.857427	1.155617	H	-5.543638	1.129235	-2.090342
O	-0.649171	-0.537912	-1.614008	H	-5.254630	2.727049	-2.809889
O	3.399519	-0.344300	1.927294	H	-6.406042	2.556409	-1.475666
H	3.621038	1.842791	-3.065439	H	5.121612	-1.239276	-0.124225
H	3.458625	0.320345	-3.949542	H	4.223596	-3.649276	-0.305560
H	5.169740	0.786732	-1.439919	H	5.128370	-3.842383	-1.825784
H	5.768691	0.623514	-3.087137	H	5.989864	-3.647840	-0.285230

Supplementary Table 15 | Atomic coordinates (Å) of **1T-a-3** obtained at the B3LYP-D3BJ/6-31G(d) level of theory in the gas phase.

C	1.415694	1.498541	-3.541197	H	3.652475	0.305556	-2.449549
C	1.837461	0.060027	-3.462626	H	4.284000	-2.543404	-0.323924
C	2.958685	-0.401981	-2.901647	H	2.892515	-4.486592	0.370150
C	3.354952	-1.862579	-2.875829	H	3.068399	-3.260134	1.647413
C	2.690394	-2.721819	-1.779900	H	1.968486	-0.733226	-0.183586
C	3.194544	-2.454334	-0.356833	H	3.100806	1.209667	1.972114
C	2.621772	-3.471143	0.669648	H	5.379441	1.753080	1.325261
C	1.107913	-3.386459	0.726389	H	5.894930	2.858759	-0.856804
C	-0.735597	-1.912614	1.311527	H	5.370942	1.183990	-1.076284
C	-1.070108	-0.780872	2.296445	H	4.255310	2.529962	-1.420821
C	-0.917818	-1.123489	3.786305	H	3.838764	3.534728	2.234238
C	-1.169658	0.141563	4.616506	H	3.394081	3.981586	0.583422
C	-1.839560	-2.271284	4.209698	H	5.057722	4.218929	1.136968
C	-0.880221	-1.361120	-0.117032	H	2.142111	1.308115	-0.810878
C	-2.410575	-0.808872	-1.938509	H	0.188714	3.266387	-0.811241
C	-3.754992	-1.314222	-2.507512	H	-0.173173	1.537775	-0.844707
C	-5.018738	-0.573618	-2.050074	H	-1.444417	1.925357	1.380154
C	-5.315724	-0.625621	-0.555775	H	-2.411233	3.206591	-1.200667
C	-2.388878	0.731449	-1.870676	H	1.287757	-1.687163	1.828810
C	-2.574846	2.644893	-0.277217	H	-2.875182	0.585071	0.085858
C	-3.815542	3.163192	0.446063	H	-0.468294	3.875977	1.840247
C	-1.319367	2.702010	0.609681	H	-3.666769	4.200239	0.752516
C	-0.046468	2.397148	-0.184467	H	-4.012830	2.571123	1.348996
C	1.115777	2.121493	0.751848	H	-4.691944	3.095591	-0.206485
C	3.391150	1.164630	0.916573	H	-1.609105	-1.071004	-2.632686
C	4.565411	2.147566	0.708142	H	-3.841678	-2.383358	-2.288216
C	4.192367	3.549692	1.198261	H	-3.705004	-1.212900	-3.595318

C	5.045719	2.175726	-0.746627	H	-2.898046	-1.823190	-0.140311
C	3.826152	-0.284402	0.630471	H	-5.883894	-1.005984	-2.570357
N	0.616373	-2.385029	1.532595	H	-4.969372	0.476280	-2.361714
N	-2.111494	-1.438121	-0.658985	H	-6.558040	0.209259	0.834696
N	-6.325840	0.184110	-0.148330	H	-6.770794	0.844705	-0.767232
N	-2.762305	1.249001	-0.670052	H	-1.419414	-2.755791	1.437753
N	2.193841	1.533285	0.174947	H	-0.428154	0.077942	2.064435
N	2.895401	-1.085077	0.049381	H	-2.106082	-0.463398	2.113071
O	0.059247	-0.800579	-0.696505	H	0.119662	-1.440729	3.957421
O	4.952227	-0.667283	0.938882	H	-1.010044	-0.048908	5.683597
O	-4.723316	-1.355698	0.241699	H	-0.502642	0.954957	4.308572
O	-2.114998	1.401922	-2.860367	H	-2.203583	0.489453	4.491643
O	-1.227923	3.969911	1.235088	H	-1.747431	-2.467647	5.283632
O	0.363805	-4.101327	0.065187	H	-1.599804	-3.200471	3.682096
O	1.068126	2.400172	1.957176	H	-2.889450	-2.024796	4.003672
H	1.605648	-2.582546	-1.820054	H	1.152828	-0.655643	-3.922087
H	2.875487	-3.780148	-2.001366	H	0.393481	1.634991	-3.170044
H	3.091854	-2.312545	-3.841413	H	2.093226	2.154069	-2.979336
H	4.445806	-1.944667	-2.785041	H	1.413373	1.848972	-4.582614

Supplementary Table 16 | Atomic coordinates (Å) of **1T-a-4** obtained at the B3LYP-D3BJ/6-31G(d) level of theory in the gas phase.

C	4.108773	1.983269	-2.543044	H	1.811917	0.555419	-3.060628
C	3.904237	0.537115	-2.897858	H	2.893772	-3.324122	-1.677957
C	2.724704	-0.037760	-3.153004	H	0.801743	-4.700950	-1.737110
C	2.545401	-1.442447	-3.665213	H	1.377798	-4.300807	-0.101868
C	1.480659	-2.299773	-2.956317	H	1.410166	-0.980544	-0.694765
C	1.919131	-2.834520	-1.589479	H	2.275112	-0.666315	2.021474
C	0.934829	-3.890796	-1.015799	H	4.643075	-1.284037	2.364884
C	-0.422920	-3.274519	-0.734941	H	5.557884	-0.727793	0.116381
C	-1.565871	-1.677654	0.710590	H	5.323558	1.008078	0.444925
C	-1.580477	-1.231151	2.183134	H	6.474028	0.074162	1.401326
C	-1.726142	-2.376948	3.197808	H	4.047064	1.724045	2.603596
C	-1.712080	-1.803097	4.619939	H	5.266951	0.847205	3.540750
C	-2.989282	-3.208898	2.948457	H	3.543338	0.487327	3.763935
C	-1.415166	-0.455831	-0.214957	H	3.068149	0.901305	-0.339146
C	-2.617990	1.472199	-1.136420	H	2.175212	2.882136	-0.562952
C	-4.091010	1.790334	-1.434703	H	0.565006	2.241334	-0.822869
C	-4.855791	0.636807	-2.102994	H	1.406928	4.269627	1.314760
C	-5.489991	-0.353826	-1.127094	H	-1.210791	4.742408	0.948860
C	-1.910258	2.732077	-0.618532	H	0.360783	-2.465254	0.960267
C	-0.708522	3.810034	1.224765	H	-1.476948	1.833237	1.147387
C	-0.679259	3.705438	2.748603	H	-0.100986	4.695904	-1.006620
C	0.699286	3.906781	0.560357	H	-0.167129	4.577861	3.166843
C	1.251855	2.602565	-0.049856	H	-0.153450	2.803274	3.066471
C	1.510664	1.427974	0.875777	H	-1.699181	3.687693	3.148077
C	3.028251	-0.508300	1.240459	H	-2.104402	1.226752	-2.073015
C	4.399510	-0.313861	1.919258	H	-4.094027	2.662688	-2.092625
C	4.307504	0.744630	3.021819	H	-4.596369	2.081022	-0.506715

C	5.498394	0.027566	0.905733	H	-3.386439	-0.002221	0.165339
C	3.018593	-1.756922	0.334344	H	-4.185340	0.069100	-2.763241
N	-0.511828	-2.636924	0.478747	H	-5.647927	1.039524	-2.745405
N	-2.511424	0.334999	-0.242683	H	-6.861544	-1.862343	-1.079005
N	-6.477074	-1.122822	-1.650673	H	-6.718077	-1.096475	-2.629885
N	-1.515753	2.724089	0.668525	H	-2.514073	-2.157555	0.452638
N	2.590829	0.678492	0.524128	H	-0.664750	-0.660943	2.384609
N	2.100179	-1.728812	-0.659891	H	-2.415794	-0.532346	2.319326
O	-0.387381	-0.186610	-0.845768	H	-0.857995	-3.041629	3.090577
O	3.774645	-2.706011	0.535632	H	-2.576886	-1.147395	4.783979
O	-5.154266	-0.464309	0.054206	H	-1.753539	-2.602684	5.368089
O	-1.746605	3.688261	-1.387146	H	-0.806725	-1.212863	4.802981
O	0.687918	4.895450	-0.461721	H	-3.115301	-3.967112	3.729543
O	-1.357445	-3.294520	-1.528864	H	-2.948274	-3.729968	1.986404
O	0.798516	1.142923	1.844308	H	-3.883568	-2.572008	2.951261
H	0.547862	-1.734292	-2.846824	H	4.808912	-0.066956	-2.986047
H	1.242200	-3.161220	-3.590694	H	4.756207	2.477486	-3.279400
H	2.250742	-1.370754	-4.723300	H	3.161328	2.530932	-2.517324
H	3.511218	-1.965027	-3.653008	H	4.608794	2.107553	-1.571524

Supplementary Table 17 | Atomic coordinates (Å) of **1T-a-5** obtained at the B3LYP-D3BJ/6-31G(d) level of theory in the gas phase.

C	-6.349242	-1.196516	-0.477492	H	-5.779549	0.452746	1.633191
C	-5.529400	-1.432671	0.756444	H	-2.031059	-1.092668	2.077584
C	-5.330428	-0.538302	1.729092	H	-1.113440	1.651025	3.031728
C	-4.549178	-0.784473	2.989962	H	-0.633854	0.057212	3.643698
C	-3.359402	0.165700	3.218171	H	-3.057996	-0.221102	0.283529
C	-2.214228	-0.019221	2.207535	H	-3.736008	1.438581	-1.330967
C	-0.916457	0.616584	2.747028	H	-3.144880	3.849007	-1.111497
C	0.281422	0.542431	1.806451	H	-1.050774	4.700385	-2.170521
C	1.732973	1.930844	0.317288	H	-0.732303	3.780324	-0.694681
C	2.959617	2.164682	1.215523	H	-0.421667	3.054085	-2.288790
C	4.184196	2.784793	0.513090	H	-2.548453	2.541272	-3.826592
C	3.915286	4.227600	0.065657	H	-3.043358	4.227316	-3.592641
C	5.400067	2.713198	1.445108	H	-4.160778	2.909230	-3.193854
C	1.874717	0.865239	-0.789158	H	-0.841918	0.954214	-1.797131
C	3.204452	-0.998022	-1.706701	H	-1.492232	-2.288231	-2.852648
C	4.573050	-1.652927	-1.429276	H	-0.227707	-1.096715	-2.438304
C	4.623885	-2.575030	-0.194870	H	-0.911792	-1.387775	0.004305
C	4.270344	-1.836765	1.090899	H	0.247517	-3.776981	-1.454243
C	2.114577	-2.085070	-1.770327	H	-0.164529	2.408898	1.183022
C	0.188181	-3.098202	-0.600035	H	1.429005	-1.535461	0.082720
C	0.161466	-3.884310	0.711403	H	-2.960227	-2.477392	-0.847584
C	-1.049020	-2.195084	-0.731584	H	-0.749506	-4.482025	0.778508
C	-1.175382	-1.533945	-2.126978	H	0.185621	-3.195876	1.565096
C	-2.253313	-0.491917	-1.953769	H	1.027127	-4.555073	0.769377
C	-2.709373	1.776877	-1.156217	H	3.218354	-0.555000	-2.706664
C	-2.544171	3.186973	-1.746863	H	5.325282	-0.864539	-1.322707
C	-3.109170	3.217941	-3.172346	H	4.831057	-2.245759	-2.310517

C	-1.097789	3.702518	-1.720886	H	3.587913	0.133750	0.050775
C	-2.458111	1.706010	0.373092	H	5.648745	-2.949108	-0.086433
N	0.537044	1.676354	1.121141	H	3.975415	-3.443545	-0.342123
N	2.945586	0.054848	-0.730456	H	2.932265	-1.842867	2.601889
N	3.367431	-2.436711	1.905231	H	2.773132	-3.160206	1.524060
N	1.373430	-2.246866	-0.644594	H	1.509227	2.848717	-0.232447
N	-1.851010	0.790610	-1.816875	H	2.640212	2.842684	2.017200
N	-2.571420	0.451715	0.866907	H	3.238511	1.226202	1.706782
O	1.033426	0.787763	-1.693654	H	4.416540	2.193412	-0.382513
O	-2.121219	2.687968	1.046155	H	4.799621	4.653128	-0.421307
O	4.778579	-0.743306	1.360707	H	3.085849	4.299067	-0.647358
O	2.022487	-2.783953	-2.773733	H	3.672145	4.861871	0.927806
O	-2.215319	-2.939910	-0.411614	H	5.625720	1.675684	1.709296
O	0.973418	-0.483752	1.709055	H	5.211845	3.271160	2.371646
O	-3.432641	-0.847257	-1.757230	H	6.285038	3.150165	0.968518
H	-3.685710	1.212403	3.183021	H	-5.066217	-2.417139	0.850794
H	-2.963188	-0.011175	4.226421	H	-7.163907	-1.928925	-0.554543
H	-5.224914	-0.680535	3.851732	H	-6.794344	-0.195281	-0.471616
H	-4.189087	-1.822016	3.002943	H	-5.731432	-1.290832	-1.376383

Supplementary Table 18 | Atomic coordinates (Å) of **1T-a-6** obtained at the B3LYP-D3BJ/6-31G(d) level of theory in the gas phase.

Arial	-5.539312	2.281752	-1.507993	H	-5.126415	0.323548	-3.411857
C	-5.447870	0.790571	-1.390695	H	-2.586460	-0.280977	-2.421893
C	-5.240581	-0.064260	-2.396678	H	-1.219351	-2.986366	-2.215289
C	-5.102271	-1.552859	-2.224023	H	-1.027529	-1.736718	-3.457622
C	-3.717887	-2.090205	-2.638892	H	-3.146124	-0.244960	-0.260201
C	-2.558525	-1.295696	-2.017648	H	-3.567759	-1.122918	1.932329
C	-1.195028	-1.909598	-2.390016	H	-2.750895	-3.306829	2.719024
C	-0.017847	-1.279514	-1.651081	H	-0.418388	-3.341015	2.009635
C	1.701333	-1.796520	0.108477	H	-0.006545	-1.972634	3.067237
C	2.919371	-2.253344	-0.711341	H	-0.497229	-3.518726	3.768150
C	4.260048	-2.280902	0.043772	H	-3.641839	-1.581793	4.300894
C	4.206550	-3.157683	1.302004	H	-2.010675	-0.929848	4.519361
C	5.365299	-2.754828	-0.907482	H	-2.390668	-2.566499	5.082176
C	1.769207	-0.370397	0.688697	H	-0.752265	-0.214033	2.044302
C	2.709674	1.878158	0.548928	H	-1.897143	2.692383	2.821605
C	3.837817	2.613784	-0.189794	H	-0.446792	1.985271	2.114736
C	5.217594	1.956118	-0.070831	H	-2.627728	3.495188	0.583127
C	5.498669	0.908034	-1.144952	H	-0.530771	4.066811	-0.874578
C	1.441854	2.741668	0.477460	H	-0.108158	-2.821796	-0.349336
C	-0.804117	3.078859	-0.484170	H	0.539363	1.436601	-0.810177
C	-1.661856	2.354265	-1.519353	H	-0.190084	4.566627	1.447538
C	-1.571456	3.374793	0.842556	H	-2.533035	2.962707	-1.774182
C	-1.482732	2.258934	1.903511	H	-2.021560	1.402596	-1.128643
C	-2.316109	1.031620	1.597348	H	-1.090453	2.151858	-2.430164
C	-2.518023	-1.413720	1.806526	H	2.947722	1.828142	1.617032
C	-2.156094	-2.408417	2.924314	H	3.873116	3.618788	0.236840

C	-2.576104	-1.838448	4.284937	H	3.572260	2.716982	-1.247691
C	-0.678687	-2.827370	2.937508	H	2.980253	0.302756	-0.840916
C	-2.405877	-2.010066	0.384655	H	5.332093	1.475785	0.910787
N	0.463980	-2.032417	-0.632570	H	6.001570	2.721543	-0.127378
N	2.549397	0.524727	0.053680	H	7.038201	-0.210534	-1.896101
N	6.793589	0.507705	-1.229353	H	7.518342	0.892050	-0.642735
N	0.435312	2.311890	-0.296384	H	1.616023	-2.424828	0.996965
N	-1.761828	-0.166440	1.911043	H	2.694019	-3.269569	-1.061118
N	-2.715548	-1.114134	-0.575709	H	3.012128	-1.636081	-1.611689
O	1.154890	-0.088240	1.726040	H	4.500950	-1.257874	0.357204
O	-2.037118	-3.170150	0.159061	H	3.887369	-4.178570	1.055148
O	4.630661	0.442017	-1.883106	H	5.195580	-3.224096	1.769442
O	1.434197	3.798488	1.124607	H	3.516869	-2.761834	2.055362
O	-1.168460	4.612824	1.396944	H	5.388687	-2.133094	-1.808379
O	0.456618	-0.184752	-1.975147	H	5.194492	-3.793805	-1.216647
O	-3.460439	1.119904	1.122646	H	6.348340	-2.708520	-0.424203
H	-3.634127	-3.142668	-2.344421	H	-5.543078	0.395242	-0.379672
H	-3.608216	-2.048435	-3.731136	H	-6.531692	2.644970	-1.208319
H	-5.284763	-1.809660	-1.174316	H	-4.815303	2.753482	-0.833043
H	-5.864794	-2.080731	-2.813948	H	-5.346882	2.623664	-2.531009

Supplementary Table 19 | Atomic coordinates (Å) of **1T-a-7** obtained at the B3LYP-D3BJ/6-31G(d) level of theory in the gas phase.

C	1.737593	-2.975231	3.883714	H	0.607623	-4.637197	1.988840
C	0.595304	-2.761387	2.935135	H	-2.478258	-3.539163	-1.340271
C	0.130271	-3.656167	2.056357	H	-0.825365	-3.765346	-3.136825
C	-1.018194	-3.399102	1.118426	H	-1.874369	-2.357700	-3.427376
C	-0.554403	-3.317585	-0.343971	H	-1.528010	-0.890713	-0.483633
C	-1.634344	-2.843594	-1.329867	H	-3.950815	0.792120	-1.095848
C	-1.086452	-2.764342	-2.785138	H	-5.965220	-0.448994	-0.420816
C	0.171402	-1.917412	-2.823804	H	-6.357773	-1.087156	1.947971
C	1.005994	0.323027	-2.276419	H	-5.002767	-1.994859	1.263969
C	0.627403	1.778627	-2.606248	H	-4.710485	-0.631501	2.374892
C	1.698795	2.831431	-2.276413	H	-5.191804	1.803449	1.526378
C	2.939084	2.686549	-3.166787	H	-6.844475	1.302926	1.138798
C	1.099016	4.239765	-2.374082	H	-5.821751	2.041453	-0.109549
C	1.106084	0.132356	-0.754071	H	-2.682607	0.005511	1.451048
C	2.451384	-0.158123	1.240354	H	-1.842278	2.572381	2.426205
C	3.913095	-0.462449	1.602794	H	-0.798570	1.217650	1.974010
C	4.355244	-1.870241	1.150449	H	0.096170	2.788956	0.106669
C	4.051922	-2.051612	-0.332594	H	0.674216	3.111393	3.043760
C	1.813749	0.920796	2.138048	H	-0.984078	-0.265488	-2.606295
C	0.957459	3.269764	1.999721	H	2.254124	2.247314	0.705489
C	1.667280	4.611787	1.844223	H	-1.625314	4.213303	0.266411
C	-0.277698	3.142410	1.075938	H	1.991060	4.764929	0.807356
C	-1.292276	2.113896	1.593548	H	2.547303	4.648697	2.493347
C	-2.276150	1.754218	0.492783	H	0.989781	5.428564	2.098376
C	-3.917070	0.057348	-0.284076	H	1.839330	-1.045174	1.431198
C	-5.317836	-0.032749	0.357785	H	4.019460	-0.385119	2.688894
C	-5.822808	1.359150	0.747574	H	4.569037	0.291702	1.154236

C	-5.340878	-0.994535	1.552311	H	3.150203	0.060027	-0.774275
C	-3.508503	-1.292898	-0.909911	H	3.851199	-2.629242	1.758099
N	-0.032396	-0.565654	-2.782103	H	5.434111	-1.978160	1.301213
N	2.328761	0.184476	-0.183929	H	2.709760	-2.970836	-1.567778
N	3.144657	-3.004009	-0.646280	H	2.633563	-3.478441	0.085051
N	1.876144	2.182703	1.642950	H	1.946793	0.033492	-2.748856
N	-2.910465	0.562013	0.638773	H	0.401064	1.823092	-3.679063
N	-2.175630	-1.561236	-0.895139	H	-0.303027	2.017293	-2.073705
O	0.089016	-0.020139	-0.064769	H	2.018899	2.684662	-1.236310
O	-4.345024	-2.046016	-1.399458	H	3.687393	3.446456	-2.916736
O	4.550580	-1.296660	-1.173810	H	3.417735	1.706573	-3.060695
O	1.304654	0.609880	3.211305	H	2.672817	2.814874	-4.223282
O	-0.903497	4.403307	0.897128	H	0.286711	4.380607	-1.652928
O	1.300592	-2.408226	-2.807796	H	0.699830	4.423792	-3.379243
O	-2.482386	2.501267	-0.472107	H	1.860520	5.002006	-2.174019
H	0.294651	-2.630478	-0.387454	H	0.120287	-1.782004	2.997097
H	-0.180594	-4.292793	-0.681198	H	1.384056	-2.989290	4.923340
H	-1.783570	-4.181797	1.219068	H	2.260995	-3.918904	3.692597
H	-1.496961	-2.453125	1.393986	H	2.453612	-2.147295	3.819330

Supplementary Table 20 | Atomic coordinates (Å) of **1T-a-8** obtained at the B3LYP-D3BJ/6-31G(d) level of theory in the gas phase.

C	3.916729	1.050842	-3.061546	H	1.775304	-0.672596	-2.917317
C	3.854514	-0.424508	-2.782236	H	3.155003	-3.513645	-0.060502
C	2.739996	-1.162122	-2.764217	H	1.201969	-4.993619	0.458489
C	2.704070	-2.662985	-2.647395	H	1.685792	-3.912018	1.784580
C	1.698684	-3.247954	-1.638487	H	1.431544	-1.128660	-0.123324
C	2.138043	-3.126559	-0.175635	H	2.176149	0.371110	2.196984
C	1.236083	-3.952098	0.786802	H	4.578481	0.197715	2.739621
C	-0.179889	-3.410065	0.809571	H	6.301242	1.207287	1.281601
C	-1.467940	-1.427555	1.517830	H	5.507419	-0.139267	0.451547
C	-1.417744	-0.460648	2.722879	H	5.100982	1.545506	0.035243
C	-2.627540	0.459297	2.955847	H	3.689333	2.956650	1.710468
C	-3.925050	-0.325580	3.191976	H	4.953684	2.674369	2.916864
C	-2.322862	1.390854	4.136218	H	3.265720	2.268247	3.284265
C	-1.394182	-0.677440	0.173617	H	2.898567	0.907345	-0.606589
C	-2.721037	0.581080	-1.468596	H	1.833533	2.511201	-1.629103
C	-4.184711	0.437566	-1.914071	H	0.284115	1.697132	-1.576478
C	-4.563908	-1.006431	-2.304097	H	0.929507	4.492933	-0.502123
C	-4.203936	-1.986080	-1.188289	H	-1.718089	4.561996	-0.950845
C	-2.240949	2.038484	-1.525292	H	0.491770	-1.950652	2.035178
C	-1.140210	3.872664	-0.328268	H	-1.667965	1.969834	0.418770
C	-1.112706	4.411850	1.101140	H	-0.606744	3.797514	-2.738949
C	0.259402	3.790513	-1.011108	H	-0.682867	5.418583	1.106987
C	0.926194	2.397448	-1.031030	H	-0.512687	3.769981	1.748965
C	1.257957	1.729583	0.292366	H	-2.130017	4.474509	1.502113
C	2.934021	0.267033	1.411799	H	-2.070410	0.053956	-2.176234
C	4.257097	0.864761	1.932871	H	-4.342026	1.101374	-2.767843
C	4.026728	2.270009	2.495804	H	-4.839916	0.779765	-1.104283

C	5.351149	0.864326	0.858456	H	-3.375754	-0.289321	0.341360
C	3.075630	-1.241065	1.110675	H	-4.069524	-1.277751	-3.244111
N	-0.352460	-2.343003	1.640290	H	-5.644765	-1.057354	-2.471140
N	-2.532850	-0.026678	-0.158978	H	-2.778671	-3.409756	-0.752481
N	-3.275962	-2.918958	-1.496322	H	-2.814080	-2.897525	-2.393426
N	-1.843448	2.590680	-0.361427	H	-2.394979	-2.009096	1.525323
N	2.406049	1.000385	0.271640	H	-1.288028	-1.088593	3.614214
N	2.185253	-1.725154	0.212675	H	-0.518904	0.158569	2.618065
O	-0.386354	-0.636660	-0.539015	H	-2.764354	1.096758	2.073900
O	3.914938	-1.936696	1.679178	H	-3.816061	-0.994602	4.055217
O	-4.689325	-1.868146	-0.058085	H	-4.754452	0.357847	3.405509
O	-2.210847	2.621061	-2.615478	H	-4.220589	-0.939933	2.334220
O	0.173757	4.246619	-2.355502	H	-3.152098	2.085555	4.309383
O	-1.082475	-3.866088	0.105544	H	-1.418013	1.979609	3.948525
O	0.538870	1.798701	1.295987	H	-2.169768	0.817169	5.059080
H	0.716600	-2.776462	-1.765383	H	4.812210	-0.922339	-2.621298
H	1.557945	-4.312601	-1.857958	H	4.535461	1.254009	-3.945461
H	2.439057	-3.064047	-3.637525	H	2.923088	1.468697	-3.252546
H	3.711506	-3.039926	-2.426911	H	4.378402	1.611997	-2.236324

Supplementary Table 21 | Conformational analysis of the B3LYP-D3BJ/6-31G(d) optimized conformers of **1T-b** in the gas phase (T=298.15 K)

Conformer	E (Hartree)[a]	C (Hartree)[b]	G (kcal/mol)[c]	ΔG (kcal/mol)[d]	Population[e]
**1T-b-1**	-1990.247326	0.709229	-1248435.155811	0.0	60.06%
**1T-b-2**	-1990.245229	0.708271	-1248434.440875	0.714936	17.95%
**1T-b-3**	-1990.250337	0.713492	-1248434.369968	0.785843	15.93%
**1T-b-4**	-1990.251693	0.715761	-1248433.797562	1.358249	6.06%

[a]Electronic energy obtained at M06-2X-D3/6-311+G(2d,p) level of theory; [b]Thermal correction to Gibbs free energy obtained at B3LYP-D3BJ/6-31G(d) level of theory; [c]Gibbs free energy (E + C); [d]The relative Gibbs free energy; [e]The Boltzmann distribution of each conformer.

Supplementary Table 22 | Atomic coordinates (Å) of **1T-b-1** obtained at the B3LYP-D3BJ/6-31G(d) level of theory in the gas phase.

C	5.697721	3.250081	0.618277	H	5.478252	2.648747	-2.059494
C	5.620066	1.934028	-0.094120	H	2.870878	1.535366	-1.324869
C	5.504395	1.768195	-1.413293	H	1.363805	0.968137	-3.286426
C	5.321023	0.437241	-2.087555	H	1.317006	-0.699569	-2.761115
C	3.921119	0.285455	-2.714636	H	2.992820	-1.368951	-0.711258
C	2.777553	0.519347	-1.715154	H	3.575402	-1.803475	1.508306
C	1.411760	0.329011	-2.394775	H	2.126038	0.139761	3.358361
C	0.137654	0.611938	-1.598837	H	2.797665	-1.413953	5.208104
C	-0.917439	1.680483	0.355778	H	1.719823	-2.237082	4.064741
C	-1.821043	2.726548	-0.308833	H	3.460498	-2.552040	4.023600
C	-1.094993	4.020680	-0.712620	H	4.374512	0.388928	4.439134
C	-2.086792	4.974235	-1.389623	H	4.395909	0.916445	2.749238
C	-0.401930	4.705255	0.472373	H	5.084104	-0.660839	3.204982
C	-1.613271	0.387822	0.829280	H	0.651436	-1.339848	1.768705
C	-3.625516	-0.989891	0.898071	H	0.005675	-4.405033	0.499357
C	-5.127822	-0.857937	0.613224	H	-0.706725	-2.834004	0.919291
C	-5.827002	0.296449	1.346512	H	0.079887	-2.080992	-1.455433
C	-5.820636	1.614288	0.576468	H	-2.122926	-4.147125	-1.185165
C	-3.108708	-2.282813	0.230818	H	1.154518	1.553481	-0.071284
C	-1.710958	-3.214782	-1.579736	H	-2.164297	-1.183056	-1.218342
C	-1.909196	-3.131983	-3.092635	H	1.420682	-4.021208	-1.585247
C	-0.222275	-3.109225	-1.205716	H	-1.350760	-3.927602	-3.590269
C	0.039921	-3.328578	0.299692	H	-1.548655	-2.166410	-3.466220
C	1.424028	-2.803973	0.592892	H	-2.971513	-3.223957	-3.339935
C	2.769490	-1.101282	1.743530	H	-3.470483	-1.154367	1.969513
C	2.904519	-0.615757	3.192310	H	-5.575631	-1.806912	0.919083
C	4.270837	0.049977	3.402689	H	-5.285477	-0.758604	-0.466570

C	2.703316	-1.772668	4.177352	H	-3.412717	0.893682	-0.058956
C	2.788323	0.050427	0.720928	H	-5.349473	0.474922	2.319952
N	0.240742	1.348799	-0.473386	H	-6.867414	0.025814	1.564474
N	-2.907067	0.210851	0.502745	H	-6.790704	3.410720	0.497813
N	-6.747401	2.523023	0.978554	H	-7.449509	2.311421	1.670757
N	-2.424083	-2.121795	-0.923046	H	-0.492824	2.097811	1.273238
N	1.516330	-1.825749	1.521716	H	-2.278289	2.288866	-1.201312
N	2.928046	-0.362217	-0.559452	H	-2.638131	2.973887	0.380764
O	-0.991307	-0.437512	1.510605	H	-0.323838	3.754092	-1.447925
O	2.590102	1.229921	1.042257	H	-2.576476	4.495967	-2.245361
O	-5.031629	1.867596	-0.333829	H	-2.872174	5.282480	-0.687500
O	-3.377219	-3.364835	0.748158	H	-1.583619	5.880070	-1.746848
O	0.524935	-4.034677	-1.976993	H	-1.119918	4.917780	1.275643
O	-0.949103	0.195422	-2.022003	H	0.041895	5.658554	0.163960
O	2.412864	-3.202814	-0.057959	H	0.403107	4.093057	0.893443
H	3.801836	1.002419	-3.537186	H	5.630682	1.047441	0.540816
H	3.823679	-0.715304	-3.157847	H	5.700665	4.089853	-0.085010
H	6.067646	0.297776	-2.881338	H	4.835011	3.366389	1.287427
H	5.478773	-0.365308	-1.358370	H	6.599385	3.321142	1.240811

Supplementary Table 23 | Atomic coordinates (Å) of **1T-b-2** obtained at the B3LYP-D3BJ/6-31G(d) level of theory in the gas phase.

C	6.761100	-0.311719	0.721196	H	5.781554	-0.271529	-1.826501
C	6.070509	0.780680	-0.042436	H	2.335743	2.409388	-1.357298
C	5.637131	0.675115	-1.300418	H	0.952197	1.637054	-3.307593
C	4.971352	1.765884	-2.096273	H	1.250746	-0.034461	-2.873332
C	3.620413	1.365041	-2.724457	H	3.079383	-0.388628	-0.740079
C	2.434881	1.392138	-1.748344	H	3.806116	-0.615691	1.466290
C	1.134299	0.970854	-2.453718	H	1.933038	0.904766	3.333110
C	-0.159634	0.935446	-1.640801	H	3.909327	-1.359973	3.990604
C	-1.411418	1.697929	0.339214	H	3.009458	-0.407980	5.183095
C	-2.563965	2.481792	-0.300617	H	2.146615	-1.485893	4.068789
C	-2.203921	3.923333	-0.696569	H	4.997092	0.866176	3.176725
C	-3.414140	4.588503	-1.363161	H	4.037471	1.747038	4.372774
C	-1.709694	4.757202	0.492318	H	3.945024	2.206759	2.664578
C	-1.747354	0.268240	0.812399	H	0.868519	-0.928208	1.756537
C	-3.344628	-1.574053	0.904361	H	0.991902	-4.054787	0.432637
C	-4.835988	-1.826927	0.645288	H	-0.073937	-2.718837	0.915893
C	-5.792039	-0.893280	1.402265	H	0.497858	-1.686250	-1.406994
C	-6.132693	0.389625	0.648583	H	-1.131003	-4.238267	-1.245315
C	-2.527810	-2.690602	0.218835	H	0.624778	2.073258	-0.109580
C	-0.955849	-3.223260	-1.611179	H	-1.910255	-1.378314	-1.228923
C	-1.159923	-3.150041	-3.123862	H	2.277558	-3.249062	-1.599283
C	0.460332	-2.769538	-1.213747	H	-1.046816	-2.115713	-3.468713
C	0.769167	-2.993124	0.283505	H	-2.165949	-3.493289	-3.385259
C	1.988878	-2.164345	0.604034	H	-0.420845	-3.770633	-3.634649
C	2.847395	-0.152549	1.717585	H	-3.134140	-1.698424	1.971838
C	2.875019	0.368605	3.160169	H	-5.021924	-2.860496	0.948258
C	4.031641	1.359358	3.348221	H	-5.033787	-1.764098	-0.430632

C	2.985817	-0.790600	4.156839	H	-3.633511	0.305893	-0.039119
C	2.549935	0.950538	0.685069	H	-5.359451	-0.608183	2.371301
N	-0.222412	1.674614	-0.512946	H	-6.726582	-1.420233	1.630877
N	-2.961231	-0.228770	0.508059	H	-7.522222	1.887168	0.613151
N	-7.246957	1.035503	1.082049	H	-7.856969	0.650799	1.786880
N	-1.917904	-2.353781	-0.938544	H	-1.089571	2.205201	1.253118
N	1.829138	-1.187369	1.525034	H	-2.905689	1.948346	-1.192632
N	2.704201	0.546367	-0.593357	H	-3.406731	2.503492	0.401929
O	-0.922028	-0.376256	1.471412	H	-1.393195	3.872957	-1.435899
O	2.117547	2.069568	0.995334	H	-3.763843	4.006140	-2.223032
O	-5.454278	0.837719	-0.275596	H	-4.250403	4.672550	-0.657162
O	-2.509065	-3.809243	0.728209	H	-3.168493	5.598147	-1.711948
O	1.415002	-3.439525	-2.019503	H	-0.772762	4.372078	0.909330
O	-1.119904	0.270624	-2.052197	H	-2.456622	4.771085	1.297203
O	3.052728	-2.318444	-0.031670	H	-1.529056	5.794944	0.190049
H	3.385603	2.046210	-3.551259	H	5.917849	1.722109	0.488532
H	3.703184	0.362280	-3.167865	H	6.225810	-0.551492	1.649387
H	4.839642	2.658928	-1.471717	H	6.831735	-1.229772	0.129118
H	5.642202	2.057606	-2.917570	H	7.776609	-0.013125	1.014585

Supplementary Table 24 | Atomic coordinates (Å) of **1T-b-3** obtained at the B3LYP-D3BJ/6-31G(d) level of theory in the gas phase.

C	6.715567	-0.523140	0.245535	H	5.407507	-1.384385	-1.990690
C	6.008715	0.248612	-0.830593	H	2.256461	1.650828	-2.345062
C	5.403819	-0.296577	-1.888235	H	0.580288	0.349522	-3.682534
C	4.719321	0.458825	-2.996262	H	0.812890	-1.045030	-2.648333
C	3.272022	0.005690	-3.279697	H	2.908530	-0.730962	-0.719916
C	2.230133	0.557908	-2.295258	H	3.902972	-0.129574	1.313128
C	0.820832	0.050695	-2.653577	H	2.382845	2.202247	2.561641
C	-0.361248	0.477557	-1.782856	H	4.293877	0.193818	3.896144
C	-1.239843	2.068303	-0.091588	H	3.618140	1.635694	4.672222
C	-2.378899	2.765790	-0.850955	H	2.552863	0.301189	4.191975
C	-3.318836	3.627545	0.015535	H	4.360184	2.901513	1.221914
C	-2.598305	4.847593	0.603970	H	5.390971	1.774257	2.134757
C	-4.534020	4.054162	-0.817228	H	4.652445	3.159203	2.949484
C	-1.637020	0.954028	0.903192	H	1.012974	0.000359	1.986248
C	-3.395705	-0.571064	1.706426	H	0.792499	-3.389078	2.034103
C	-4.916523	-0.748431	1.520581	H	-0.132836	-1.870518	1.994228
C	-5.346714	-1.410184	0.196542	H	0.271054	-1.916099	-0.573945
C	-4.896546	-0.609522	-1.019774	H	-1.483683	-4.023132	0.721860
C	-2.688178	-1.924157	1.494434	H	0.708346	1.996779	-0.903493
C	-1.283313	-3.264566	-0.038887	H	-2.027118	-1.312992	-0.348967
C	-1.625810	-3.796328	-1.430987	H	1.923677	-3.591804	-0.230349
C	0.187553	-2.825539	0.043737	H	-0.961237	-4.620098	-1.698202
C	0.624893	-2.460181	1.479260	H	-1.507353	-3.001886	-2.178055
C	1.921120	-1.698289	1.347072	H	-2.661018	-4.157562	-1.455375
C	3.012911	0.492380	1.444064	H	-3.193371	-0.309421	2.748875
C	3.260472	1.543788	2.533238	H	-5.397649	0.231120	1.607396
C	4.487469	2.395726	2.181631	H	-5.271425	-1.371436	2.345610

C	3.435627	0.877897	3.902643	H	-3.538138	0.874912	0.152990
C	2.639937	1.109667	0.083841	H	-6.442065	-1.453123	0.172842
N	-0.210156	1.564966	-1.006367	H	-4.980821	-2.440007	0.149031
N	-2.903201	0.501793	0.850345	H	-3.761723	-0.758330	-2.681025
N	-4.295504	-1.306236	-2.016093	H	-3.920522	-2.222652	-1.812791
N	-2.123689	-2.112692	0.274655	H	-0.713776	2.800087	0.527297
N	1.922878	-0.422576	1.791978	H	-1.907888	3.413393	-1.601535
N	2.589499	0.213129	-0.925376	H	-2.955634	2.022945	-1.411119
O	-0.809633	0.508705	1.707560	H	-3.686985	3.018589	0.852126
O	2.314616	2.297306	-0.051630	H	-1.760034	4.568601	1.252482
O	-5.067033	0.612408	-1.078603	H	-2.204141	5.487682	-0.195862
O	-2.732321	-2.770643	2.381227	H	-3.287405	5.451769	1.204489
O	1.013340	-3.847043	-0.484409	H	-5.220432	4.668220	-0.222901
O	-1.420025	-0.171572	-1.834707	H	-5.080147	3.179300	-1.182319
O	2.896507	-2.201465	0.751093	H	-4.219676	4.650953	-1.683559
H	2.979797	0.326045	-4.286957	H	5.998832	1.334768	-0.722041
H	3.223468	-1.092872	-3.281535	H	6.632073	-1.602517	0.083725
H	4.735941	1.535432	-2.782854	H	7.781862	-0.263086	0.287286
H	5.294747	0.317345	-3.922702	H	6.300851	-0.299381	1.237203

Supplementary Table 25 | Atomic coordinates (Å) of **1T-b-4** obtained at the B3LYP-D3BJ/6-31G(d) level of theory in the gas phase.

C	-5.614072	-2.535517	0.239638	H	-5.722417	-0.577701	2.135619
C	-4.641352	-2.176342	1.324243	H	-1.974243	1.948740	2.504655
C	-4.820585	-1.189950	2.208468	H	-0.777906	-0.071980	3.530744
C	-3.905540	-0.861185	3.356215	H	-1.306998	-1.027275	2.167129
C	-3.364019	0.581805	3.356847	H	-3.272151	0.071300	0.615848
C	-2.274762	0.914256	2.319995	H	-3.897876	1.099597	-1.411004
C	-1.050243	-0.007167	2.469009	H	-2.010271	3.378802	-2.151283
C	0.242169	0.328577	1.725280	H	-2.393738	1.851335	-4.106737
C	1.530686	1.988053	0.404740	H	-4.143225	1.973675	-3.867138
C	2.719377	2.282887	1.332340	H	-3.215999	3.404113	-4.348676
C	3.875338	3.076970	0.692027	H	-5.060301	3.333083	-1.822709
C	3.453917	4.504131	0.318460	H	-4.112183	4.716451	-2.386214
C	5.076080	3.088949	1.646062	H	-3.900603	4.126842	-0.730749
C	1.778327	1.020737	-0.773391	H	-0.969791	0.928155	-1.920125
C	3.257223	-0.668266	-1.788310	H	-1.300337	-2.347333	-2.795441
C	4.697830	-1.181593	-1.587266	H	-0.144728	-1.080175	-2.320416
C	4.895234	-2.144947	-0.400179	H	-0.777537	-1.653412	0.120208
C	4.519517	-1.503948	0.930606	H	0.631595	-3.759630	-1.551360
C	2.287916	-1.865174	-1.857116	H	-0.452181	2.165581	1.117263
C	0.536721	-3.134498	-0.660089	H	1.627711	-1.469737	0.045960
C	0.665184	-3.980629	0.607022	H	-2.672600	-2.847331	-0.736522
C	-0.808863	-2.392945	-0.696149	H	1.600859	-4.552163	0.590494
C	-1.036507	-1.625557	-2.015561	H	-0.169939	-4.679730	0.684234
C	-2.197156	-0.691806	-1.773556	H	0.657571	-3.334556	1.493642
C	-2.930605	1.609203	-1.384471	H	3.183432	-0.191336	-2.769654
C	-2.975757	2.868713	-2.258927	H	5.364208	-0.321126	-1.468899
C	-4.076085	3.815995	-1.763776	H	4.984159	-1.704833	-2.503199

C	-3.189912	2.499968	-3.730894	H	3.564260	0.415357	0.015544
C	-2.542016	1.905896	0.073578	H	5.957189	-2.412448	-0.346304
N	0.354420	1.541886	1.158250	H	4.335985	-3.070732	-0.564195
N	2.923268	0.314874	-0.764202	H	3.262022	-1.720790	2.492732
N	3.728464	-2.235321	1.754521	H	3.201969	-3.006227	1.366835
N	1.610722	-2.147553	-0.714486	H	1.206201	2.914776	-0.076002
N	-1.947547	0.633599	-1.859628	H	2.325164	2.863592	2.176171
N	-2.793555	0.904802	0.949780	H	3.094211	1.346440	1.757203
O	0.939618	0.912344	-1.676884	H	4.188155	2.566686	-0.228775
O	-1.931029	2.937316	0.388697	H	4.294786	5.054065	-0.118542
O	4.913562	-0.371665	1.227853	H	2.637300	4.523953	-0.412154
O	2.231167	-2.532927	-2.884258	H	3.121281	5.055269	1.207499
O	-1.858389	-3.319458	-0.467401	H	5.411588	2.069748	1.859512
O	1.150438	-0.519847	1.688161	H	4.811333	3.571416	2.596123
O	-3.303452	-1.138499	-1.414947	H	5.914784	3.646351	1.213330
H	-4.194499	1.283545	3.212026	H	-3.734780	-2.781726	1.381004
H	-2.940526	0.797609	4.346066	H	-6.502572	-1.895766	0.275344
H	-4.464429	-0.994540	4.293920	H	-5.155413	-2.428609	-0.748434
H	-3.080713	-1.582640	3.395013	H	-5.941817	-3.578495	0.341942
